# Targeted CENH3 protein depletion in egg cells enables highly efficient haploid induction

**DOI:** 10.1016/j.xplc.2026.101837

**Published:** 2026-03-30

**Authors:** Saravanakumar Somasundaram, Seda Yaşar, Jörg Fuchs, Maria Cuacos, Julian Claassen, Oda Weiss, Andriy Kochevenko, Jonathan C. Lamb, Tengyu Li, Niklas Capdeville, Holger Puchta, Andreas Houben

**Affiliations:** 1 Leibniz Institute of Plant Genetics and Crop Plant Research (IPK) Gatersleben, Corrensstrasse 3, 06466 Seeland, Germany; 2 Joseph Gottlieb Kölreuter Institute of Plant Sciences (JKIP) – Molecular Biology, Karlsruhe Institute of Technology (KIT), 76131 Karlsruhe, Germany; 3 Bayer U.S. – Crop Science, 700 Chesterfield Parkway West, Chesterfield, MO 63017, USA; 4 Martin Luther University Halle Wittenberg, Institute of Agricultural & Nutritional Sciences, 06120 Halle (Saale), Germany

**Keywords:** ALFA-tag, centromere, haploid induction, uniparental genome elimination, targeted protein degradation, nanobody, *in locus* protein tagging, gene targeting

## Abstract

Doubled haploid technology is a transformative tool for accelerating plant breeding by enabling the rapid development of homozygous lines. While manipulation of the centromere-specific histone H3 variant CENH3 has been shown to induce haploids in *Arabidopsis* and selected crop species, a broadly applicable approach remains elusive. The prevailing hypothesis is that CENH3 asymmetry between parental genomes during early embryonic development leads to the selective elimination of parental chromosomes with reduced CENH3 and consequently weaker centromeres. We experimentally validate this hypothesis by depleting EYFP- or ALFA-tagged CENH3 using ubiquitin-mediated proteasomal degradation specifically in the egg cell prior to fertilization with wild-type pollen. This approach consistently generated paternal wild-type haploids, with induction frequencies of up to 57% among progeny derived from egg cells containing constructs for CENH3 depletion. We further enhanced the system by incorporating a plant-derived E3 ubiquitin ligase for efficient CENH3 degradation and a fluorescent seed marker for rapid haploid identification. This approach also proved effective in *in-locus* ALFA-tagged CENH3 lines generated by gene targeting. Furthermore, the plant-derived E3 ubiquitin ligase successfully degraded CENH3 from tomato. Thus, this gametic CENH3-depletion system establishes a rational, modular framework for engineering haploid inducers and provides a potentially universal platform for haploid induction across diverse crop species.

## Teaser

This study demonstrates that targeted depletion of the centromeric protein CENH3 in egg cells can efficiently induce haploid plants by eliminating maternal chromosomes during early embryogenesis. Using a ubiquitin-mediated degradation system, the authors achieved haploid induction rates of up to 57% and further enhanced the system by replacing animal-derived E3 ligases with a plant-derived E3 ligase. This approach provides a modular and potentially universal strategy to accelerate plant breeding across diverse crop species.

## Introduction

Haploid inducers are specialized genotypes that facilitate haploid production *in planta* through intra-specific hybridization, enabling haploids to be directly obtained from seeds (Widiez, 2021). This approach can be broadly classified into (i) centromere-based and (ii) non-centromere-based methods. Centromere-based methods involve the manipulation of the centromere-specific histone H3 variant (CENH3) or kinetochore proteins, such as KNL2, to alter chromosome segregation (Ravi and Chan, 2010; Lv et al., 2020; Wang et al., 2021; Ahmadli et al., 2023). In contrast, non-centromere-based methods rely on mutations in genes such as *MTL*, *DMP*, and *Kokopelli*, which disrupt various reproductive processes to induce haploids (Kelliher et al., 2017; Zhong et al., 2019; Jacquier et al., 2023). Generally, centromere-based methods can induce both paternal and maternal haploids in monocots and dicots. In contrast, non-centromere-based methods induce haploids containing maternal chromosomes and are often specific to either monocots or dicots (Quiroz et al., 2024). Among these systems, CENH3 manipulation has proven the most efficient *in vivo* method for haploid induction, especially in *Arabidopsis thaliana*, as reviewed by Quiroz et al. (2024).

In *Arabidopsis*, homozygous *cenh3* null mutants expressing a chimeric “GFP-tailswap” protein induced up to 45% paternal haploids and 5% maternal haploids when outcrossed with wild-type plants (Ravi and Chan, 2010). Other CENH3 manipulations, such as point mutations, heterologous CENH3 complementation, and inactivation of kinetochore proteins such as KNL2, have also enabled haploid induction (Karimi-Ashtiyani et al., 2015; Kuppu et al., 2015, 2020; Maheshwari et al., 2015; Ahmadli et al., 2023). Environmental factors, particularly temperature, have been shown to further modulate haploid induction frequency (Ahmadli et al., 2023; Jin et al., 2023; Wang et al., 2023).

Despite success in *Arabidopsis*, translating centromere-based haploid induction to crop species has proven challenging. In maize, plants heterozygous for a *cenh3*-null mutation produced 5% paternal haploids upon outcrossing (Wang et al., 2021). Under similar conditions, no haploids were induced in wheat, and an extremely low frequency (0.4%) was observed in *Arabidopsis* (Lv et al., 2020; Marimuthu et al., 2021). Alternatively, a specific type of mutation, namely a restored frameshift allele, was effective for haploid induction at frequencies of up to 8% in wheat (Lv et al., 2020). A similar kind of allele resulted in haploid induction in broccoli (Han et al., 2024). RNAi-mediated downregulation of CENH3 triggered haploid production in onion and cotton but not in maize or *Arabidopsis* (Kelliher et al., 2016; Gao et al., 2020; Ahmadli et al., 2023; Manape et al., 2024). Thus, although CENH3 manipulation enables haploid induction across various species, no single strategy can be universally applied across all plant species.

Our earlier work demonstrated targeted EYFP-CENH3 degradation using engineered E3 ligases encoded by the paternal genome, which eliminates maternal chromosomes in *Arabidopsis* (Demidov et al., 2022). However, this approach suffers from critical limitations: it requires transgenes in both parents, produces transgenic haploids unsuitable for breeding, and shows very low efficiency compared with established CENH3 manipulation methods in *Arabidopsis*.

A better understanding of the mechanistic basis would enable the rational design of a universal centromere-based haploidization strategy. Cumulative insights from previous studies indicate that CENH3 imbalance between parental genomes during early embryogenesis results in uniparental genome elimination (Sanei et al., 2011; Raychaudhuri et al., 2012; Marimuthu et al., 2021; Wang et al., 2021; Comai and Marimuthu, 2025). Uniparental gametic CENH3 depletion represents a promising strategy to achieve CENH3 imbalance between parental genomes. However, whether targeted gametic CENH3 depletion can successfully induce haploids in plants remains unexplored.

Here, we demonstrate that egg-cell-specific depletion of epitope-tagged CENH3 enables efficient paternal haploid induction in *A. thaliana*. We developed a strategy to specifically deplete EYFP- or ALFA-tagged CENH3 in egg cells using engineered E3 ligases, producing non-transgenic haploids through outcrossing with wild-type plants. To address concerns associated with the use of animal-derived E3 ubiquitin ligases in crop breeding, we successfully replaced them with a plant-derived alternative. This approach achieved haploid induction frequencies of up to 57% in genetic backgrounds previously devoid of haploid induction capability and enhanced the efficiency of existing haploid inducers to up to 72%. We confirmed the strategy’s effectiveness by integrating the epitope tag directly into the native CENH3 locus via gene targeting (GT). Moreover, the engineered E3 ligases successfully degraded tagged CENH3 from tomato, demonstrating potential cross-species applicability. These findings establish gametic CENH3 depletion as a systematic, scalable, and potentially universal strategy for centromere-based haploid induction in crop improvement.

## Results

### *EYFP-gCENH3* induces varying levels of haploids

Previously, we utilized a *cenh3* null mutant complemented with an EYFP-tagged CENH3 transgene, *EYFP-gCENH3*^*cenh3-1*^, for targeted removal of the EYFP-CENH3 protein in *Arabidopsis* (Demidov et al., 2022). To test whether the gametic depletion of CENH3 results in haploid induction, we utilized *EYFP-gCENH3*^*cenh3-1*^ as a test genotype. We found that *EYFP-gCENH3*^*cenh3-1*^ has a background haploid induction activity ranging from 5.54% to 20.34% (Supplemental Table 1). A similar genotype from an independent complementation experiment also resulted in haploid induction frequencies ranging from 6.25% to 14.81% (Supplemental Table 1). However, no haploids were identified in the control cross in the previous study (Demidov et al., 2022). We assume that the low sample size in the control cross in the previous study underlies these contradictory observations.

**Figure 1:**
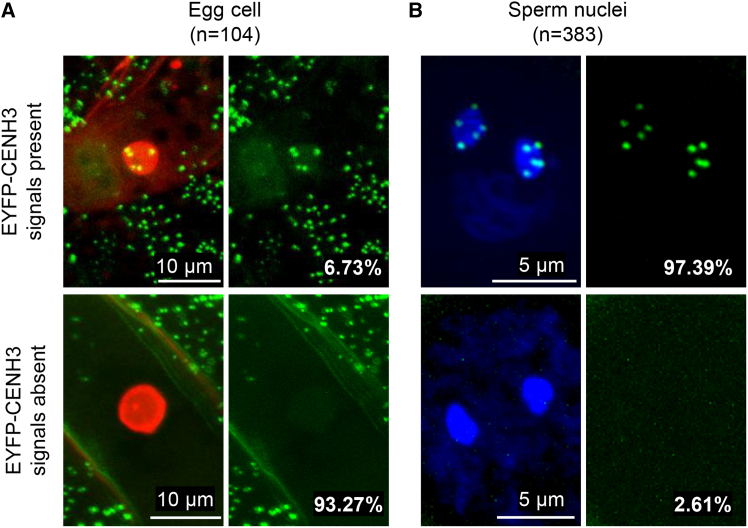
*EYFP-gCENH3*^*cenh3-1*^ exhibits different levels of EYFP-CENH3 in male and female gametes in *A. thaliana.* **(A)** Representative egg cells labeled with histone H2B-tdTomato (red), showing the presence or absence of EYFP-CENH3. **(B)** Representative sperm nuclei stained with DAPI (blue), showing the presence or absence of EYFP-CENH3. *n* indicates the number of ovules or pollen analyzed. The frequency of gametes with or without EYFP-CENH3 is given for each scenario.

Marimuthu et al. (2021) suggested that removal of the GFP-tailswap variant in the egg cell is a potential reason for haploid induction. To check whether EYFP-CENH3 is also absent in the egg cell, we examined the egg cells of EYFP-gCENH3^cenh3-1^. Only 6.73% of egg cells displayed EYFP-CENH3 foci, while all other egg cells showed no detectable EYFP-CENH3 fluorescence (Figure 1A). In contrast, EYFP-CENH3 is detectable in the sperm nuclei of most pollen (Figure 1B). Although most egg cells lack detectable EYFP-CENH3, this results in only up to 19.3% haploids. Therefore, we assumed that EYFP-CENH3 is not completely removed from egg cells and may still be present at levels below the detection limit of microscopy. If this is the case, complete removal of EYFP-CENH3 in gametes should enhance haploid induction frequency.

### Egg-cell-specific depletion of the EYFP-CENH3 protein results in shriveled seeds

**Figure 2:**
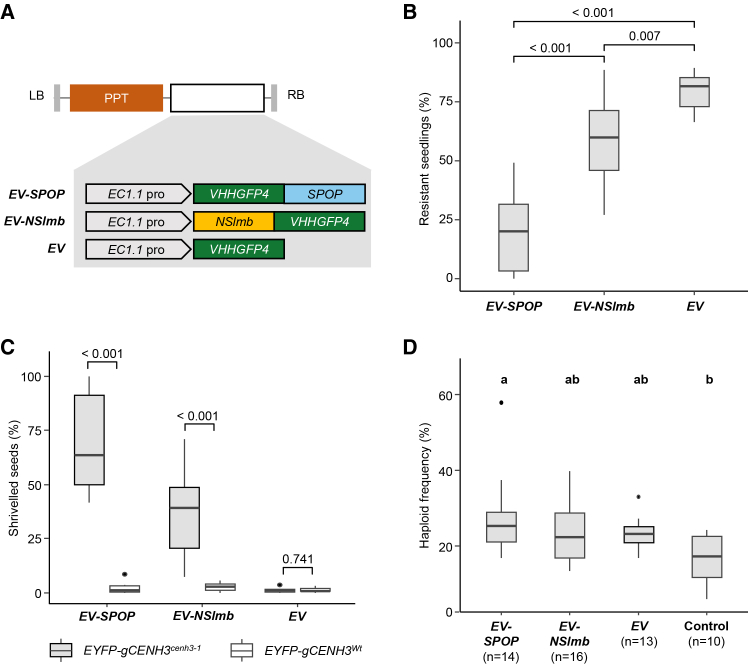
Egg-cell-specific degradation of EYFP-CENH3 results in shriveled seeds and increased haploid induction frequency. **(A)** Schematic representation of different constructs (*EV-SPOP*, *EV-NSlmb*, and *EV*) used for egg-cell-specific degradation of EYFP-CENH3. *PPT*, *phosphinothricin*. **(B)** Frequency of *PPT*-resistant seedlings obtained from T2 seeds of 10 individual T1 *EYFP-gCENH3*^*cenh3-1*^ plants for the different constructs shown in **(A)**. Comparisons are based on adjusted *p* values calculated using the Holm–Sidak method for three pairwise comparisons. **(C)** Frequency of shriveled seeds after self-pollination of 10 different T1 plants from each genotype (*EYFP-gCENH3*^*cenh3-1*^ and *EYFP-gCENH3*^*Wt*^) transformed with the constructs shown in **(A)**. *p* values represent the significance of differences between genotypes based on a two-sample *t*-test. **(D)** Haploidization frequency of independent *EYFP-gCENH3*^*cenh3-1*^ T1 plants hemizygous for the different constructs shown in **(A)**, after pollination with *gl1-1* pollen. *EYFP-gCENH3*^*cenh3-1*^ plants without any of the constructs were used as controls. *n* indicates the number of individual T1 plants evaluated for each construct and individual plants used as controls. *p* values were calculated using the Holm–Sidak method for six pairwise comparisons. A significance threshold of *p* < 0.05 was applied, and different letters denote statistically significant differences in haploid induction frequency between constructs. Detailed data, including the total number of plants screened, haploid counts, and haploid frequencies for each individual cross, are provided in Supplemental Table 9.

To test our hypothesis, we employed engineered E3-ligase-based targeted depletion of EYFP-CENH3 in egg cells. These E3 ligases recognize EYFP-CENH3 through a fused GFP-specific nanobody, subsequently marking the protein for ubiquitin-mediated proteasomal degradation via lysine polyubiquitination. The E3 ligases were expressed under the control of the *EC1.1* promoter (Sprunck et al., 2012) to degrade EYFP-CENH3 specifically in egg cells. Three different constructs, namely *EV-SPOP*, *EV-NSlmb*, and *EV*, were utilized for this experiment (Figure 2A). The first two bear the E3 ligases *SPOP* (Shin et al., 2015) and *NSlmb* (Caussinus et al., 2011), respectively, fused to a GFP nanobody (*VHHGFP4*) (Rothbauer et al., 2006), and the third one (*EV*) carries the GFP-nanobody alone. All three constructs were used to transform *EYFP-gCENH3*^*cenh3-1*^ plants.

To recover homozygous transgenic lines for these constructs, T2 seeds from 10 independent T1 plants per construct were used for *phosphinothricin* (*PPT*) selection. The frequency of *PPT*-resistant T2 seedlings was significantly lower in *EV-SPOP* and *EV-NSlmb* compared with *EV* (Figure 2B). Upon further investigation, a high number of non-viable, shriveled T2 seeds were derived from *EV-SPOP* and *EV-NSlmb* T1 plants (Supplemental Figure 1). In contrast, no shriveled seeds were obtained from *EV* T1 plants (Supplemental Figure 1). This observation raised the question of whether expression of E3 ligases itself might affect seed development.

To test this possibility, *EYFP-gCENH3*^*Wt*^ plants were transformed with the constructs described above. Seeds from a few siliques of T1 plants from both genotypes, *EYFP-gCENH3*^*cenh3-1*^ and *EYFP-gCENH3*^*Wt*^, expressing *EV-SPOP*, *EV-NSlmb*, or *EV* were pooled and examined for the prevalence of shriveled seeds. The frequency of shriveled seeds differed significantly between the two genotypes for *EV-SPOP* and *EV-NSlmb* (Figure 2C). In the case of *EV*, no shriveled seeds were observed in either genotype (Figure 2C). This suggests that shriveled seeds were generated only when *EV-SPOP* or *EV-NSlmb* was expressed in the *EYFP-gCENH3*^*cenh3-1*^ background, likely due to genomic instability during early embryogenesis resulting from reduced abundance of EYFP-CENH3 caused by targeted degradation in egg cells.

### Hemizygous T1 plants carrying transgene cassettes with *EV-SPOP* lead to an increased frequency of haploids

The isolation of homozygous transgenic lines through segregation analysis of *PPT* resistance was hindered by the prevalence of shriveled seeds among T2 progeny from plants containing the *EV-SPOP* and *EV-NSlmb* constructs. To investigate whether egg-cell-specific degradation of EYFP-CENH3 could enhance haploid induction frequency, *EYFP-gCENH3*^*cenh3-1*^ T1 plants hemizygous for the above constructs were used as mother plants and crossed with *gl1-1* fathers in order to use the glabrous phenotype (trichome-less leaves) as a marker for haploid screening (Kuppu et al., 2015). The *EYFP-gCENH3*^*cenh3-1*^ genotype without a CENH3-degradation construct served as a control.

Evaluation of the offspring from these crosses showed that the *EV-SPOP* construct significantly increased haploid frequency (median 25.17%) compared with the control (median 17.11%), whereas the other constructs were not significantly different from the control (Figure 2D). Although not statistically significant, we observed an increase in haploid induction frequency for *EV* plants (median 23.08%) compared with that of the control. Also, there was no significant difference between T1 plants carrying E3-ligase-bearing constructs (*EV-SPOP* and *EV-NSlmb*) and those with *EV*. Consequently, it remained unclear whether the increase in haploid induction frequency was attributable to the effect of E3 ligases or to the nanobody alone. We hypothesized that variability in the control group contributed to the noise in our data, leading to these ambiguous results. Therefore, isolating a tagged CENH3 line devoid of background haploid induction became necessary to obtain more precise conclusions.

### Identification of a non-haploid-inducing ALFA-CENH3 *Arabidopsis* line

**Figure 3:**
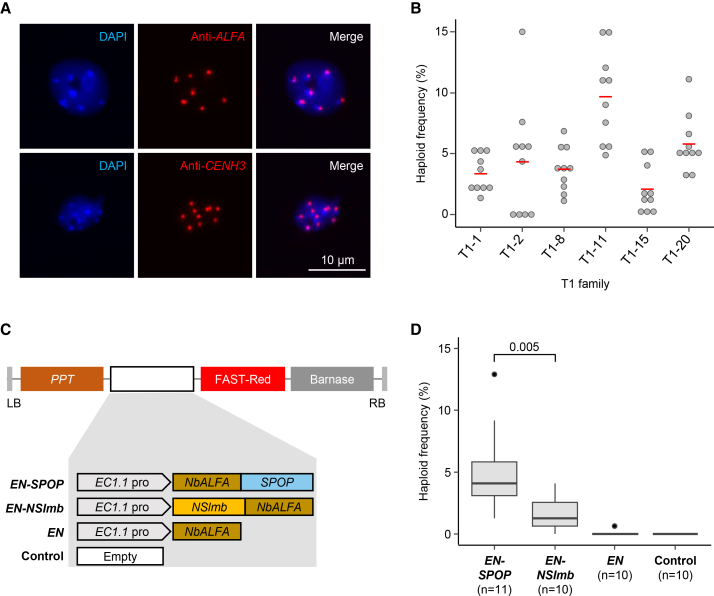
Egg-cell-specific degradation of ALFA-CENH3 by different E3 ligases results in haploid induction. **(A)** Immunolabeling of leaf nuclei from *ALFA-gCENH3*^*cenh3-1*^ plants with anti-*ALFA* and anti-*CENH3* antibodies. Immunosignals are shown in red. Nuclei were stained with DAPI (blue). **(B)** Haploid induction frequencies of 10 females from different *ALFA-gCENH3*^*cenh3-1*^ T1 families after outcrossing with *gl1-1* fathers. Gray dots indicate haploid induction frequencies of T2 samples from the corresponding T1 families. Red lines indicate the median haploid induction frequency of the corresponding T1 family. Detailed data, including the total number of plants screened, haploid counts, and haploid frequencies for each individual cross, are provided in Supplemental Table 10. **(C)** Schematic representation of different constructs (*EN-SPOP*, *EN-NSlmb*, *EN*, and Control) used for egg-cell-specific degradation of ALFA-CENH3. *PPT*, *phosphinothricin*; FAST-Red, fluorescent seed marker; *Barnase*, intronized *barnase* gene expressed under the control of the *A9* promoter for male sterility. **(D)** Haploid induction frequency of independent *ALFA-gCENH3*^*cenh3-1*^ T1 plants hemizygous for the different constructs shown in **(C)** after pollination with pollen from *gl1-1* plants. *n* indicates the number of individual T1 plants evaluated for each construct and individual plants used as controls. *p* values are based on a two-sample *t*-test. Detailed data, including the total number of plants screened, haploid counts, and haploid frequencies for each individual cross, are provided in Supplemental Table 11.

To generate a tagged CENH3 variant without background haploid induction, we replaced the EYFP tag (26.7 kDa) with the smaller ALFA protein tag (1.9 kDa) (Gotzke et al., 2019), assuming that the reduced size of the ALFA-tag might eliminate background haploid induction activity. We successfully complemented the *cenh3* null mutant with an *ALFA*-tagged genomic CENH3 transgene, *ALFA-gCENH3* (Supplemental Figure 2). Indirect immunostaining of nuclei with an *ALFA*-specific nanobody and an *Arabidopsis* CENH3-specific antibody revealed centromere-like signals in interphase nuclei (Figure 3A). To assess haploid induction frequency, plants from six independent *ALFA-gCENH3*^*cenh3-1*^ T1 families were pollinated with pollen from *gl1-1* plants. Ten different female plants from each T1 family were used. Haploid frequencies varied widely both within and between T1 families, with an overall haploid frequency ranging from 0% to 15.15% (Figure 3B). Thus, despite the small size of the ALFA-tag, *ALFA-gCENH3*^*cenh3-1*^ plants still induced haploids after outcrossing with wild-type plants. The observed variability suggests that haploid induction ability and efficiency may be influenced by transgene expression levels or positional effects rather than by the ALFA-tag itself.

However, we identified one T1 family, T1-2, in which 4 of 10 T2 plants lacked haploids among the evaluated progeny (Figure 3B). For further analysis, we focused on 1 of these 4 T2 plants, namely T1-2-23. Ten individual T3 plants derived from selfing of T1-2-23 were used as females and crossed with *gl1-1* plants, and the progeny were analyzed for haploid plants. None of the 3,009 assessed F1 plants displayed the glabrous phenotype. Therefore, T3 plants derived from T1-2-23 were used for further transformation with different constructs designed for egg-cell-specific degradation of ALFA-CENH3.

### Egg-cell-specific depletion of ALFA-CENH3 leads to haploid induction

For the egg-cell-specific degradation of ALFA-CENH3, four distinct constructs were developed: *EN-SPOP*, *EN-NSlmb*, *EN*, and Control. The *EN-SPOP* and *EN-NSlmb* constructs contain an anti-ALFA nanobody (*NbALFA*) fused to E3 ligases *SPOP* and *NSlmb*, respectively (Figure 3C). The *EN* construct includes only the nanobody (*NbALFA*), while the control construct consists of an empty module. Each construct also integrates a male sterility module (intronized *Barnase*; Hartley, 1988) expressed under the *A9* promoter (Paul et al., 1992) to facilitate crossing, as well as a FAST-Red fluorescent seed marker (Shimada et al., 2010). The fluorescent seed marker was assessed for its suitability in identifying haploid progeny.

*ALFA-gCENH3*^*cenh3-1*^ T1 plants hemizygous for any of the above-mentioned constructs were crossed as females with *gl1-1* fathers, and the progeny were evaluated for haploid induction. Crosses with the control construct showed no induction of haploids, confirming that the background does not promote haploid formation (Figure 3D). The nanobody-only construct (*EN*) similarly failed to induce haploids across most lines, with one exception displaying a negligible haploid induction frequency (0.64%) (Figure 3D), suggesting that the anti-*ALFA* nanobody alone is insufficient for effective haploid induction. In contrast, both E3-ligase-fused constructs, *EN-SPOP* and *EN-NSlmb*, resulted in haploid induction, with *EN-SPOP* (median 4.11%) achieving a significantly higher induction frequency of haploids than *EN-NSlmb* T1 plants (1.31%) (Figure 3D). Thus, E3-ligase-mediated degradation of CENH3 in egg cells is effective in inducing paternal haploids when tested in a genetic background confirmed to lack intrinsic haploid-inducing activity. This experimental design ensures that any observed haploid induction can be unambiguously attributed to the introduced degradation module rather than to confounding effects from the tagged CENH3 line itself.

### Seed marker aids in rapid haploid screening and transgene cassette inheritance tracking

Most previous studies on CENH3-based haploid induction have relied on the glabrous phenotype of the *gl1-1* mutant as a marker for identifying haploids (Kuppu et al., 2015, 2020; Ahmadli et al., 2023; Wang et al., 2023). However, because glabrousness is a recessive trait, its use limits haploid screening to crosses involving *gl1-1* or similar mutants. To address this constraint, we incorporated the dominant FAST-Red fluorescent seed marker (Shimada et al., 2010) into the transgene cassettes for CENH3 depletion. Previous research has shown that FAST-Red enables highly accurate identification of haploid seeds, particularly in *DMP* mutant backgrounds (Zhong et al., 2020).

F1 seeds obtained by crossing *EN-SPOP* with *gl1-1* fathers can be classified into four distinct classes: Class 1, non-fluorescent and morphologically normal; Class 2, bright, uniform red fluorescence; Class 3, faint, uniform red fluorescence; and Class 4, shriveled and collapsed seeds with non-uniform fluorescence (Supplemental Figure 3A and 3B). Class 1 seeds most likely represent seeds originating from gametes lacking the transgene cassette and are therefore wild type, accounting for 38.81%–45.53% (Supplemental Table 2). The frequency of fluorescent seeds (Classes 2 and 3) in *EN-SPOP* crosses was markedly lower, ranging from 12.77% to 19.40% (Supplemental Table 2). This reduction suggests that segregation of the *EN-SPOP* transgene cassette is impaired. Most Class 4 seeds from *EN-SPOP* crosses exhibited residual red fluorescence (Supplemental Figure 3B), indicating that gametes carrying the *EN-SPOP* construct frequently gave rise to non-viable seeds. This pattern of seed lethality is a well-known consequence of chromosome elimination. In F1 seeds obtained by crossing *EN* T1 plants with *gl1-1*, the majority of seeds belonged to either Class 1 or Class 2 (Supplemental Table 2).

To determine the ploidy status of each seed category, we conducted flow cytometric analysis on nuclei isolated from F1 seeds. All Class 1 seeds were diploid, whereas Class 2 seeds predominantly consisted of diploids, along with a notable proportion of aneuploids and some mixoploids (Supplemental Table 3). Strikingly, Class 3 seeds, which exhibited faint red fluorescence, showed a high frequency of haploids (72.5%), in addition to diploids, aneuploids, and mixoploids. Class 4 seeds did not allow the isolation of nuclei suitable for ploidy determination, likely due to aborted embryo development.

To compare the accuracy of the glabrous phenotype and faint seed fluorescence (Class 3) in identifying haploids, seeds from different classes were germinated and screened. Glabrous plants were recovered exclusively from Class 3 seeds, consistent with our ploidy analysis (Supplemental Table 2). The accuracy of Class 3 seeds in predicting haploids was 92.11%, compared with 97.22% for the glabrous phenotype (Supplemental Table 4). Despite the lower accuracy than that of the glabrous phenotype, the >90% accuracy of Class 3 fluorescence provides a reliable alternative for rapid haploid screening, reducing dependency on the glabrous phenotype.

In addition, we used the FAST-Red seed marker to trace the gametic origin of haploids. Our results showed that haploids occurred exclusively among fluorescent seeds, indicating that they originated from egg cells carrying the transgene cassette with the degradation module, *EN-SPOP* (nanobody with an E3 ligase). In contrast, the *EN* transgene cassette without the degradation module (nanobody without an E3 ligase) showed normal segregation, with no haploid (glabrous) progeny observed among fluorescent seeds (Supplemental Table 2). This confirms that egg-cell-specific CENH3 depletion caused by the degradation module is responsible for haploid induction.

### Genome elimination occurs exclusively in the embryo

To understand the differences between bright (Class 2) and faintly fluorescent (Class 3) *EN-SPOP* F1 seeds, we analyzed them using confocal microscopy. In Class 2 seeds, FAST-Red fluorescence was predominantly detected in the embryo and in a single endosperm layer surrounding the embryo (Supplemental Figure 4). In Class 3 seeds, however, fluorescence was confined solely to the endosperm (Supplemental Figure 4). Ravi et al. (2014) reported non-uniform fluorescence patches in haploid seeds from GFP-tailswap mothers, indicating genome elimination in the endosperm. In contrast, we observed uniform fluorescence throughout the endosperm. This difference demonstrates that our approach utilizing the *EC1.1* promoter, restricts CENH3 degradation specifically to the egg cell, thereby confining genome elimination to the embryo rather than extending it to the endosperm.

### Accurate assessment of haploid induction requires accounting for transgene transmission

Given that the degradation module (*EN-SPOP*) functions at the gametic level, the predominance of progeny from non-fluorescent Class 1 F1 seeds lacking the transgene (Supplemental Table 2) obscures accurate evaluation of haploid induction efficiency. To address this, we focused specifically on progeny derived from transgenic gametes to determine haploid induction frequency.

The observed haploid induction frequency, calculated as the percentage of haploid plants among all germinated seedlings, ranged from 2.18% to 3.57% (Table 1). This metric underestimates the true performance of the degradation module because it encompasses progeny from both transgenic and non-transgenic gametes, although only the former possess haploid induction capability. Restricting the analysis to seedlings originating from transgenic gametes (seed classes 2, 3, and 4) provided an accurate measure of the module’s performance.

**Table 1:** Observed and effective haploid induction frequencies of three independent *EN-SPOP* T1 lines.

Cross	T1 replicate	Total germinated seedlings	Seedlings from fluorescent seeds	No. of glabrous plants	Haploids based on flow cytometry	Observed haploid frequency (%)	Effective haploid frequency (%)
*EN-SPOP* × *gl1-1*	1	275	24	6	6	2.18	25.00
	2	448	28	17^a^	16	3.57	57.14
	3	465	36	13	13	2.80	36.11

Seedlings germinated from seed classes 2, 3, and 4 were considered progeny from fluorescent seeds. Observed haploid induction frequency represents the percentage of haploids among total germinated seedlings. Effective haploid induction frequency represents the percentage of haploids among progeny derived specifically from fluorescent seed classes.

a One glabrous seedling was confirmed to be diploid by flow cytometry and was excluded from frequency calculations.

The haploid induction frequency, calculated as the percentage of haploid plants among seedlings derived from these seeds, corresponds to the effective haploid induction frequency. Under this refined calculation, the effective haploid induction frequency ranged from 25% to 57.14% (Table 1), demonstrating that the *EN-SPOP* module is capable of inducing haploids at a very high frequency.

### The plant E3 ligase EL5 enhances haploid induction frequency compared with SPOP when combined with EYFP-CENH3 but not with ALFA-CENH3

Haploid induction performance differed between the E3 ligases *SPOP* and *NSlmb* (Figure 3D), both derived from non-plant organisms. We hypothesized that a plant-derived E3 ligase would exhibit enhanced interaction with the plant ubiquitination machinery, thereby improving target protein degradation and the frequency of haploidization. To test this hypothesis, we selected EL5, a well-characterized E3 ligase from *Oryza sativa*. *EL5* encodes a RING-type protein that directly interacts with E2 ubiquitin-conjugating enzymes, promoting efficient substrate ubiquitination (Takai et al., 2002). Katoh et al. (2003) demonstrated that the region spanning amino acids 96–181 is critical for ubiquitination activity.

To assess the functionality of EL5 in mediating CENH3 degradation, we constructed a transgene cassette bearing a degradation module comprising *VHHGFP4* fused to the active domain of EL5 (amino acids 96–181) and histone H2B-mCherry. A transgene cassette without a degradation module was used as the control (Supplemental Figure 5A). Protoplasts isolated from *EYFP-gCENH3*^*cenh3-1*^ plants were transfected with these constructs. Fluorescence microscopy analysis revealed a marked reduction in EYFP-CENH3 signals in protoplasts transformed with the *EL5* degradation module, with signal absence observed in most cells (Supplemental Figures 5B and 5C). In contrast, control protoplasts transformed with H2B-mCherry alone retained detectable EYFP-CENH3 fluorescence in most cells (Supplemental Figures 5B and 5C), confirming that the observed signal reduction was specifically attributable to EL5-mediated CENH3 degradation.

**Figure 4:**
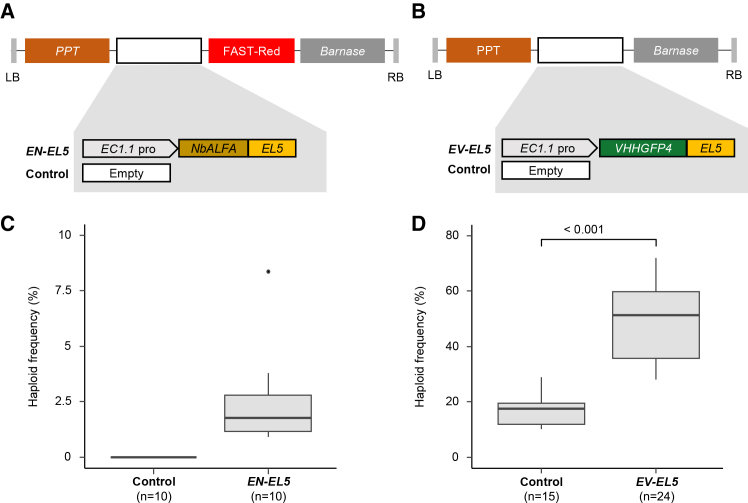
Plant E3 ligase EL5 is more efficient than SPOP only when combined with EYFP-CENH3. **(A)** Schematic representation of the different constructs (*EN-EL5* and control) used for the transformation of *ALFA-gCENH3*^*cenh3-1*^. **(B)** Schematic representation of the different constructs (*EV-EL5* and control) used for the transformation of *EYFP-gCENH3*^*cenh3-1*^. **(C and D)** Haploidization frequency of independent T1 plants hemizygous for the constructs shown in **(A)** and **(B),** after pollination with *gl1-1* pollen. *n* indicates the number of individual T1 plants evaluated for each construct and individual plants used as controls. The *p* value in **(D)** is based on Welch’s *t*-test. Detailed data, including the total number of plants screened, haploid counts, and haploid frequencies for each individual cross, are provided in Supplemental Table 12 for **(C)** and Supplemental Table 13 for **(D)**.

To assess its potential for haploid induction, we engineered two independent degradation modules by fusing a functional *EL5* fragment (amino acids 96–181) to *NbALFA* and *VHHGFP4* and placed both fusions under the control of the egg-cell-specific *EC1.1* promoter. The resulting constructs, *EN-EL5* and *EV-EL5*, were introduced into *ALFA-gCENH3*^*cenh3-1*^ and *EYFP-gCENH3*^*cenh3-1*^ plants, respectively (Figures 4A and 4B). Hemizygous T1 plants carrying either *EN-EL5* or *EV-EL5* were crossed with *gl1-1* males, and the F1 progeny were screened for glabrous individuals as a proxy for haploid induction.

Our results showed that *EN-EL5* is capable of inducing haploids, with a median frequency of 1.76% (Figure 4C). In contrast, T1 lines expressing *EV-EL5* showed a median haploid induction frequency of 51.39%, with values reaching up to 72% (Figure 4D). This represents a significant 2.94-fold increase compared with the control (median 17.5%) (Figure 4D). EL5 (median 51.39%) was more efficient than SPOP (median 25.17%) when combined with EYFP-CENH3. However, this trend was reversed in the context of ALFA-CENH3, where *SPOP* (median 4.11%) outperformed *EL5* (median 1.76%). This indicates that the efficiency of both E3 ligases in terms of haploid induction frequency are differentially influenced by the epitope tag used for CENH3 degradation.

### *EN-SPOP* enables haploid induction in an *in-locus* ALFA-tagged CENH3 line

**Figure 5:**
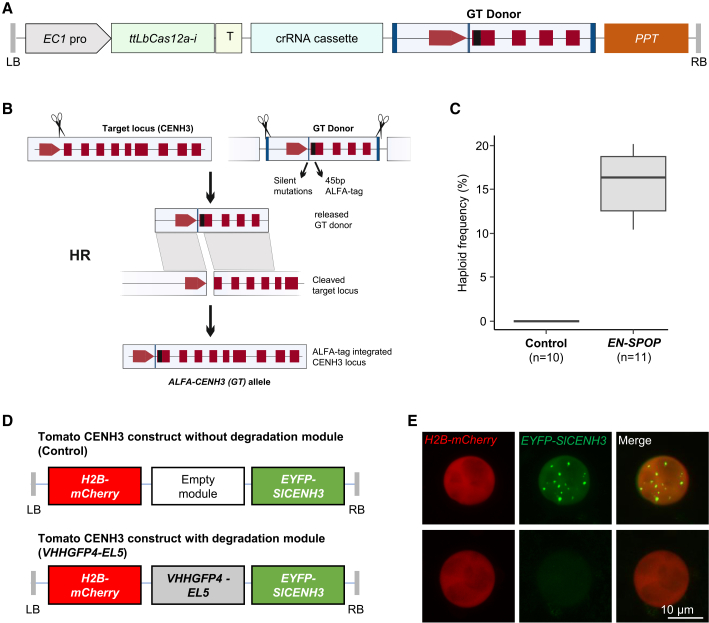
Functionality of degradation modules in the *ALFA-CENH3* (*GT*) background and for tomato CENH3 degradation. **(A)** Schematic representation of the T-DNA construct used for *in planta* gene targeting (*ip*GT). Cas12a expression was driven by an egg-cell-specific promoter (*EC1pro*) using the intronized tt*Lb*Cas12a variant (*ttLbCas12a*-*i*) together with a crRNA cassette targeting *AtCENH3* and a GT donor flanked by target sites for donor excision. The GT donor consists of 5′ and 3′ homology arms and includes the 45-bp ALFA-tag coding sequence, with silent mutations introduced to prevent re-cleavage after successful integration. The phosphinothricin resistance cassette (PPT) was used as a selection marker. **(B)** Overview of the *ip*GT strategy for inserting the ALFA-tag at the wild-type *CENH3* locus. Cas12a induces a double-strand break at the target site within the start codon and, at the same time, excises the GT donor from the vector to be used as a template for homologous recombination (HR). HR results in precise insertion of the ALFA tag immediately downstream of the start codon, generating an ALFA-tagged *CENH3* allele. **(C)** Haploidization frequency of *ALFA-CENH3* (*GT*) plants and independent hemizygous *EN-SPOP* T1 plants in the *ALFA-CENH3* (*GT*) background after pollination with *gl1-1* pollen. *n* indicates either the number of individual T1 plants evaluated for *EN-SPOP* or the number of individual plants used as controls. Detailed data, including the total number of plants screened, haploid counts, and haploid frequencies for each individual cross, are provided in Supplemental Table 14. **(D)** Schematic representation of transgene cassettes with and without the degradation module (*VHHGFP4-EL5*), carrying H2B-mCherry and EYFP-*Sl*CENH3 (tomato), used for transient transformation in *N. benthamiana*. **(E)** Representative nuclei of *N. benthamiana* labeled with histone H2B-mCherry (red) and showing the presence or absence of EYFP-*Sl*CENH3 (green).

The variation in haploid induction efficiency among *ALFA-gCENH3*^*cenh3-1*^ T1 families suggests that variation in the expression level of modified CENH3, due to transgene positional effects, might contribute to haploid induction capacity. To test this hypothesis, we generated *ALFA-CENH3* knock-in plants by targeted integration of an ALFA tag at the endogenous *CENH3* locus using *ttLbCas12a-i*-mediated *in planta* GT (Figure 5A) (Merker et al., 2020; Schindele et al., 2023). The CRISPR-Cas12a cleavage site was positioned within the start codon to induce a double-strand break, facilitating ALFA-tag integration. The GT donor was designed with a 5′ homology arm of approximately 550 bp containing two silent point mutations to prevent re-cleavage after successful integration and a 3′ homology arm of approximately 580 bp flanking the 45-bp ALFA-tag coding sequence (Supplemental Data 1). Within this donor construct, the ALFA-tag was precisely placed in-frame at the N terminus of *At*CENH3 to ensure accurate tagging upon homologous recombination. To enable donor excision, additional CRISPR-Cas12a target sites, including protospacer adjacent motif (PAM) sequences, were placed at both ends of the GT donor (Figure 5B).

Screening of 2400 T2 plants identified one heterozygous plant carrying the correctly targeted ALFA-tag insertion, corresponding to a GT efficiency of 0.04%. From its progeny, plants with a homozygous ALFA tag insertion were identified by PCR and used for further experiments. Molecular analysis confirmed precise integration of the ALFA tag at the target locus (Supplemental Figure 6). This allele of *CENH3* is termed the “*ALFA-CENH3* (*GT*)” allele. Plants homozygous for the *ALFA-CENH3* (*GT*) allele were isolated, and they exhibited no morphological differences compared with wild-type Col-0. Anti-*ALFA* immunostaining confirmed the functionality and centromeric localization of the ALFA-CENH3 protein (Supplemental Figure 7).

To assess haploid induction capacity, homozygous *ALFA-CENH3* (*GT*) plants were crossed with *gl1-1* males, and F1 progeny were screened for glabrous haploid plants. No glabrous plants were detected among the progeny, indicating that *ALFA-CENH3* (*GT*) plants lack haploid-inducing activity (Figure 5C). These results demonstrate that ALFA tagging of CENH3 alone is insufficient for haploid induction and instead suggest that altered gene regulation associated with the *ALFA-gCENH3* transgene in the *cenh3-1* background may be responsible for this haploidization phenotype.

To determine whether egg-cell-specific degradation of *in-locus*-tagged CENH3 in this background could lead to haploid induction, we transformed ALFA-CENH3 (GT) plants with the *EN-SPOP* construct, which was selected based on its high induction efficiency in the *ALFA-gCENH3*^*cenh3-1*^ background. Hemizygous *EN-SPOP* T1 plants in the *ALFA-CENH3* (*GT*) background were crossed with *gl1-1* pollen donors, and the progeny were screened for haploid induction. Glabrous haploids were recovered from all T1 maternal lines, with a median haploid induction frequency of 16.38% (Figure 5C). These findings conclusively demonstrate that egg-cell-specific degradation of epitope-tagged CENH3 is sufficient for haploid induction, regardless of whether the tagged CENH3 is expressed from a transgene or the tag is integrated at the endogenous *CENH3* locus.

### Engineered plant-derived EL5 effectively degrades tagged tomato CENH3

Translation of egg-cell-specific CENH3 degradation technology from *Arabidopsis* to crop species requires validation that the engineered rice-derived E3 ligase fusion protein can effectively degrade CENH3 orthologs in heterologous systems. As an initial test case, we selected tomato (*Solanum lycopersicum*) *CENH3* (*SlCENH3*) to evaluate the functionality of the engineered E3 ligase. We generated two constructs for transient expression in *Nicotiana benthamiana*: the first contained modules for constitutive expression of *EYFP-SlCENH3* and histone H2B-mCherry, both driven by the *35S* promoter; the second construct included an additional degradation module expressing EL5-VHHGFP4 under the control of the *35S* promoter (Figure 5D).

Following agroinfiltration, confocal microscopy revealed characteristic centromeric EYFP foci in all H2B-mCherry signal-positive nuclei (*n* = 38) transformed with the construct lacking the degradation module. In contrast, most H2B-mCherry-positive nuclei (96%, *n* = 50) transformed with the construct containing *EYFP-SlCENH3* and the corresponding degradation module showed a nearly complete absence of EYFP foci (Figure 5E). These results demonstrate that the plant-based engineered E3 ligase EL5 is effective at degrading tagged CENH3 from species other than *Arabidopsis*.

## Discussion

**Figure 6:**
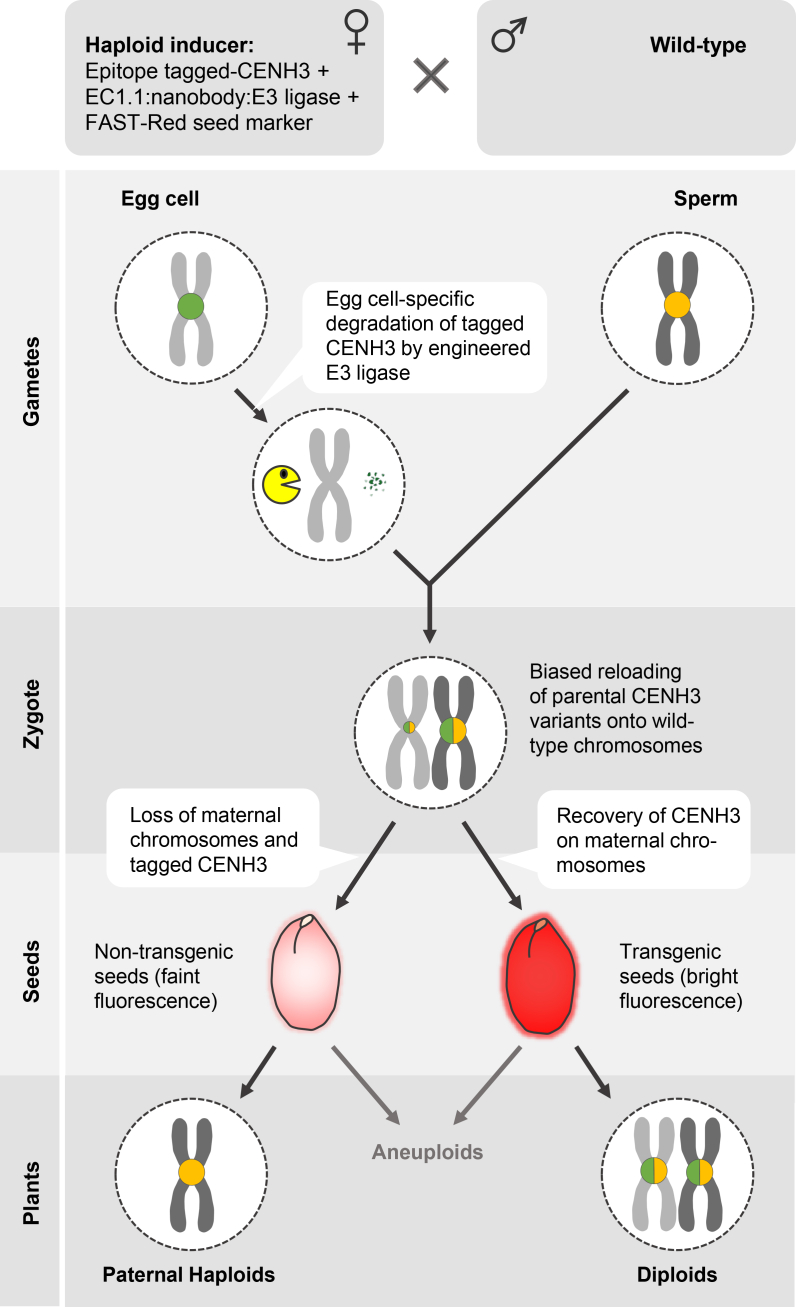
Schematic model of egg-cell-specific CENH3 depletion for paternal haploid induction. The haploid inducer line carries epitope-tagged CENH3 and is transformed with constructs containing (i) an E3 ligase module for egg-cell-specific CENH3 degradation and (ii) a FAST-Red seed marker module. Gametes: targeted degradation of tagged CENH3 generates maternal chromosomes depleted of CENH3 nucleosomes. Zygote: fertilization with a wild-type sperm produces a zygote containing parental chromosomes with pronounced CENH3 nucleosome asymmetry. Both wild-type and epitope-tagged CENH3 preferentially reload onto CENH3-enriched paternal chromosomes. Seeds: this asymmetric zygote generates two distinct seed fluorescence classes: seeds with faint fluorescence (endosperm-derived signal only) develop into non-transgenic paternal haploids and a few aneuploids, whereas seeds with bright fluorescence (combined embryo and endosperm signals) develop into transgenic diploids and aneuploids.

We developed a CENH3 degradation-based haploidization approach that produces wild-type haploids. Haploid inducers were generated by combining engineered (non-plant or plant-derived) E3 ligases with YFP- or ALFA-tagged CENH3. These haploid inducers undergo egg-cell-specific CENH3 degradation, and after outcrossing with wild-type plants, maternal chromosomes are eliminated in a subset of F1 plants, resulting in paternal, non-transgenic haploids (Figure 6). An important advantage of this approach is its scalability, allowing increased haploid induction frequency through the use of more efficient protein degradation systems.

Our findings suggest that extreme differences in CENH3 levels between parental chromosomes during early embryogenesis are critical for effective haploid induction. While gametic CENH3 dilution through heterozygous null mutations is effective in maize, its efficacy is significantly reduced in Brassicaceae and largely ineffective in wheat (Lv et al., 2020; Marimuthu et al., 2021; Wang et al., 2021, 2024). This species-specific variation in tolerance to parental CENH3 asymmetry underscores the need for strategies that actively reduce gametic CENH3 levels for broad applicability.

RNAi-mediated CENH3 downregulation has limitations, as constitutive CENH3 suppression compromises plant fitness because of its essential role in chromosome segregation (Lermontova et al., 2011b; Manape et al., 2024). Recent studies show that CENH3 RNAi driven by constitutive *RPS5A* promoters induces haploids only under elevated temperatures or enhances existing inducer frequency (Yang et al., 2025). However, RNAi driven by gametic promoters, such as *EC1.2*, fails to effectively induce haploids (Yang et al., 2025), likely due to CENH3’s intrinsic stability and low turnover in centromeric chromatin (Lermontova et al., 2011a). These findings indicate that RNAi alone is insufficient to deplete centromeric CENH3 in gametes, necessitating active protein-degradation mechanisms.

Here, we demonstrate that the ubiquitin–proteasome pathway can selectively degrade CENH3 in egg cells using engineered E3 ubiquitin ligases, generating paternal haploids upon wild-type outcrossing. Since targeted Cid depletion (the *Drosophila* CENH3 homolog) in sperm cells induces gynogenic haploids in *Drosophila* (Raychaudhuri et al., 2012), the link between CENH3 asymmetry and haploid induction appears to be evolutionarily conserved. Therefore, gametic CENH3 degradation offers a promising strategy for haploid induction across crop species.

Using *EN-SPOP* combined with ALFA-CENH3, we achieved an observed haploid induction frequency of 4.11% (median). This frequency appears to be lower than that of the GFP-tailswap modification (29%–45%) (Ravi et al., 2010). Unlike other CENH3-based modifications in *Arabidopsis*, our approach uniquely restricts haploid induction potential to gametes carrying the transgene encoding the degradation module. As the haploid induction experiments in this study used T1 plants hemizygous for CENH3 degradation constructs, efficiency should be compared based on effective rather than observed haploid induction frequency. Leveraging the FAST-Red seed marker, we show that the effective haploid induction frequency of *EN-SPOP* with ALFA-CENH3 ranges from 25% to 57%. This frequency reflects the haploid induction potential of homozygous lines carrying the *EN-SPOP* transgene cassette.

Interestingly, SPOP, which induces haploids at higher frequencies than NSlmb, also causes correspondingly higher seed lethality upon self-pollination. However, this seed lethality associated with high-efficiency haploid induction, which impedes standard segregation analysis, challenges the isolation of homozygous lines. The fluorescent seed marker system proves essential in this context, enabling the identification and selection of transgene-positive seeds among the self-pollinated progeny of hemizygous lines, thereby facilitating the maintenance and propagation of haploid-inducing genotypes.

This is the first study evaluating plant-derived engineered E3 ligases for targeted protein degradation in plants, whereas previous studies used only non-plant-organism-derived E3 ligases (Baudisch et al., 2018; Sorge et al., 2021; Huang and Rojas-Pierce, 2024). However, OsEL5 proved more efficient than *SPOP* in haploid induction only when combined with EYFP-CENH3 and showed the opposite trend with ALFA-CENH3. Previous studies demonstrated that lethality caused by a ubiquitination-deficient mutant, CENP-A K124R, could be rescued by EYFP-tagged CENP-A K124R but not by FLAG-K124R (Niikura et al., 2019), indicating that different tags can influence ubiquitination potential. Additionally, E3 ligase degradation efficiency can vary depending on the interacting domain to which it is fused, likely due to context-dependent steric hindrance (Huang and Rojas-Pierce, 2024). These findings suggest that EYFP’s larger structure compared with the ALFA tag may offer more accessible lysine residues or a better structural context for ubiquitination by the monomeric E3 ligase *EL5*. In contrast, SPOP, as a multi-subunit E3 ligase complex, may achieve an optimal geometry for effective ubiquitination of ALFA-tagged CENH3. The interplay among substrate accessibility, steric constraints, and the oligomeric state of the E3 ligase complex appears to be a critical determinant of degradation efficiency. These factors must be considered in future optimizations of protein degradation system design to identify suitable components for efficient degradation.

Applying our approach to crop plants requires two key steps: generating genotypes with a functional epitope-tagged CENH3 to facilitate the recruitment of CENH3-specific E3 ligases and transforming the tagged CENH3 line with constructs for gametic expression of engineered E3 ligases. Epitope-tagged CENH3 genotypes can be generated through two methods: complementing a *cenh3* null mutant with an epitope-tagged CENH3 transgene or using an *in-locus* protein tagging strategy to insert the tag directly at the endogenous locus. Our results demonstrate that the CENH3 degradation strategy functions effectively irrespective of the method used to generate the tagged CENH3 line. Furthermore, the *in-locus*-tagged CENH3 was helpful in bypassing potential position effects associated with transgenic CENH3.

While these steps involve transgenesis, the resulting haploids remain non-transgenic, making this strategy compatible with breeding programs. However, whether these haploids would be subject to regulatory oversight remains debatable and may depend on the country. We endeavored to assess plant E3 ligases for developing effective haploid inducers, as public sentiment may be more averse to using genetic elements from animal species than from plants (Weale, 2010).

Paternal haploids carry the cytoplasm of the female parent and are useful for cytoplasm swapping to generate male-sterile lines (Bortiri et al., 2024; Han et al., 2024). However, haploids that carry both the cytoplasm and the nuclear genome of the maternal parent are preferred in breeding programs (Wang et al., 2023). Therefore, exploring whether sperm-specific degradation of CENH3 can induce maternal haploids in the future would be of interest. However, identifying a promoter that is exclusively active in sperm nuclei that fertilize egg cells remains a challenge (Ingouff et al., 2009). Promising candidates for driving sperm-specific CENH3 degradation include promoters from genes such as *GEX2*, *GCS1*, and *MGH3* (Borg et al., 2011). The expression of these genes has been shown to be highly specific to the generative nucleus and sperm cells.

The combination of plant EL5 and EYFP-CENH3 has proven highly effective for haploid induction in *Arabidopsis*. Notably, the same combination was also successful for the degradation of transiently expressed tomato EYFP-CENH3. Thus, targeted degradation of CENH3 holds strong potential for translation to crop species.

Additionally, alternative protein degradation strategies could be explored to further improve haploid induction frequencies. Incorporating seed markers such as FAST-Red greatly facilitates rapid and efficient haploid screening. Notably, restricting genome elimination to embryos represents a major advancement, particularly for monocot species that require embryo rescue for haploid recovery. The gamete-specific CENH3 degradation strategy presented here marks a starting point for this line of research. Further optimization of components, including promoters, E3 ligases, and other factors, in alignment with synthetic biology principles, may further enhance haploid induction efficiency.

## Methods

### Plant growth conditions

*Arabidopsis* plants were cultivated under long-day conditions (16 h light/8 h dark) at 21°C in a cultivation room. Plants used for crossings were moved to a plant growth chamber (Percival) maintained at 21°C, 16 h light/8 h dark, 2 weeks before emasculation to ensure a constant temperature until complete seed set. Transgenic seeds were selected on ½ MS (Murashige and Skoog, 2006) medium supplemented with cefotaxime (100 μg/ml) and one of the following selection agents: kanamycin (50 μg/ml), hygromycin (15 μg/ml), or PPT (20 μg/ml), under long-day conditions (16 h light/8 h dark) at 21°C. *Arabidopsis* plants were stably transformed using the floral dip method as described by Clough and Bent (1998). Protoplast preparation and transformation were performed following the protocol described by Wu et al. (2009).

### Genotypic description

The *cenh3-1* mutation in the Col-0 genetic background (Ravi et al., 2010) was used as a null allele in this study. *EYFP-gCENH3* refers to a previously described transgenic CENH3 construct (Le Goff et al., 2020). *ALFA-gCENH3* denotes a transgenic construct containing genomic CENH3 sequences with a nucleotide sequence encoding the ALFA tag inserted immediately after the start codon. Expression of both transgenic CENH3 constructs is driven by the native *CENH3* promoter and terminator. The *cenh3-1* mutation was genotyped using a dCAPS marker based on *Eco*RV digestion of PCR products (Ravi et al., 2010). Primer sequences varied depending on the transgenic CENH3 construct and are provided in Supplemental Table 5.

*EYFP-gCENH3*^*cenh3-1*^ represents a homozygous *cenh3-1* null mutant complemented by the *EYFP-gCENH3* transgene. *EYFP-gCENH3*^*Wt*^ refers to wild-type (Col-0) plants expressing the *EYFP-gCENH3* transgene. FAST-Red refers to the pOLE1:OLE1-tagRFP construct encoding seed fluorescence (Shimada et al., 2010). *ALFA-gCENH3*^*cenh3-1*^ refers to the *cenh3-1* null mutant complemented by the ALFA-gCENH3 transgene. *ALFA-CENH3* (*GT*) refers to plants homozygous for an *in-locus* ALFA-tagged CENH3 allele.

### Preparation of transgenic constructs

All transgenic constructs, except the egg cell marker, were prepared using the Golden Gate-based modular cloning (MoClo) tool kit (Engler et al., 2014). The pICSL4723 vector served as the level 2 destination binary vector. Golden Gate reactions were performed following the protocol described by Grützner and Marillonnet (2020). ALFA-gCENH3 was prepared by assembling two PCR amplicons carrying *Bsa*I sites into pICH86966. PCR amplicons were generated from *Arabidopsis* genomic DNA using the primers listed in Supplemental Table 5. The *ALFA-gCENH3* plasmid and all level 0 modules generated in this study, along with their Addgene ID numbers, are listed in Supplemental Table 6. Details of the modules used to construct level 1 and level 2 plasmids can be found in Supplemental Tables 7 and 8, respectively.

For the egg cell marker *EC1.1pro:H2B-tdTomato*, entry clones containing the *EC1.1* promoter, the H2B-tdTomato coding sequence, and the rbcSE9 terminator were re-combined into pGWB501 using multi-site Gateway cloning (LR Clonase II Plus enzyme, Thermo Fisher). The destination and entry vectors used for GT were based on the previously described *pDe-EC-ttLbCas12a-i* and *pEn-RZ-LbcrRNA* plasmids (Schindele et al., 2023). The GT donor was synthesized by BioCat (Heidelberg, Germany), and flanking *Spe*I restriction sites were added for integration into the destination vector. Specification of the crRNA cassette and final assembly of the *in planta* GT expression vector were performed as previously described (Schindele et al., 2023).

### Gene targeting for *in-locus* ALFA tagging of CENH3

T1 plants transformed with the *in planta* GT expression vector were grown, and T2 seeds were harvested individually from each line. A bulk screening method (Ronspies et al., 2022) was performed on T2plants using NC368 and SY209 primers, followed by individual line screening of positive bulks with primers NC368 and NC369. Correct integration was confirmed by Sanger sequencing (Eurofins Genomics, Germany) using primer NC370. PCR and sequence analyses were conducted to identify homozygous ALFA-tag-positive lines. Primer sequences are provided in Supplemental Table 5.

### Crossing and haploid screening

Crossing and haploid screening were performed as described by Kuppu et al. (2015). The *gl1-1* mutation, which confers a recessive glabrous (trichome-less) phenotype in the *Ler* background, was used as a phenotypic marker for haploid screening. Plants for which haploid induction frequency was to be quantified were crossed with *gl1-1* plants, and seedlings were screened for glabrous plants 3 weeks after sowing. To confirm ploidy, a few randomly selected plants were then analyzed using flow cytometry.

### Transient transformation in *N. benthamiana*

Constructs were transformed into *Agrobacterium tumefaciens* strain GV3101. Agrobacteria harboring the constructs were cultured overnight at 28°C in YEB medium containing kanamycin (50 mg/l), gentamicin (30 mg/l), and rifampicin (50 mg/l) for transient transformation of *N. benthamiana* according to Phan and Conrad (2016).

### Indirect immunostaining

Indirect immunostaining was performed on fixed leaf nuclei using the protocol described by Kuo et al. (2023). Custom-made rabbit anti-*Arabidopsis* CENH3 antibodies and a recombinant ALFA nanobody (*NbALFA*) fused to a rabbit IgG Fc domain (NanoTag Biotechnologies, #N1583) were used as primary antibodies. Anti-rabbit secondary antibodies conjugated to a rhodamine fluorophore were used for fluorescent labeling.

### Microscopy

Ovules were mounted on glass slides using 50% glycerol/0.1× PBS. DAPI staining of mature pollen was performed following a method described before (Park et al., 1998). Ovules, DAPI-stained pollen, protoplasts, mature *Arabidopsis* seeds, and transiently transformed *N. benthamiana* nuclei were analyzed using a confocal laser scanning microscope (Zeiss LSM780). Images were captured as z-stacks, and maximum-intensity projections were generated in Fiji (ImageJ). Egg cells within ovules were identified using the H2B-tdTomato marker driven by the *EC1.1* promoter.

Immunostained *Arabidopsis* leaf nuclei were imaged using an Olympus BX61 epifluorescence microscope equipped with a Hamamatsu Orca ER CCD camera. Pooled seeds were imaged using a Zeiss Axio Zoom stereo microscope.

### Flow cytometry

A BD Influx cell sorter (Becton Dickinson) was used to analyze ploidy. Either young leaves or mature seeds were finely chopped with a razor blade in CyStain UV Ploidy buffer (Sysmex). The homogenate was filtered through a 50-μm nylon mesh and analyzed by flow cytometry.

### Statistical comparisons

For all statistical comparisons, the methods used for significance testing, along with *p* values and sample sizes (*n*), are provided within the figures or detailed in the figure legends.

## Funding

This research was supported by grants from DAAD (funding program ID 57507871, reference number 91767722) to S.S. and from Bayer Crop Science (USA) to A.H. S.Y. gratefully acknowledges financial support from the Study Abroad Postgraduate Education Scholarship Program (YLSY) awarded by the Republic of Türkiye Ministry of National Education.

## Acknowledgments

The MoClo toolkit was a gift from Sylvestre Marillonnet (IPB, Halle; Addgene kit #1000000044). pICSL4723 was a gift from Mark Youles (TSL, UK). We thank Luca Comai, Mohan P.A. Marimuthu (UC, Davis, USA), and Stefan Heckmann (IPK, Germany) for critical discussions of the manuscript; Armin Meister for his input on statistical analysis; and Twan Rutten (IPK, Germany) for assistance with seed microscopy. No conflict of interest is declared.

## Author contributions

S.S. performed most of the experiments with technical assistance from O.W. J.F. performed the flow cytometry experiments. J.C. contributed to microscopy experiments, and T.L. contributed to crossing and haploid screening. M.C. generated and characterized *EYFP-gCENH3* transgenic lines. A.K. contributed to aspects of experimental design. S.Y., under the supervision of N.C. and H.P., performed all experiments related to *in planta* gene targeting. J.C.L. contributed to the selection and characterization of plant-derived E3 ligases. A.H. supervised the research project. S.S., J.F., and A.H. wrote the manuscript with input from all co-authors.

## Supplemental information

Document S1. Supplemental Figures 1–7, Supplemental Tables 1–14, and Supplemental data

Document S2. Article plus supplemental information
