# Supplementary material for: Targeted CENH3 protein depletion in egg cells enables highly efficient haploid induction
Source: Plant Commun. 2026 Mar 30;7(5):101837. doi: 10.1016/j.xplc.2026.101837 (PMC13174264; doi:10.1016/j.xplc.2026.101837)
Supplement: Document S1. Supplemental Figures 1–7, Supplemental Tables 1–14, and Supplemental data [file mmc1.pdf]

Plant Communications, Volume *7*

Supplemental information

Targeted CENH3 protein depletion in egg cells enables highly efficient haploid induction

Saravanakumar Somasundaram, Seda Yaşar, Jörg Fuchs, Maria Cuacos, Julian Claassen, Oda Weiss, Andriy Kochevenko, Jonathan C. Lamb, Tengyu Li, Niklas Capdeville, Holger Puchta, and Andreas Houben

Supplemental figures:

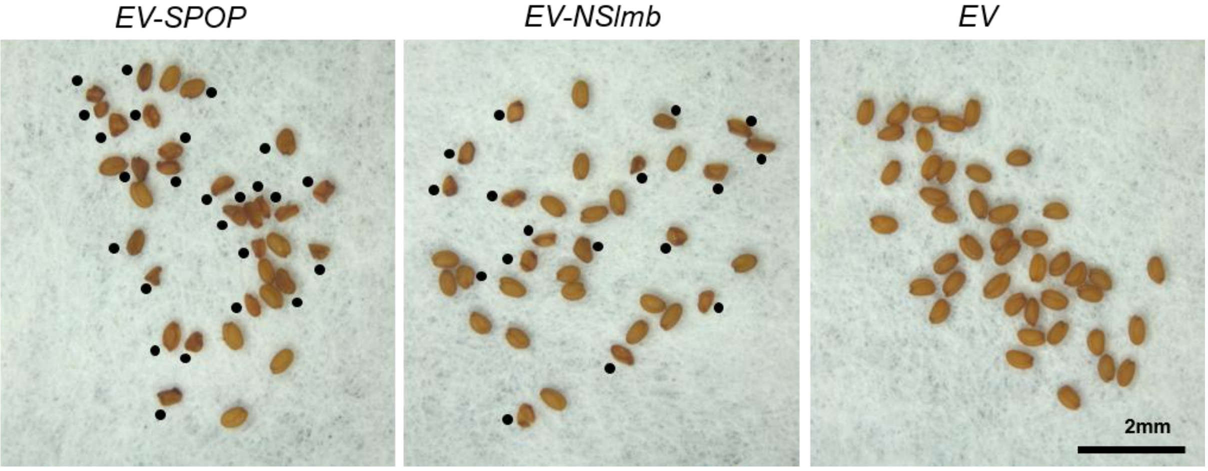

Figure S1: Egg cell-specific degradation of EYFP-CENH3 results in shriveled seeds after self-pollination. Images of seeds from a representative silique of representative *EV-SPOP, EV-NSlmb and EV T1* plants in the genetic background of *EYFP-gCENH3*$^{cenh3\text{-}1}$. Black dots indicate the shriveled seeds.

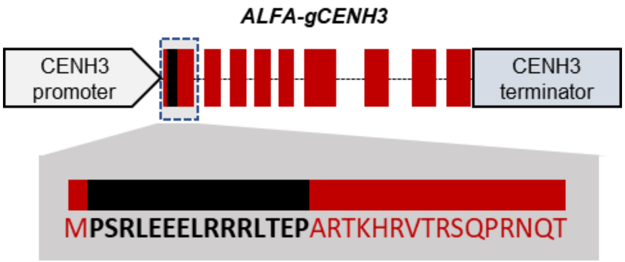

Figure S2: Schematic representation of the *ALFA-gCENH3* construct showing the site of the ALFA-tag insertion. The first exon is zoomed in, and the amino acids highlighted in black corresponds to the ALFA-tag.

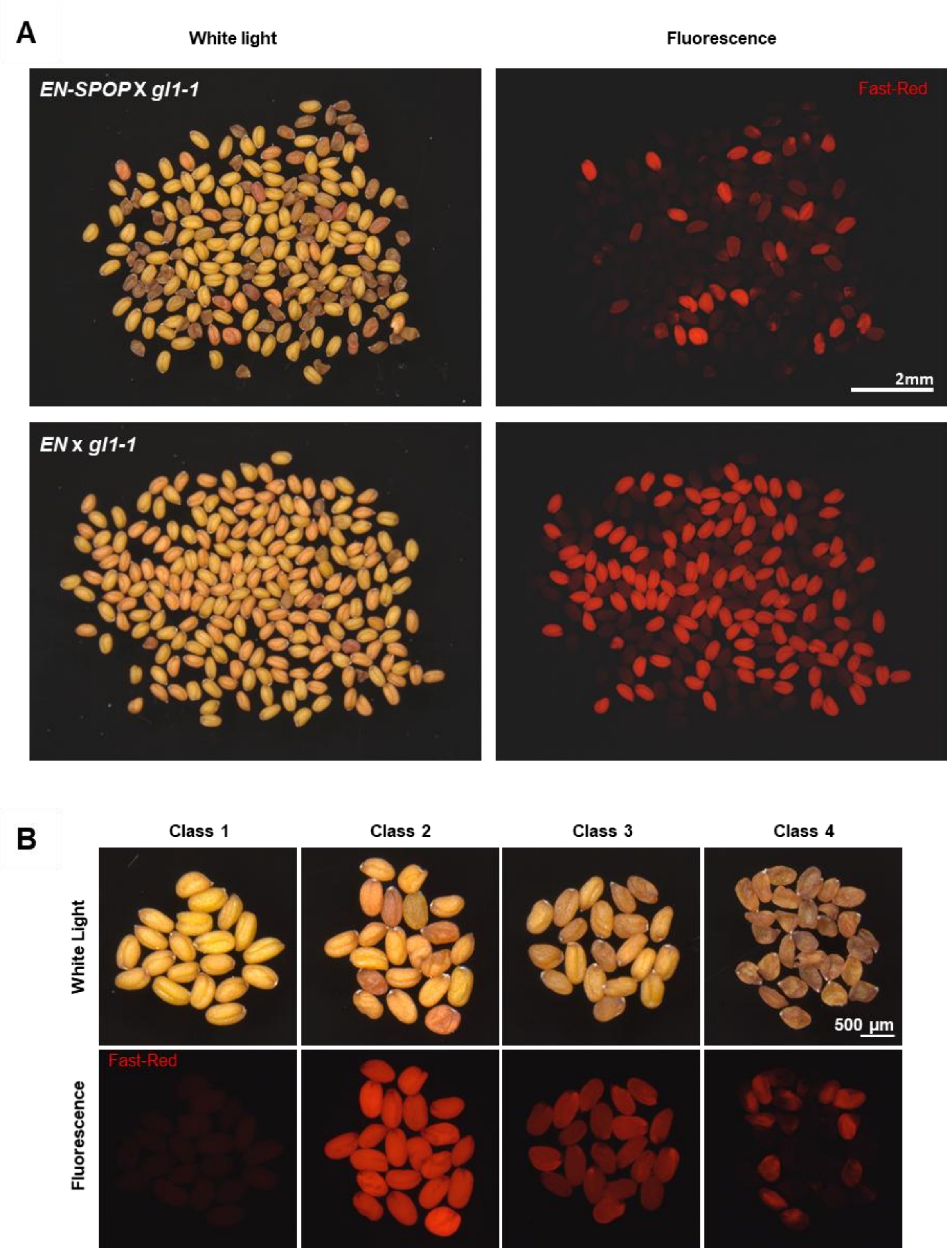

Figure S3: Comparison of F1 seed phenotypes from *ALFA-gCENH3*$^{cenh3\text{-}1}$ mothers with and without engineered E3 ligases. A) Seeds were obtained from crosses between *ALFA-gCENH3*$^{cenh3\text{-}1}$ mothers carrying either *EN-SPOP* (with engineered E3 ligases) and *EN* (control without E3 ligases) transgene and *gl1-1* fathers. Crosses with *EN* mothers produced

predominantly viable F1 seeds with a high proportion exhibiting fluorescence. In contrast, crosses involving *EN-SPOP* mothers resulted in increased seed lethality and a substantially reduced proportion of fluorescent seeds. Fluorescent seeds could be further classified based on fluorescence intensity differences. **B)** Different seed classes observed among the F1 seeds from EN-SPOP T1 females.

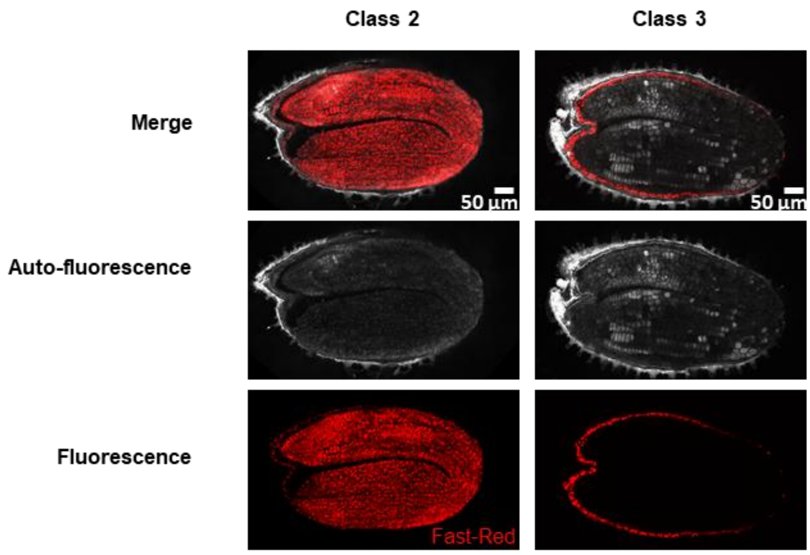

Figure S4: Differential fluorescence distribution in Class 2 and Class 3 seeds. In Class 2 seeds, fluorescence is detected in both the embryo and endosperm. In contrast, Class 3 seeds, which primarily give rise to haploids, exhibit fluorescence only in the endosperm, with no signal observed in the embryo.

A

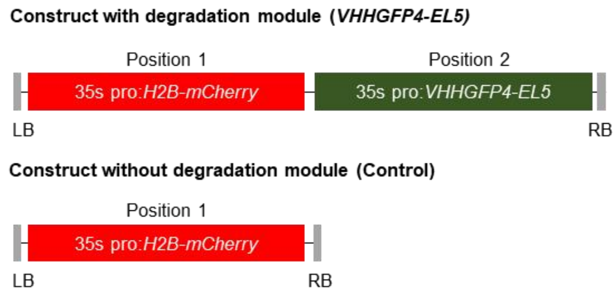

B

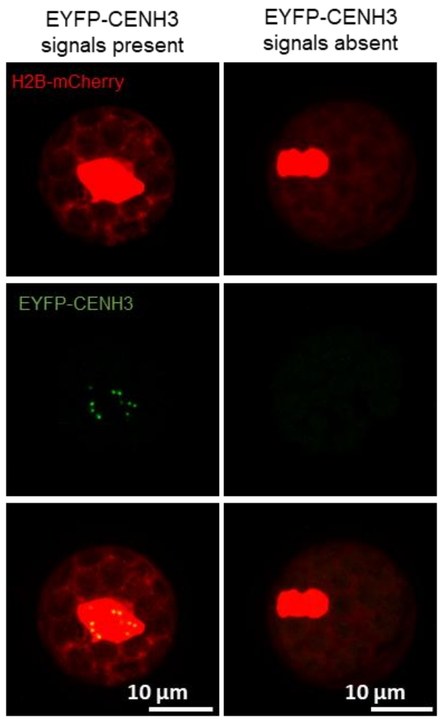

C

Construct	n	Protoplasts with EYFP-CENH3 signal (%)	Protoplasts without EYFP-CENH3 signal (%)
VHHGFP4-EL5	53	3.77	96.23
Control	56	82.14	17.86

Figure S5: Assessment of the functionality of the E3 ligase, EL5 for CENH3 degradation: A) Schematic representation of transgene cassettes with and without the degradation module (VHHGFP4-EL5) used for protoplast transformation. Protoplasts were derived from *EYFP-gCENH3*$^{cenh3\text{-}1}$ plants. **B)** Representative protoplasts showing nuclei labelled with histone H2B-mCherry (red) and displaying either presence or absence of EYFP-CENH3. **C).** Proportion of protoplasts with and without EYFP-CENH3 signals following transformation with the constructs shown in panel (A). 'n' indicates the total number of transformed protoplasts analysed for each construct.

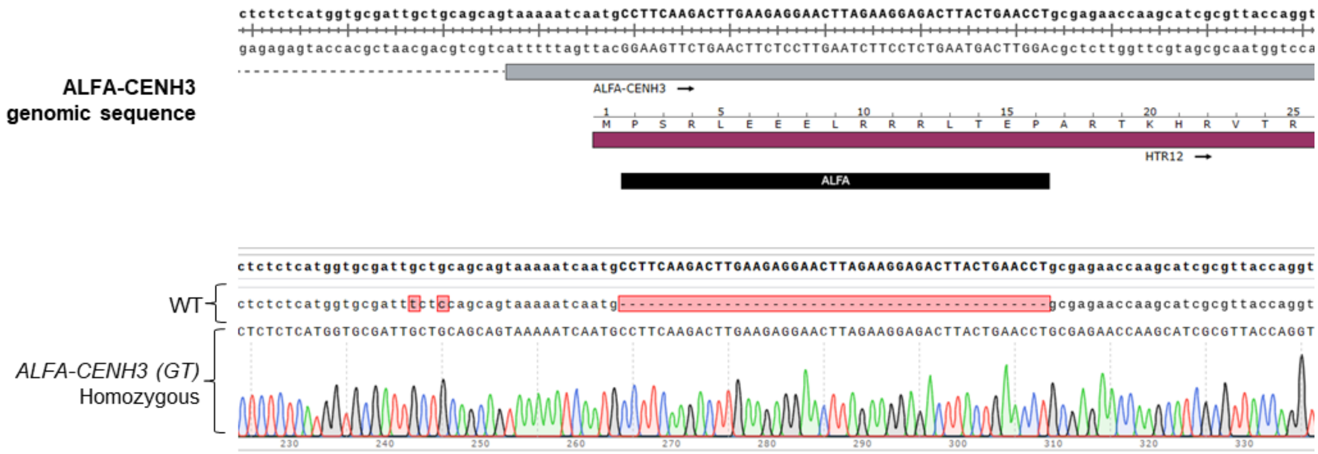

Figure S6: Sanger sequencing chromatogram of a representative *ALFA-CENH3 (GT)* homozygous plant. The sequence is compared with the WT sequence, and the sites where silent mutations were introduced are highlighted.

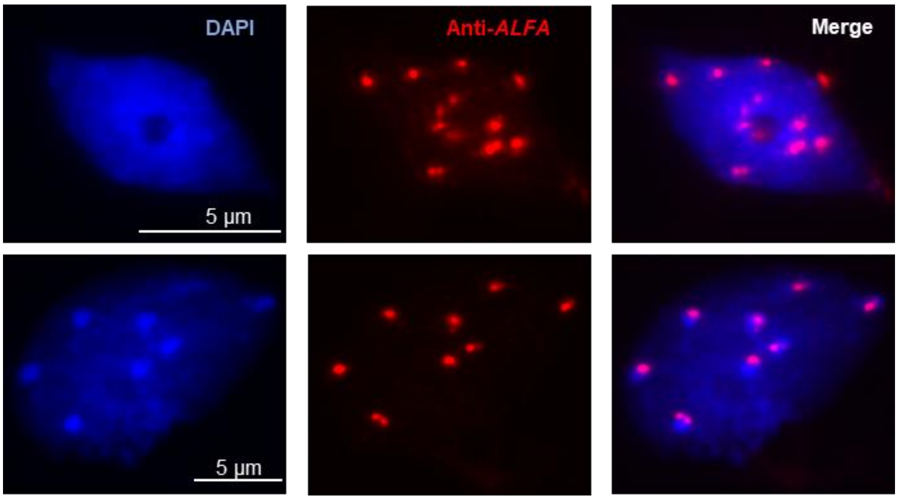

Figure S7: Immunolabeling of leaf nuclei from *ALFA-CENH3 (GT)* homozygous plant with anti-ALFA antibody.

Supplemental Tables

Table S1: ***EYFP-gCENH3$^{cenh3\text{-}1}$*** **results in variable frequencies of haploids on outcrossing.** Haploids are determined based on glabrous seedlings among the progenies observed from crosses with *gl1-1* fathers.

Replicates	EYFP-gCENH3$^{cenh3\text{-}1}$ @			EYFP-gCENH3$^{cenh3\text{-}1}$ #		
	Total	Haploids	(%)	Total	Haploids	(%)
1	48	6	12.50	27	4	14.81
2	271	15	5.54	17	2	11.76
3	62	12	19.35	46	6	13.04
4	59	12	20.34	26	3	11.54
5	68	8	11.76	41	5	12.20
6	184	15	8.15	89	6	6.74
7	29	4	13.79	32	4	12.50
8	41	4	9.76	16	1	6.25
9	19	2	10.53	74	6	8.11
10	32	4	12.50	31	3	9.68

@ - line used in the previous study (Demidov et al. 2022). # - line from an independent complementation experiment.

Table S2: Proportion of F1 seed classes from *ALFA-gCENH3*$^{cenh3\text{-}1}$ mother plants with and without engineered E3 ligases, and their corresponding germinated and glabrous seedlings. F1 seeds from three independent T1 mother plants were analyzed for each construct: *EN-SPOP* (with engineered E3 ligases) and *EN* (control without E3 ligases). Seeds were classified based on fluorescence intensity and morphological characteristics as described in Fig. 5. Numbers in parentheses indicate the proportion of seeds in each category relative to total seeds analyzed.

Cross	T1 replicate	Total number of seeds analysed	No. of seeds (%)				Germinated seedlings				Glabrous seedlings			
			Class 1	Class 2	Class 3	Class 4	Class 1	Class 2	Class 3	Class 4	Class 1	Class 2	Class 3	Class 4
EN-SPOP x *gl1-1*	1	604	275 (45.5 %)	75 (12.4 %)	44 (7.3 %)	210 (34.8 %)	251	13	6	5	0	0	6	0
	2	1206	468 (38.8 %)	78 (6.5 %)	76 (6.3 %)	584 (48.4 %)	420	10	17	1	0	0	17	0
	3	1032	465 (45.1 %)	108 (10.5 %)	68 (6.6 %)	391 (37.9 %)	429	13	15	8	0	0	13	0
EN x *gl1-1*	1	641	156 (24.3 %)	460 (71.8 %)	0	25 (3.9 %)	141	395	0	2	0	0	0	0
	2	799	355 (44.4 %)	416 (52.1 %)	0	28 (3.5 %)	326	391	0	3	0	0	0	0
	3	679	296 (43.6 %)	314 (46.2 %)	0	69 (10.2 %)	258	272	0	0	0	0	0	0

Table S3: Ploidy analysis of different F1 seed classes from *EN-SPOP* plants. Flow cytometry analysis was performed on 'n' seeds per class from *EN-SPOP* T1 mother plants. Diploid Col-0 seeds served as internal reference for classes 1-3. Class 4 seeds were measured without an internal reference due to low number of isolatable nuclei in this seed class.

Cross	Seed category	n	Haploid (%)	Diploid (%)	Aneuploid (%)	Mixoploid (%)
EN-SPOP x *gl1-1*	Class 1	20	0	100	0	0
	Class 2	40	0	72.5	25	2.5
	Class 3	40	72.5	15	7.5	5
	Class 4	20	0	0	0	0

Table S4: Accuracy of FAST-Red seed marker and glabrous phenotype for haploid progeny identification. The accuracy of FAST-Red seed marker was calculated as the proportion of flow cytometry-confirmed haploids among germinated seedlings derived from seed class 3 exhibiting faint FAST-Red fluorescence. Accuracy of glabrous phenotype was calculated as the proportion of flow cytometry-confirmed haploids among glarous seedlings.

Cross	T1 replicate	Seedlings from class 3 seeds	Total glabrous seedlings	Haploids based on flow cytometry	Accuracy of glabrous phenotype (%)	Accuracy of class 3 seed phenotype (%)
EN-SPOP x *gl1-1*	1	6	6	6	100.00	100.00
	2	17	17	16	94.12	94.12
	3	15	13	13	100.00	86.67
	**Total**	**38**	**36**	**35**	**97.22**	**92.11**

Table S5: Primer sequences used in this study.

Purpose	Primer name	Sequence
EcoRV dCAPS for genotyping *cenh3-1* (in case of *EYFP-gCENH3* complementation)	CenH3-dCAPS-F	GGTGCGATTTCTCCAGCAGTAAAAATC
	CenH3-dCAPS-R	CTGAGAAGATGAAGCACCGGCGATAT
EcoRV dCAPS for genotyping cenh3-1 (in case of *ALFA-gCENH3* complementation)	AtCENH3-Geno-F2	CTCCAGCAGTAAAAATCAATGGATAGA
	CenH3-dCAPS-R	CTGAGAAGATGAAGCACCGGCGATAT
Primers used for generating PCR fragments to assemble ALFA-gCENH3	CENH3-Bsa1-F1	TTGGTCTCATCCCGTACGGACGCATCATC AACATCTG
	CENH3-Bsa1-R1	TTGGTCTCATTCCTCTTCCAGCCTGGACG GCATTGATTTTTACTGCTGGAGAAATC
	CENH3-Bsa1-F2	TTGGTCTCAGGAACTCAGACGTAGGCTCA CGGAACCTGCGAGAACCAAGCATCGCG
	CENH3-Bsa1-R2	TTGGTCTCACAGATTTCGTTTAAGGACCA ATGAG
Gene-targeting for *in-locus* ALFA tagging of CENH3	NC368	CTATCCTCGGTAATGACCAG
	NC369	CCTGTCTGGATCTCTTAGCC
	NC370	CTAACAATCGAGTCGAACCC
	SY209	CCTCTTCAAGTCTTGAAGG

Table S6: Level-0 modules used in this study and their Addgene ID number.

Module Name	Destination	Addgene ID
NOS-PPT	pICH41331	232550
CAMV35S-promoter	pICH41295	232551
EC1.1-promoter	pICH41295	232552
A9-promoter	pICH41295	232553
HSP-terminator	pAGM9121	232554
RbcSE9-tererminator	pAGM9121	232555
Barnase	pAGM1287	232556
H2B	pICH41258	232557
mCherry	pAGM1299	232558
VHHGFP4	pAGM1287	232559
VHHGFP4-SPOP	pAGM1287	232560
Nslmb-VHHGFP4	pAGM1287	232561
NbALFA-N	pICH41258	232562
NbALFA-C	pAGM1299	232563
NbALFA	pAGM1287	232564
Nslmb	pICH41258	232565
SPOP	pAGM1299	232566
EYFP	pICH41258	232567
CENH3-cds	pAGM9121	232568
EL5	pAGM1299	232569
VHHGFP4-N	pICH41258	232571
VHHGFP4-C	pAGM1299	232572
ALFA-gCENH3	pICH86966	232575

Table S7: Level-1 modules used in this study: List of level-0 modules and destination plasmid used for the construction of level-1 plasmids.

Construct	Destination	Level-0 modules			
		1	2	3	4
PPT	pICH47802	NOS-PPT	-	-	-
Kan	pICH47802	NOS-Kan			
EVS	pICH47811	EC1.1-pro	VHHGFP4-SPOP	RbcSE9-terminator	-
ENV	pICH47811	EC1.1-promoter	Nslmb-VHHGFP4	RbcSE9-terminator	-
EV	pICH47811	EC1.1-promoter	VHHGFP4	RbcSE9-terminator	-
ENS	pICH47811	EC1.1-promoter	NbALFA-N	SPOP	RbcSE9-terminator
ENN	pICH47811	EC1.1-promoter	Nslmb	NbALFA-C	RbcSE9-terminator
EN	pICH47811	EC1.1-promoter	NbALFA	RbcSE9-terminator	-
ENE	pICH47811	EC1.1-promoter	NbALFA-N	EL5	RbcSE9-terminator
EVE	pICH47811	EC1.1-promoter	VHHGFP4-N	EL5	RbcSE9-terminator
FAST	pICH47822	pICSL70008 (Addgene #50336)	-	-	-
Barnase	pICH47822/ pICH47831	A9-promoter	Barnase	HSP-terminator	
H2B-mCherry	pICH47732	CAMV35S-promoter	H2B	mCherry	HSP-terminator
Degradation module (VHHGFP4-EL5)	pICH47742	CAMV35S-promoter	VHHGFP4-N	EL5	RbcSE9-terminator
EYFP-*Sl*CENH3	pICH47751	CAMV35S-promoter	EYFP	*Sl*CENH3 (Synthetic fragment)	RbcSE9-terminator

Table S8: Level-2 modules used in this study: List of level-1 modules used for the construction of level-2 plasmids.

Construct	Destination	Position 1	Position 2	Position 3	Position 4
EV-SPOP	pICSL4723	PPT	EVS	-	-
EV-NSlmb	pICSL4723	PPT	ENV	-	-
EV	pICSL4723	PPT	EV	-	-
EN-SPOP	pICSL4723	PPT	ENS	FAST	Barnase
EN-SPOP (for transformation of *ALFA-CENH3* (GT) plants)	pICSL4723	Kan	ENS	FAST	Barnase
EN-NSlmb	pICSL4723	PPT	ENN	FAST	Barnase
EN	pICSL4723	PPT	EN	FAST	Barnase
Control (only Barnase) for *ALFA-gCENH3*	pICSL4723	PPT	-	FAST	Barnase
EN-EL5	pICSL4723	PPT	ENE	FAST	Barnase
EV-EL5	pICSL4723	PPT	EVE	Barnase	
Control (only Barnase) for *EYFP-gCENH3*	pICSL4723	PPT	-	Barnase	
Construct with degradation Module (VHHGFP4-EL5)	pICSL4723	H2B-mCherry	VHHGFP4-EL5		
Control Construct without degradation Module	pICSL4723	H2B-mCherry	-		
Construct with degradation Module (VHHGFP4-EL5) for tomato CENH3 experiment	pICSL4723	H2B-mCherry	VHHGFP4-EL5	EYFP-*Sl*CENH3	
Control Construct without degradation Module for tomato CENH3 experiment	pICSL4723	H2B-mCherry	-	EYFP-*Sl*CENH3	

Table S9: Haploid induction frequency of individual crosses presented in Figure 2D.

Construct	Replicate	No. of progenies screened	No. of Haploid (glabrous) plants	Haploid frequency (%)	Median (%)
EV-SPOP	1	87	26	29.89	25.17
	2	94	20	21.28	
	3	19	11	57.89	
	4	24	4	16.67	
	5	142	37	26.06	
	6	138	24	17.39	
	7	131	49	37.40	
	8	173	42	24.28	
	9	79	23	29.11	
	10	60	17	28.33	
	11	92	24	26.09	
	12	38	8	21.05	
	13	153	29	18.95	
	14	79	18	22.78	
EV-NSlmb	1	126	28	22.22	22.315
	2	79	28	35.44	
	3	241	54	22.41	
	4	323	58	17.96	
	5	186	74	39.78	
	6	514	89	17.32	
	7	65	10	15.38	
	8	116	40	34.48	
	9	245	41	16.73	
	10	305	41	13.44	
	11	100	27	27.00	
	12	117	32	27.35	
	13	171	44	25.73	
	14	333	52	15.62	
	15	83	14	16.87	
	16	247	81	32.79	
EV	1	357	60	16.81	23.08
	2	191	34	17.80	
	3	134	30	22.39	
	4	276	69	25.00	
	5	107	25	23.36	
	6	149	39	26.17	
	7	118	32	27.12	
	8	79	26	32.91	
	9	337	64	18.99	
	10	195	45	23.08	
	11	69	17	24.64	
	12	181	41	22.65	
	13	24	5	20.83	
Control	1	46	5	10.87	17.105
	2	38	5	13.16	
	3	97	11	11.34	
	4	76	18	23.68	
	5	109	23	21.10	
	6	39	9	23.08	

	7	118	7	5.93	
	8	169	41	24.26	
	9	40	5	12.50	
	10	133	28	21.05	

Table S10: Haploid induction frequency of individual crosses presented in Figure 3B.

T1_Family	Replicate	No. of progenies screened	No. of Haploid (glabrous) plants	Haploid frequency (%)
34-1	1	298	13	4.36
	2	237	5	2.11
	3	192	10	5.21
	4	218	3	1.38
	5	220	5	2.27
	6	200	10	5.00
	7	143	3	2.10
	8	200	11	5.50
	9	244	9	3.69
	10	237	5	2.11
34-2	1	56	3	5.36
	2	46	2	4.35
	3	66	5	7.58
	4	267	0	0.00
	5	20	3	15.00
	6	454	0	0.00
	7	87	5	5.75
	8	434	0	0.00
	9	54	3	5.56
	10	254	0	0.00
34-8	1	44	3	6.82
	2	128	5	3.91
	3	140	4	2.86
	4	50	2	4.00
	5	36	2	5.56
	6	55	3	5.45
	7	90	1	1.11
	8	183	3	1.64
	9	87	2	2.30
	10	56	2	3.57
34-11	1	88	13	14.77
	2	82	4	4.88
	3	108	13	12.04
	4	90	5	5.56
	5	89	10	11.24
	6	66	10	15.15
	7	106	8	7.55
	8	89	8	8.99
	9	107	6	5.61
	10	111	12	10.81
34-15	1	108	2	1.85
	2	219	11	5.02
	3	124	5	4.03
	4	443	5	1.13
	5	230	3	1.30
	6	421	1	0.24
	7	302	5	1.66
	8	303	16	5.28
	9	435	1	0.23

	10	431	1	0.23
34-20	1	124	4	3.23
	2	80	4	5.00
	3	136	11	8.09
	4	92	3	3.26
	5	153	8	5.23
	6	122	6	4.92
	7	98	5	5.10
	8	54	3	5.56
	9	91	6	6.59
	10	54	6	11.11

Table S11: Haploid induction frequency of individual crosses presented in Figure 3D.

Construct	Replicate	No. of progenies screened	No. of Haploid (glabrous) plants	Haploid frequency (%)	Median (%)
EN-SPOP	1	342	10	2.92	4.11
	2	324	18	5.56	
	3	76	7	9.21	
	4	73	3	4.11	
	5	307	10	3.26	
	6	280	8	2.86	
	7	93	12	12.90	
	8	350	13	3.71	
	9	230	3	1.30	
	10	169	8	4.73	
	11	167	10	5.99	
EN-NSlmb	1	172	7	4.07	1.31
	2	342	3	0.88	
	3	322	2	0.62	
	4	185	1	0.54	
	5	290	3	1.03	
	6	287	8	2.79	
	7	228	4	1.75	
	8	301	0	0.00	
	9	252	4	1.59	
	10	71	2	2.82	
EN	1	526	0	0.00	0.00
	2	260	0	0.00	
	3	384	0	0.00	
	4	312	2	0.64	
	5	428	0	0.00	
	6	249	0	0.00	
	7	248	0	0.00	
	8	388	0	0.00	
	9	380	0	0.00	
	10	284	0	0.00	
Control	1	530	0	0.00	0.00
	2	245	0	0.00	
	3	421	0	0.00	
	4	398	0	0.00	
	5	487	0	0.00	
	6	268	0	0.00	
	7	354	0	0.00	
	8	289	0	0.00	
	9	189	0	0.00	
	10	464	0	0.00	

Table S12: Haploid induction frequency of individual crosses presented in Figure 4C.

Construct	Replicate	No. of progenies screened	No. of Haploid (glabrous) plants	Haploid frequency (%)	Median (%)
Control	1	384	0	0.00	0.00
	2	591	0	0.00	
	3	458	0	0.00	
	4	393	0	0.00	
	5	534	0	0.00	
	6	351	0	0.00	
	7	298	0	0.00	
	8	421	0	0.00	
	9	398	0	0.00	
	10	448	0	0.00	
EN-EL5	1	281	5	1.78	1.76
	2	287	5	1.74	
	3	243	7	2.88	
	4	215	2	0.93	
	5	185	7	3.78	
	6	213	2	0.94	
	7	239	20	8.37	
	8	155	4	2.58	
	9	227	3	1.32	
	10	181	2	1.10	

Table S13: Haploid induction frequency of individual crosses presented in Figure 4D.

Construct	Replicate	No. of progenies screened	No. of Haploid (glaborous) plants	Haploid frequency (%)	Median (%)
Control	1	28	4	14.29	17.50
	2	30	7	23.33	
	3	38	11	28.95	
	4	26	5	19.23	
	5	28	3	10.71	
	6	34	5	14.71	
	7	36	4	11.11	
	8	31	6	19.35	
	9	40	7	17.50	
	10	35	4	11.43	
	11	33	6	18.18	
	12	16	2	12.50	
	13	23	5	21.74	
	14	88	9	10.23	
	15	30	6	20.00	
EV-EL5	1	25	7	28.00	51.39
	2	7	4	57.14	
	3	6	3	50.00	
	4	6	4	66.67	
	5	9	3	33.33	
	6	20	11	55.00	
	7	10	4	40.00	
	8	20	7	35.00	
	9	36	19	52.78	
	10	24	16	66.67	
	11	12	6	50.00	
	12	18	6	33.33	
	13	27	16	59.26	
	14	16	9	56.25	
	15	16	5	31.25	
	16	33	21	63.64	
	17	34	21	61.76	
	18	25	9	36.00	
	19	25	18	72.00	
	20	28	8	28.57	
	21	36	15	41.67	
	22	21	13	61.90	
	23	44	26	59.09	
	24	23	11	47.83	

Table S14: Haploid induction frequency of individual crosses presented in Figure 5C.

Construct	Replicate	No. of progenies screened	No. of Haploid (glaborous) plants	Haploid frequency (%)	Median (%)
Control	1	136	0	0.00	0.00
	2	154	0	0.00	
	3	195	0	0.00	
	4	210	0	0.00	
	5	145	0	0.00	
	6	223	0	0.00	
	7	123	0	0.00	
	8	129	0	0.00	
	9	232	0	0.00	
	10	113	0	0.00	
EN-SPOP	1	277	32	11.55	16.38
	2	223	43	19.28	
	3	110	17	15.45	
	4	124	25	20.16	
	5	177	31	17.51	
	6	232	38	16.38	
	7	230	45	19.57	
	8	220	23	10.45	
	9	192	26	13.54	
	10	154	28	18.18	
	11	124	13	10.48	

Supplemental Data

Data S1: Sequence information relevant to in-locus ALFA tagging of CENH3

>CENH3 Cas12a cleavage site

TCCAGCAGTAAAAATCAATGGCGA

>*At*CENH3 GT donor – pUC57 (SpeI flanked)

The sequence organization is as follows: SpeI site – CRISPR target/PAM – 5′ homology arm including two silent point mutations (in red) – ALFA-tag CDS – 3′ homology arm – CRISPR target/PAM – SpeI site.

actagttttctccagcagtaaaaatcaatggcgaAACCTATGATTGGATGCTGAGAACTTGTAAAGAATCTGAGGC
AGAAAGTTGAAAAACTGTGTCAATTTCATTAACTTGAAAAGATGAGCATAAATTGGGAGAGA
GAGAGAGAGACAAAGATTTTGAATTGAGGTTTAACGGTAAAACACACAAAACCTATTCCCCT
CTGTTTCCAATTTTCATCTAAACAAACAGGTACATATTTGAATGTAATATTGTATACAGACCAGG
GGTAAAACAGGAACTAAAGAAGGCTAACAATCGAGTCGAACCCTCTATGTGAAGCCACAGGT
TTAGTGCAAATTGTAATAAGTTGTTCAGAGAGACTCTTGACTGAAACAAATTGTGAAGCAGAT
TCGATTTTAAAATCAAAATTTGAGTGTCGAGCGGGAAAGTAAAAGTTCCGCTCCAATCTTCTA
ATCTTTTCGTATCTAGCGGGAAATTTCTCAGCAGGTGACTTTCATAATCGCAGTTTTCGTCGATT
CTCTTTTCCGATTTTACGATTCCTCTCTCTCTCTCATGGTGCGATT**G**CT**G**CAGCAGTAAAAATC
AATGCCTTCAAGACTTGAAGAGGAACTTAGAAGGAGACTTACTGAACCTGCGAGAACCAAG
CATCGCGTTACCAGGTCACAACCTCGGAATCAAACTGGTATCTTAAATCTGCTTTCTCTTTCAA
TTTTTACTTCTGATTTTACCCAGAATTTTAGGTTTTTTATTTCGATTTTGTTAACCCTAGATTTCG
AATCTGAAATTTGTAGATGCCGCCGGTGCTTCATCTTCTCAGGCGGCAGGTCCAACTACGGTA
CGGCATCTTTTTCCGTCTTAGGGTTTCCAATGTTTCTTCCTTTTATCGTTATGATCAAATTTGTTT
ATCTATCGAAATTGAAGACCCCGACAAGGAGAGGCGGTGAAGGTGGAGATAATACTCAACAA
AGTGAGTTTTTTATATTTGAAGTCTTTTTTTTCCCTCTTTTCATCTCTTTTGTTTGTGAAGTTATT
CTTTTGTAACATCTGCAGCAAATCCTACAACTTCACCAGCTACTGGTACAAGGGTAAGATTTTT
GTGACCATTGCTTATGAACTGCTTCAACTTTGATTTCGTTATTAAGCTGACAAAATTCTCGTTTT
GGTTtcgccattgatttttactgctggagaaaactagt
