# Supplementary material for: Targeted CENH3 protein depletion in egg cells enables highly efficient haploid induction
Source: Plant Commun. 2026 Mar 30;7(5):101837. doi: 10.1016/j.xplc.2026.101837 (PMC13174264; doi:10.1016/j.xplc.2026.101837)
Supplement: Document S2. Article plus supplemental information [file mmc2.pdf]

Plant Communications
Resource article

CellPress
Partner Journal

Targeted CENH3 protein depletion in egg cells enables highly efficient haploid induction

Saravanakumar Somasundaram[1,*], Seda Yaşar[2], Jörg Fuchs[1], Maria Cuacos[1], Julian Claassen[1], Oda Weiss[1], Andriy Kochevenko[1], Jonathan C. Lamb[3], Tengyu Li[1], Niklas Capdeville[2], Holger Puchta[2] and Andreas Houben[1,4,*]

[1]Leibniz Institute of Plant Genetics and Crop Plant Research (IPK) Gatersleben, Corrensstrasse 3, 06466 Seeland, Germany

[2]Joseph Gottlieb Kölreuter Institute of Plant Sciences (JKIP) – Molecular Biology, Karlsruhe Institute of Technology (KIT), 76131 Karlsruhe, Germany

[3]Bayer U.S. – Crop Science, 700 Chesterfield Parkway West, Chesterfield, MO 63017, USA

[4]Martin Luther University Halle Wittenberg, Institute of Agricultural & Nutritional Sciences, 06120 Halle (Saale), Germany

***Correspondence: Saravanakumar Somasundaram (somasundaram@ipk-gatersleben.de), Andreas Houben (houben@ipk-gatersleben.de)**

https://doi.org/10.1016/j.xplc.2026.101837

ABSTRACT

Doubled haploid technology is a transformative tool for accelerating plant breeding by enabling the rapid development of homozygous lines. While manipulation of the centromere-specific histone H3 variant CENH3 has been shown to induce haploids in *Arabidopsis* and selected crop species, a broadly applicable approach remains elusive. The prevailing hypothesis is that CENH3 asymmetry between parental genomes during early embryonic development leads to the selective elimination of parental chromosomes with reduced CENH3 and consequently weaker centromeres. We experimentally validate this hypothesis by depleting EYFP- or ALFA-tagged CENH3 using ubiquitin-mediated proteasomal degradation specifically in the egg cell prior to fertilization with wild-type pollen. This approach consistently generated paternal wild-type haploids, with induction frequencies of up to 57% among progeny derived from egg cells containing constructs for CENH3 depletion. We further enhanced the system by incorporating a plant-derived E3 ubiquitin ligase for efficient CENH3 degradation and a fluorescent seed marker for rapid haploid identification. This approach also proved effective in *in-locus* ALFA-tagged CENH3 lines generated by gene targeting. Furthermore, the plant-derived E3 ubiquitin ligase successfully degraded CENH3 from tomato. Thus, this gametic CENH3-depletion system establishes a rational, modular framework for engineering haploid inducers and provides a potentially universal platform for haploid induction across diverse crop species.

Key words: ALFA-tag, centromere, haploid induction, uniparental genome elimination, targeted protein degradation, nanobody, *in locus* protein tagging, gene targeting

Somasundaram S., Yaşar S., Fuchs J., Cuacos M., Claassen J., Weiss O., Kochevenko A., Lamb J.C., Li T., Capdeville N., Puchta H., and Houben A. (2026). Targeted CENH3 protein depletion in egg cells enables highly efficient haploid induction. Plant Comm. **7**, 101837.

INTRODUCTION

Haploid inducers are specialized genotypes that facilitate haploid production *in planta* through intra-specific hybridization, enabling haploids to be directly obtained from seeds (Widiez, 2021). This approach can be broadly classified into (i) centromere-based and (ii) non-centromere-based methods. Centromere-based methods involve the manipulation of the centromere-specific histone H3 variant (CENH3) or kinetochore proteins, such as KNL2, to alter chromosome segregation (Ravi and Chan, 2010; Lv et al., 2020; Wang et al., 2021; Ahmadli et al., 2023). In contrast, non-centromere-based methods rely on mutations in genes such as *MTL*, *DMP*, and *Kokopelli*, which disrupt various reproductive processes to induce haploids (Kelliher et al., 2017; Zhong et al., 2019; Jacquier et al., 2023). Generally, centromere-based methods can induce both paternal and maternal haploids in monocots and dicots. In contrast, non-centromere-based methods induce haploids containing maternal chromosomes and are often specific to either monocots or dicots (Quiroz et al., 2024). Among these systems, CENH3 manipulation has proven the most efficient *in vivo* method for haploid induction, especially in *Arabidopsis thaliana*, as reviewed by Quiroz et al. (2024).

Plant Communications 7, 101837, May 11 2026 © 2026 The Author(s). Published by Elsevier Inc. on behalf of CAS Center for Excellence in Molecular Plant Sciences, Chinese Academy of Sciences, and Chinese Society for Plant Biology. This is an open access article under the CC BY license (http://creativecommons.org/licenses/by/4.0/).

In *Arabidopsis*, homozygous *cenh3* null mutants expressing a chimeric "GFP-tailswap" protein induced up to 45% paternal haploids and 5% maternal haploids when outcrossed with wild-type plants (Ravi and Chan, 2010). Other CENH3 manipulations, such as point mutations, heterologous CENH3 complementation, and inactivation of kinetochore proteins such as KNL2, have also enabled haploid induction (Karimi-Ashtiyani et al., 2015; Kuppu et al., 2015, 2020; Maheshwari et al., 2015; Ahmadli et al., 2023). Environmental factors, particularly temperature, have been shown to further modulate haploid induction frequency (Ahmadli et al., 2023; Jin et al., 2023; Wang et al., 2023).

Despite success in *Arabidopsis*, translating centromere-based haploid induction to crop species has proven challenging. In maize, plants heterozygous for a *cenh3*-null mutation produced 5% paternal haploids upon outcrossing (Wang et al., 2021). Under similar conditions, no haploids were induced in wheat, and an extremely low frequency (0.4%) was observed in *Arabidopsis* (Lv et al., 2020; Marimuthu et al., 2021). Alternatively, a specific type of mutation, namely a restored frameshift allele, was effective for haploid induction at frequencies of up to 8% in wheat (Lv et al., 2020). A similar kind of allele resulted in haploid induction in broccoli (Han et al., 2024). RNAi-mediated downregulation of CENH3 triggered haploid production in onion and cotton but not in maize or *Arabidopsis* (Kelliher et al., 2016; Gao et al., 2020; Ahmadli et al., 2023; Manape et al., 2024). Thus, although CENH3 manipulation enables haploid induction across various species, no single strategy can be universally applied across all plant species.

Our earlier work demonstrated targeted EYFP-CENH3 degradation using engineered E3 ligases encoded by the paternal genome, which eliminates maternal chromosomes in *Arabidopsis* (Demidov et al., 2022). However, this approach suffers from critical limitations: it requires transgenes in both parents, produces transgenic haploids unsuitable for breeding, and shows very low efficiency compared with established CENH3 manipulation methods in *Arabidopsis*.

A better understanding of the mechanistic basis would enable the rational design of a universal centromere-based haploidization strategy. Cumulative insights from previous studies indicate that CENH3 imbalance between parental genomes during early embryogenesis results in uniparental genome elimination (Sanei et al., 2011; Raychaudhuri et al., 2012; Marimuthu et al., 2021; Wang et al., 2021; Comai and Marimuthu, 2025). Uniparental gametic CENH3 depletion represents a promising strategy to achieve CENH3 imbalance between parental genomes. However, whether targeted gametic CENH3 depletion can successfully induce haploids in plants remains unexplored.

Here, we demonstrate that egg-cell-specific depletion of epitope-tagged CENH3 enables efficient paternal haploid induction in *A. thaliana*. We developed a strategy to specifically deplete EYFP- or ALFA-tagged CENH3 in egg cells using engineered E3 ligases, producing non-transgenic haploids through outcrossing with wild-type plants. To address concerns associated with the use of animal-derived E3 ubiquitin ligases in crop breeding, we successfully replaced them with a plant-derived alternative. This approach achieved haploid induction frequencies of up to 57% in genetic backgrounds previously devoid of haploid induction capability and enhanced the efficiency of existing haploid inducers to up to 72%. We confirmed the strategy's effectiveness by integrating the epitope tag directly into the native CENH3 locus via gene targeting (GT). Moreover, the engineered E3 ligases successfully degraded tagged CENH3 from tomato, demonstrating potential cross-species applicability. These findings establish gametic CENH3 depletion as a systematic, scalable, and potentially universal strategy for centromere-based haploid induction in crop improvement.

RESULTS

EYFP-gCENH3 induces varying levels of haploids

Previously, we utilized a *cenh3* null mutant complemented with an EYFP-tagged CENH3 transgene, *EYFP-gCENH3$^{cenh3\text{-}1}$*, for targeted removal of the EYFP-CENH3 protein in *Arabidopsis* (Demidov et al., 2022). To test whether the gametic depletion of CENH3 results in haploid induction, we utilized *EYFP-gCENH3$^{cenh3\text{-}1}$* as a test genotype. We found that *EYFP-gCENH3$^{cenh3\text{-}1}$* has a background haploid induction activity ranging from 5.54% to 20.34% (Supplemental Table 1). A similar genotype from an independent complementation experiment also resulted in haploid induction frequencies ranging from 6.25% to 14.81% (Supplemental Table 1). However, no haploids were identified in the control cross in the previous study (Demidov et al., 2022). We assume that the low sample size in the control cross in the previous study underlies these contradictory observations.

Marimuthu et al. (2021) suggested that removal of the GFP-tailswap variant in the egg cell is a potential reason for haploid induction. To check whether EYFP-CENH3 is also absent in the egg cell, we examined the egg cells of EYFP-gCENH3$^{cenh3\text{-}1}$. Only 6.73% of egg cells displayed EYFP-CENH3 foci, while all other egg cells showed no detectable EYFP-CENH3 fluorescence (Figure 1A). In contrast, EYFP-CENH3 is detectable in the sperm nuclei of most pollen (Figure 1B). Although most egg cells lack detectable EYFP-CENH3, this results in only up to 19.3% haploids. Therefore, we assumed that EYFP-CENH3 is not completely removed from egg cells and may still be present at levels below the detection limit of microscopy. If this is the case, complete removal of EYFP-CENH3 in gametes should enhance haploid induction frequency.

Egg-cell-specific depletion of the EYFP-CENH3 protein results in shriveled seeds

To test our hypothesis, we employed engineered E3-ligase-based targeted depletion of EYFP-CENH3 in egg cells. These E3 ligases recognize EYFP-CENH3 through a fused GFP-specific nanobody, subsequently marking the protein for ubiquitin-mediated proteasomal degradation via lysine poly-ubiquitination. The E3 ligases were expressed under the control of the *EC1.1* promoter (Sprunck et al., 2012) to degrade EYFP-CENH3 specifically in egg cells. Three different constructs, namely *EV-SPOP*, *EV-NSlmb*, and *EV*, were utilized for this experiment (Figure 2A). The first two bear the E3 ligases *SPOP* (Shin et al., 2015) and *NSlmb* (Caussinus et al., 2011), respectively, fused to a GFP nanobody (*VHHGFP4*) (Rothbauer et al., 2006), and the third one (*EV*) carries the GFP-nanobody

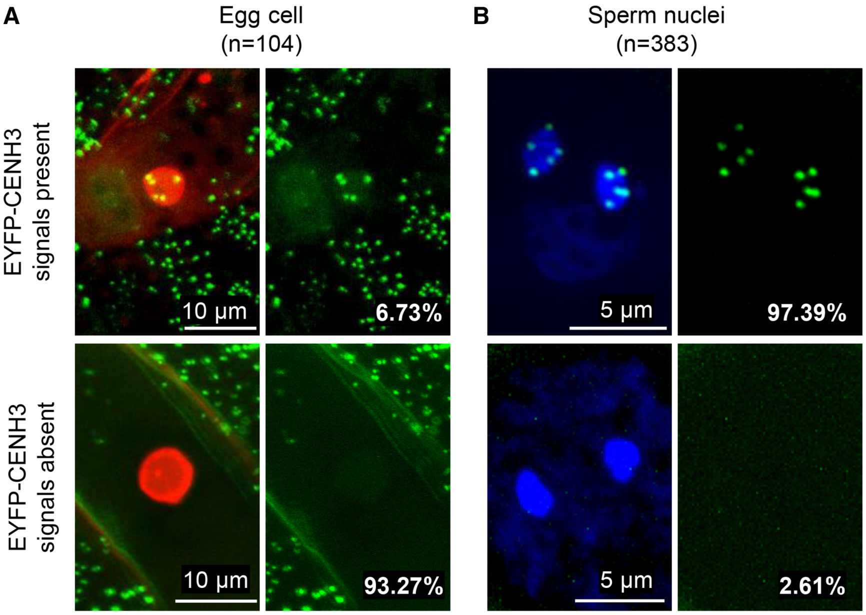

Figure 1. *EYFP-gCENH3$^{cenh3\text{-}1}$* exhibits different levels of EYFP-CENH3 in male and female gametes in *A. thaliana*.

(A) Representative egg cells labeled with histone H2B-tdTomato (red), showing the presence or absence of EYFP-CENH3.

(B) Representative sperm nuclei stained with DAPI (blue), showing the presence or absence of EYFP-CENH3. *n* indicates the number of ovules or pollen analyzed. The frequency of gametes with or without EYFP-CENH3 is given for each scenario.

alone. All three constructs were used to transform *EYFP-gCENH3$^{cenh3\text{-}1}$* plants.

To recover homozygous transgenic lines for these constructs, T2 seeds from 10 independent T1 plants per construct were used for *phosphinothricin* (*PPT*) selection. The frequency of *PPT*-resistant T2 seedlings was significantly lower in *EV-SPOP* and *EV-NSlmb* compared with *EV* (Figure 2B). Upon further investigation, a high number of non-viable, shriveled T2 seeds were derived from *EV-SPOP* and *EV-NSlmb* T1 plants (Supplemental Figure 1). In contrast, no shriveled seeds were obtained from *EV* T1 plants (Supplemental Figure 1). This observation raised the question of whether expression of E3 ligases itself might affect seed development.

To test this possibility, *EYFP-gCENH3^{Wt}* plants were transformed with the constructs described above. Seeds from a few siliques of T1 plants from both genotypes, *EYFP-gCENH3$^{cenh3\text{-}1}$* and *EYFP-gCENH3^{Wt}*, expressing *EV-SPOP*, *EV-NSlmb*, or *EV* were pooled and examined for the prevalence of shriveled seeds. The frequency of shriveled seeds differed significantly between the two genotypes for *EV-SPOP* and *EV-NSlmb* (Figure 2C). In the case of *EV*, no shriveled seeds were observed in either genotype (Figure 2C). This suggests that shriveled seeds were generated only when *EV-SPOP* or *EV-NSlmb* was expressed in the *EYFP-gCENH3$^{cenh3\text{-}1}$* background, likely due to genomic instability during early embryogenesis resulting from reduced abundance of EYFP-CENH3 caused by targeted degradation in egg cells.

Hemizygous T1 plants carrying transgene cassettes with *EV-SPOP* lead to an increased frequency of haploids

The isolation of homozygous transgenic lines through segregation analysis of *PPT* resistance was hindered by the prevalence of shriveled seeds among T2 progeny from plants containing the *EV-SPOP* and *EV-NSlmb* constructs. To investigate whether egg-cell-specific degradation of EYFP-CENH3 could enhance haploid induction frequency, *EYFP-gCENH3$^{cenh3\text{-}1}$* T1 plants hemizygous for the above constructs were used as mother plants and crossed with *gl1-1* fathers in order to use the glabrous phenotype (trichome-less leaves) as a marker for haploid screening (Kuppu et al., 2015). The *EYFP-gCENH3$^{cenh3\text{-}1}$* genotype without a CENH3-degradation construct served as a control.

Evaluation of the offspring from these crosses showed that the *EV-SPOP* construct significantly increased haploid frequency (median 25.17%) compared with the control (median 17.11%), whereas the other constructs were not significantly different from the control (Figure 2D). Although not statistically significant, we observed an increase in haploid induction frequency for *EV* plants (median 23.08%) compared with that of the control. Also, there was no significant difference between T1 plants carrying E3-ligase-bearing constructs (*EV-SPOP* and *EV-NSlmb*) and those with *EV*. Consequently, it remained unclear whether the increase in haploid induction frequency was attributable to the effect of E3 ligases or to the nanobody alone. We hypothesized that variability in the control group contributed to the noise in our data, leading to these ambiguous results. Therefore, isolating a tagged CENH3 line devoid of background haploid induction became necessary to obtain more precise conclusions.

Identification of a non-haploid-inducing ALFA-CENH3 *Arabidopsis* line

To generate a tagged CENH3 variant without background haploid induction, we replaced the EYFP tag (26.7 kDa) with the smaller ALFA protein tag (1.9 kDa) (Gotzke et al., 2019), assuming that the reduced size of the ALFA-tag might eliminate background haploid induction activity. We successfully complemented the *cenh3* null mutant with an *ALFA*-tagged genomic CENH3 transgene, *ALFA-gCENH3* (Supplemental Figure 2). Indirect immunostaining of nuclei with an *ALFA*-specific nanobody and an *Arabidopsis* CENH3-specific antibody revealed centromere-like signals in interphase nuclei (Figure 3A). To assess haploid induction frequency, plants from six independent

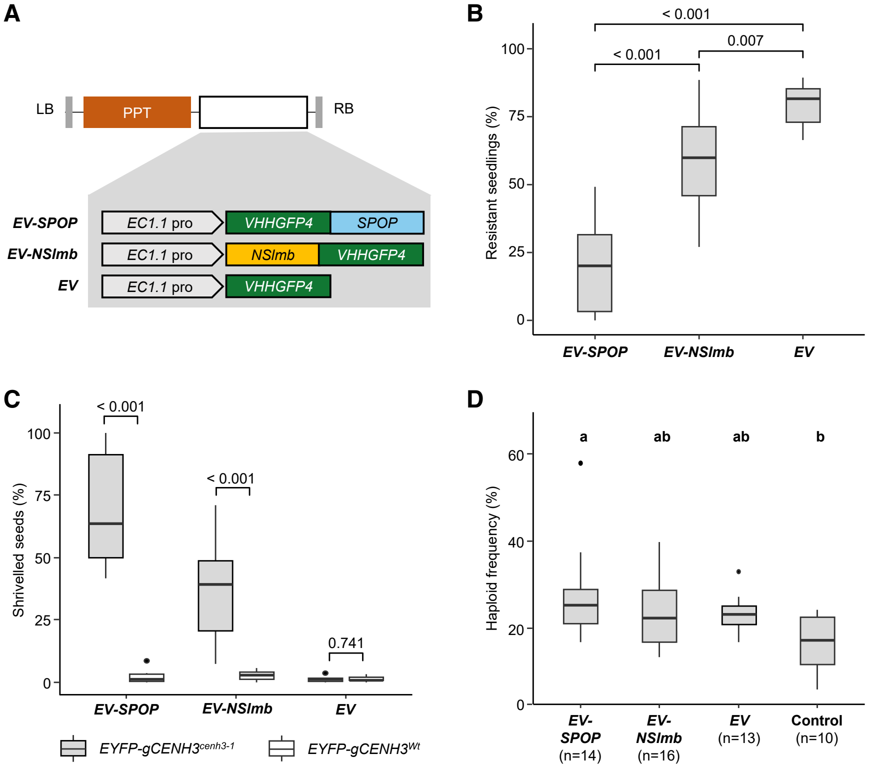

Figure 2. Egg-cell-specific degradation of EYFP-CENH3 results in shriveled seeds and increased haploid induction frequency.

(A) Schematic representation of different constructs (*EV-SPOP*, *EV-NSlmb*, and *EV*) used for egg-cell-specific degradation of EYFP-CENH3. *PPT*, *phosphinothricin*.

(B) Frequency of *PPT*-resistant seedlings obtained from T2 seeds of 10 individual T1 *EYFP-gCENH3$^{cenh3-1}$* plants for the different constructs shown in **(A)**. Comparisons are based on adjusted *p* values calculated using the Holm–Sidak method for three pairwise comparisons.

(C) Frequency of shriveled seeds after self-pollination of 10 different T1 plants from each genotype (*EYFP-gCENH3$^{cenh3-1}$* and *EYFP-gCENH3^{Wt}*) transformed with the constructs shown in **(A)**. *p* values represent the significance of differences between genotypes based on a two-sample *t*-test.

(D) Haploidization frequency of independent *EYFP-gCENH3$^{cenh3-1}$* T1 plants hemizygous for the different constructs shown in **(A)**, after pollination with *gl1-1* pollen. *EYFP-gCENH3$^{cenh3-1}$* plants without any of the constructs were used as controls. *n* indicates the number of individual T1 plants evaluated for each construct and individual plants used as controls.

p values were calculated using the Holm–Sidak method for six pairwise comparisons. A significance threshold of $p < 0.05$ was applied, and different letters denote statistically significant differences in haploid induction frequency between constructs. Detailed data, including the total number of plants screened, haploid counts, and haploid frequencies for each individual cross, are provided in Supplemental Table 9.

ALFA-gCENH3$^{cenh3-1}$ T1 families were pollinated with pollen from *gl1-1* plants. Ten different female plants from each T1 family were used. Haploid frequencies varied widely both within and between T1 families, with an overall haploid frequency ranging from 0% to 15.15% (Figure 3B). Thus, despite the small size of the ALFA-tag, *ALFA-gCENH3$^{cenh3-1}$* plants still induced haploids after outcrossing with wild-type plants. The observed variability suggests that haploid induction ability and efficiency may be influenced by transgene expression levels or positional effects rather than by the ALFA-tag itself.

However, we identified one T1 family, T1-2, in which 4 of 10 T2 plants lacked haploids among the evaluated progeny (Figure 3B). For further analysis, we focused on 1 of these 4 T2 plants, namely T1-2-23. Ten individual T3 plants derived from selfing of T1-2-23 were used as females and crossed with *gl1-1* plants, and the progeny were analyzed for haploid plants. None of the 3,009 assessed F1 plants displayed the glabrous phenotype. Therefore, T3 plants derived from T1-2-23 were used for further transformation with different constructs designed for egg-cell-specific degradation of ALFA-CENH3.

Egg-cell-specific depletion of ALFA-CENH3 leads to haploid induction

For the egg-cell-specific degradation of ALFA-CENH3, four distinct constructs were developed: *EN-SPOP*, *EN-NSlmb*, *EN*, and Control. The *EN-SPOP* and *EN-NSlmb* constructs contain an anti-ALFA nanobody (*NbALFA*) fused to E3 ligases *SPOP* and *NSlmb*, respectively (Figure 3C). The *EN* construct includes only the nanobody (*NbALFA*), while the control construct consists of an empty module. Each construct also integrates a male sterility module (intronized *Barnase*; Hartley, 1988) expressed under the *A9* promoter (Paul et al., 1992) to facilitate crossing, as well as a FAST-Red fluorescent seed marker (Shimada et al., 2010). The fluorescent seed marker was assessed for its suitability in identifying haploid progeny.

ALFA-gCENH3$^{cenh3-1}$ T1 plants hemizygous for any of the above-mentioned constructs were crossed as females with *gl1-1* fathers, and the progeny were evaluated for haploid induction. Crosses with the control construct showed no induction of haploids, confirming that the background does not promote haploid formation (Figure 3D). The nanobody-only construct (*EN*) similarly failed to induce haploids across most lines, with one exception displaying a negligible haploid induction frequency (0.64%) (Figure 3D), suggesting that the anti-*ALFA* nanobody alone is insufficient for effective haploid induction. In contrast, both E3-ligase-fused constructs, *EN-SPOP* and *EN-NSlmb*, resulted in haploid induction, with *EN-SPOP* (median 4.11%) achieving a significantly higher induction frequency of haploids than *EN-NSlmb* T1 plants (1.31%) (Figure 3D). Thus, E3-ligase-mediated degradation of CENH3 in egg cells is effective in inducing paternal haploids when tested in a genetic background confirmed to lack intrinsic haploid-inducing activity. This experimental design ensures that any observed haploid induction can be unambiguously attributed to the introduced degradation module rather than to confounding effects from the tagged CENH3 line itself.

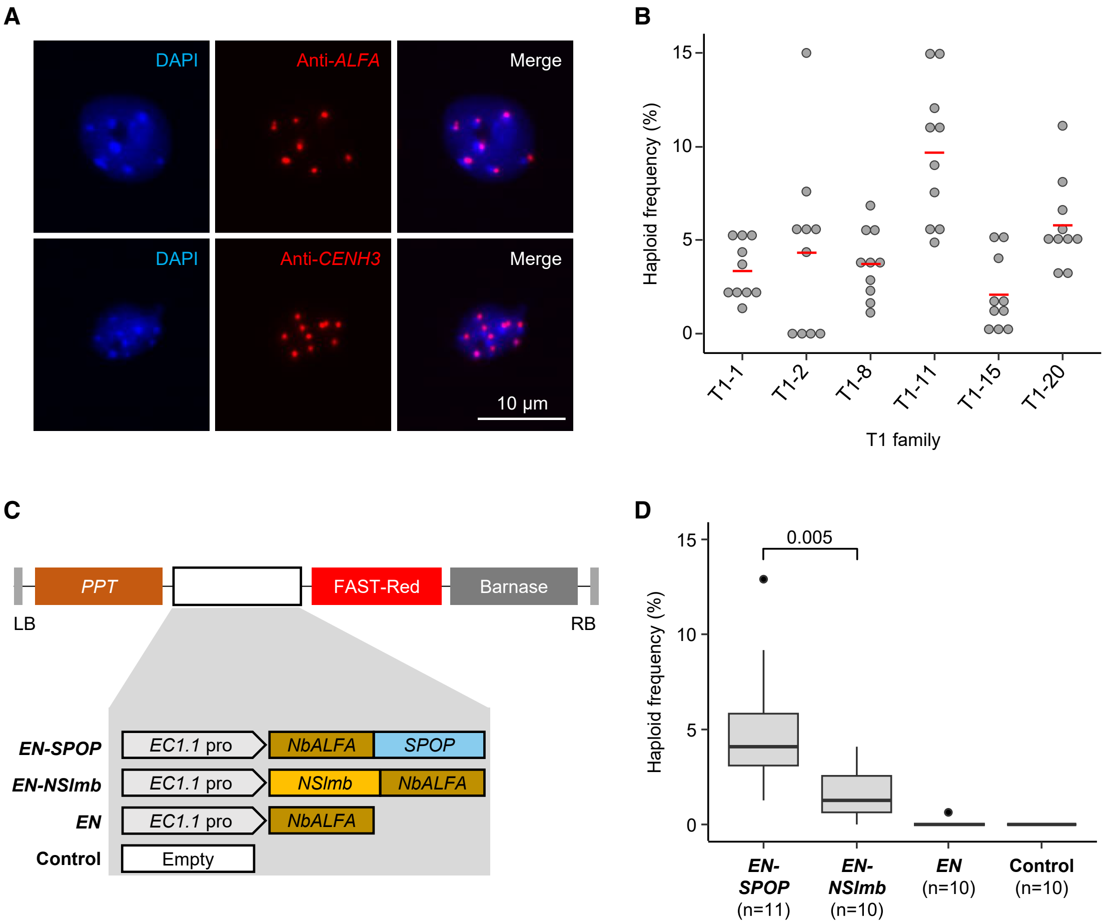

Figure 3. Egg-cell-specific degradation of ALFA-CENH3 by different E3 ligases results in haploid induction.

(A) Immunolabeling of leaf nuclei from *ALFA-gCENH3*$^{cenh3\text{-}1}$ plants with anti-*ALFA* and anti-*CENH3* antibodies. Immunosignals are shown in red. Nuclei were stained with DAPI (blue).

(B) Haploid induction frequencies of 10 females from different *ALFA-gCENH3*$^{cenh3\text{-}1}$ T1 families after outcrossing with *gl1-1* fathers. Gray dots indicate haploid induction frequencies of T2 samples from the corresponding T1 families. Red lines indicate the median haploid induction frequency of the corresponding T1 family. Detailed data, including the total number of plants screened, haploid counts, and haploid frequencies for each individual cross, are provided in Supplemental Table 10.

(C) Schematic representation of different constructs (*EN-SPOP*, *EN-NSlmb*, *EN*, and Control) used for egg-cell-specific degradation of ALFA-CENH3. *PPT*, *phosphinothricin*; FAST-Red, fluorescent seed marker; *Barnase*, intronized *barnase* gene expressed under the control of the *A9* promoter for male sterility.

(D) Haploid induction frequency of independent *ALFA-gCENH3*$^{cenh3\text{-}1}$ T1 plants hemizygous for the different constructs shown in **(C)** after pollination with pollen from *gl1-1* plants. *n* indicates the number of individual T1 plants evaluated for each construct and individual plants used as controls. *p* values are based on a two-sample *t*-test. Detailed data, including the total number of plants screened, haploid counts, and haploid frequencies for each individual cross, are provided in Supplemental Table 11.

Seed marker aids in rapid haploid screening and transgene cassette inheritance tracking

Most previous studies on CENH3-based haploid induction have relied on the glabrous phenotype of the *gl1-1* mutant as a marker for identifying haploids (Kuppu et al., 2015, 2020; Ahmadli et al., 2023; Wang et al., 2023). However, because glabrousness is a recessive trait, its use limits haploid screening to crosses involving *gl1-1* or similar mutants. To address this constraint, we incorporated the dominant FAST-Red fluorescent seed marker (Shimada et al., 2010) into the transgene cassettes for CENH3 depletion. Previous research has shown that FAST-Red enables highly accurate identification of haploid seeds, particularly in *DMP* mutant backgrounds (Zhong et al., 2020).

F1 seeds obtained by crossing *EN-SPOP* with *gl1-1* fathers can be classified into four distinct classes: Class 1, non-fluorescent and morphologically normal; Class 2, bright, uniform red fluorescence; Class 3, faint, uniform red fluorescence; and Class 4, shriveled and collapsed seeds with non-uniform fluorescence (Supplemental Figure 3A and 3B). Class 1 seeds most likely represent seeds originating from gametes lacking the transgene cassette and are therefore wild type, accounting for 38.81%–45.53% (Supplemental Table 2). The frequency of fluorescent seeds (Classes 2 and 3) in *EN-SPOP* crosses was markedly lower, ranging from 12.77% to 19.40% (Supplemental Table 2). This reduction suggests that segregation of the *EN-SPOP* transgene cassette is impaired. Most Class 4 seeds from *EN-SPOP* crosses exhibited residual red fluorescence (Supplemental Figure 3B),

Cross	T1 replicate	Total germinated seedlings	Seedlings from fluorescent seeds	No. of glabrous plants	Haploids based on flow cytometry	Observed haploid frequency (%)	Effective haploid frequency (%)
EN-SPOP × *gl1-1*	1	275	24	6	6	2.18	25.00
	2	448	28	17[a]	16	3.57	57.14
	3	465	36	13	13	2.80	36.11

Table 1. Observed and effective haploid induction frequencies of three independent *EN-SPOP* T1 lines.

Seedlings germinated from seed classes 2, 3, and 4 were considered progeny from fluorescent seeds. Observed haploid induction frequency represents the percentage of haploids among total germinated seedlings. Effective haploid induction frequency represents the percentage of haploids among progeny derived specifically from fluorescent seed classes.

[a]One glabrous seedling was confirmed to be diploid by flow cytometry and was excluded from frequency calculations.

indicating that gametes carrying the *EN-SPOP* construct frequently gave rise to non-viable seeds. This pattern of seed lethality is a well-known consequence of chromosome elimination. In F1 seeds obtained by crossing *EN* T1 plants with *gl1-1*, the majority of seeds belonged to either Class 1 or Class 2 (Supplemental Table 2).

To determine the ploidy status of each seed category, we conducted flow cytometric analysis on nuclei isolated from F1 seeds. All Class 1 seeds were diploid, whereas Class 2 seeds predominantly consisted of diploids, along with a notable proportion of aneuploids and some mixoploids (Supplemental Table 3). Strikingly, Class 3 seeds, which exhibited faint red fluorescence, showed a high frequency of haploids (72.5%), in addition to diploids, aneuploids, and mixoploids. Class 4 seeds did not allow the isolation of nuclei suitable for ploidy determination, likely due to aborted embryo development.

To compare the accuracy of the glabrous phenotype and faint seed fluorescence (Class 3) in identifying haploids, seeds from different classes were germinated and screened. Glabrous plants were recovered exclusively from Class 3 seeds, consistent with our ploidy analysis (Supplemental Table 2). The accuracy of Class 3 seeds in predicting haploids was 92.11%, compared with 97.22% for the glabrous phenotype (Supplemental Table 4). Despite the lower accuracy than that of the glabrous phenotype, the >90% accuracy of Class 3 fluorescence provides a reliable alternative for rapid haploid screening, reducing dependency on the glabrous phenotype.

In addition, we used the FAST-Red seed marker to trace the gametic origin of haploids. Our results showed that haploids occurred exclusively among fluorescent seeds, indicating that they originated from egg cells carrying the transgene cassette with the degradation module, *EN-SPOP* (nanobody with an E3 ligase). In contrast, the *EN* transgene cassette without the degradation module (nanobody without an E3 ligase) showed normal segregation, with no haploid (glabrous) progeny observed among fluorescent seeds (Supplemental Table 2). This confirms that egg-cell-specific CENH3 depletion caused by the degradation module is responsible for haploid induction.

Genome elimination occurs exclusively in the embryo

To understand the differences between bright (Class 2) and faintly fluorescent (Class 3) *EN-SPOP* F1 seeds, we analyzed them using confocal microscopy. In Class 2 seeds, FAST-Red fluorescence was predominantly detected in the embryo and in a single endosperm layer surrounding the embryo (Supplemental Figure 4). In Class 3 seeds, however, fluorescence was confined solely to the endosperm (Supplemental Figure 4). Ravi et al. (2014) reported non-uniform fluorescence patches in haploid seeds from GFP-tailswap mothers, indicating genome elimination in the endosperm. In contrast, we observed uniform fluorescence throughout the endosperm. This difference demonstrates that our approach utilizing the *EC1.1* promoter, restricts CENH3 degradation specifically to the egg cell, thereby confining genome elimination to the embryo rather than extending it to the endosperm.

Accurate assessment of haploid induction requires accounting for transgene transmission

Given that the degradation module (*EN-SPOP*) functions at the gametic level, the predominance of progeny from non-fluorescent Class 1 F1 seeds lacking the transgene (Supplemental Table 2) obscures accurate evaluation of haploid induction efficiency. To address this, we focused specifically on progeny derived from transgenic gametes to determine haploid induction frequency.

The observed haploid induction frequency, calculated as the percentage of haploid plants among all germinated seedlings, ranged from 2.18% to 3.57% (Table 1). This metric underestimates the true performance of the degradation module because it encompasses progeny from both transgenic and non-transgenic gametes, although only the former possess haploid induction capability. Restricting the analysis to seedlings originating from transgenic gametes (seed classes 2, 3, and 4) provided an accurate measure of the module's performance.

The haploid induction frequency, calculated as the percentage of haploid plants among seedlings derived from these seeds, corresponds to the effective haploid induction frequency. Under this refined calculation, the effective haploid induction frequency ranged from 25% to 57.14% (Table 1), demonstrating that the *EN-SPOP* module is capable of inducing haploids at a very high frequency.

The plant E3 ligase EL5 enhances haploid induction frequency compared with SPOP when combined with EYFP-CENH3 but not with ALFA-CENH3

Haploid induction performance differed between the E3 ligases *SPOP* and *NSlmb* (Figure 3D), both derived from non-plant organisms. We hypothesized that a plant-derived

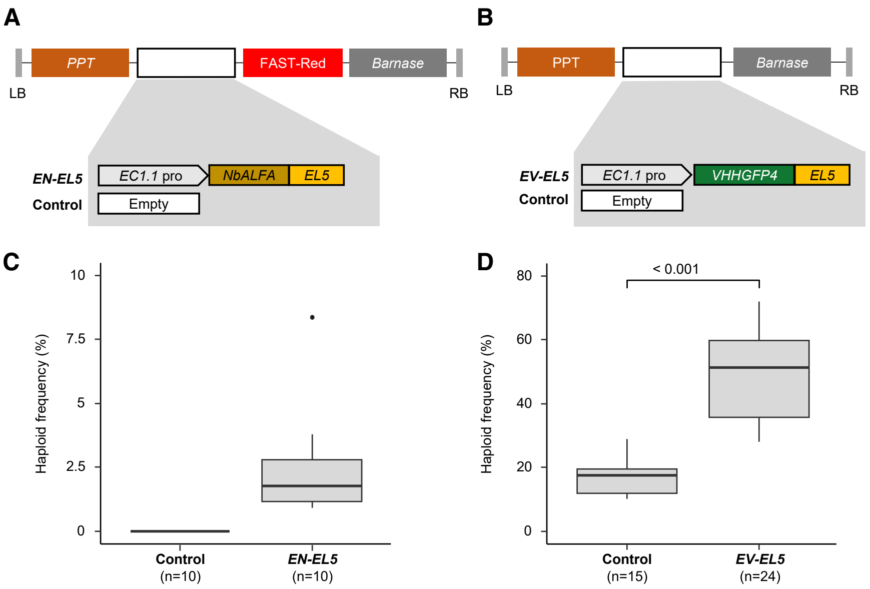

Figure 4. Plant E3 ligase EL5 is more efficient than SPOP only when combined with EYFP-CENH3.

(A) Schematic representation of the different constructs (*EN-EL5* and control) used for the transformation of *ALFA-gCENH3*$^{cenh3\text{-}1}$.

(B) Schematic representation of the different constructs (*EV-EL5* and control) used for the transformation of *EYFP-gCENH3*$^{cenh3\text{-}1}$.

(C and D) Haploidization frequency of independent T1 plants hemizygous for the constructs shown in **(A)** and **(B),** after pollination with *gl1-1* pollen. *n* indicates the number of individual T1 plants evaluated for each construct and individual plants used as controls. The *p* value in **(D)** is based on Welch's *t*-test. Detailed data, including the total number of plants screened, haploid counts, and haploid frequencies for each individual cross, are provided in Supplemental Table 12 for **(C)** and Supplemental Table 13 for **(D)**.

E3 ligase would exhibit enhanced interaction with the plant ubiquitination machinery, thereby improving target protein degradation and the frequency of haploidization. To test this hypothesis, we selected EL5, a well-characterized E3 ligase from *Oryza sativa*. *EL5* encodes a RING-type protein that directly interacts with E2 ubiquitin-conjugating enzymes, promoting efficient substrate ubiquitination (Takai et al., 2002). Katoh et al. (2003) demonstrated that the region spanning amino acids 96–181 is critical for ubiquitination activity.

To assess the functionality of EL5 in mediating CENH3 degradation, we constructed a transgene cassette bearing a degradation module comprising *VHHGFP4* fused to the active domain of EL5 (amino acids 96–181) and histone H2B-mCherry. A transgene cassette without a degradation module was used as the control (Supplemental Figure 5A). Protoplasts isolated from *EYFP-gCENH3*$^{cenh3\text{-}1}$ plants were transfected with these constructs. Fluorescence microscopy analysis revealed a marked reduction in EYFP-CENH3 signals in protoplasts transformed with the *EL5* degradation module, with signal absence observed in most cells (Supplemental Figures 5B and 5C). In contrast, control protoplasts transformed with H2B-mCherry alone retained detectable EYFP-CENH3 fluorescence in most cells (Supplemental Figures 5B and 5C), confirming that the observed signal reduction was specifically attributable to EL5-mediated CENH3 degradation.

To assess its potential for haploid induction, we engineered two independent degradation modules by fusing a functional *EL5* fragment (amino acids 96–181) to *NbALFA* and *VHHGFP4* and placed both fusions under the control of the egg-cell-specific *EC1.1* promoter. The resulting constructs, *EN-EL5* and *EV-EL5*, were introduced into *ALFA-gCENH3*$^{cenh3\text{-}1}$ and *EYFP-gCENH3*$^{cenh3\text{-}1}$ plants, respectively (Figures 4A and 4B). Hemizygous T1 plants carrying either *EN-EL5* or *EV-EL5* were crossed with *gl1-1* males, and the F1 progeny were screened for glabrous individuals as a proxy for haploid induction.

Our results showed that *EN-EL5* is capable of inducing haploids, with a median frequency of 1.76% (Figure 4C). In contrast, T1 lines expressing *EV-EL5* showed a median haploid induction frequency of 51.39%, with values reaching up to 72% (Figure 4D). This represents a significant 2.94-fold increase compared with the control (median 17.5%) (Figure 4D). EL5 (median 51.39%) was more efficient than SPOP (median 25.17%) when combined with EYFP-CENH3. However, this trend was reversed in the context of ALFA-CENH3, where *SPOP* (median 4.11%) outperformed *EL5* (median 1.76%). This indicates that the efficiency of both E3 ligases in terms of haploid induction frequency are differentially influenced by the epitope tag used for CENH3 degradation.

EN-SPOP enables haploid induction in an *in-locus* ALFA-tagged CENH3 line

The variation in haploid induction efficiency among *ALFA-gCENH3*$^{cenh3\text{-}1}$ T1 families suggests that variation in the expression level of modified CENH3, due to transgene positional effects, might contribute to haploid induction capacity. To test this hypothesis, we generated *ALFA-CENH3* knock-in plants by targeted integration of an ALFA tag at the endogenous *CENH3* locus using *ttLbCas12a-i*-mediated *in planta* GT (Figure 5A) (Merker et al., 2020; Schindele et al., 2023). The CRISPR-Cas12a cleavage site was positioned within the start codon to induce a double-strand break, facilitating ALFA-tag integration. The GT donor was designed with a 5′ homology arm of approximately 550 bp containing two silent point mutations to prevent re-cleavage after successful integration and a 3′ homology arm of approximately 580 bp flanking the 45-bp ALFA-tag coding sequence (Supplemental Data 1). Within this donor construct, the ALFA-tag was precisely placed in-frame at the N terminus of *At*CENH3 to ensure accurate tagging upon homologous recombination. To enable donor excision, additional CRISPR-Cas12a target sites, including protospacer adjacent motif (PAM) sequences, were placed at both ends of the GT donor (Figure 5B).

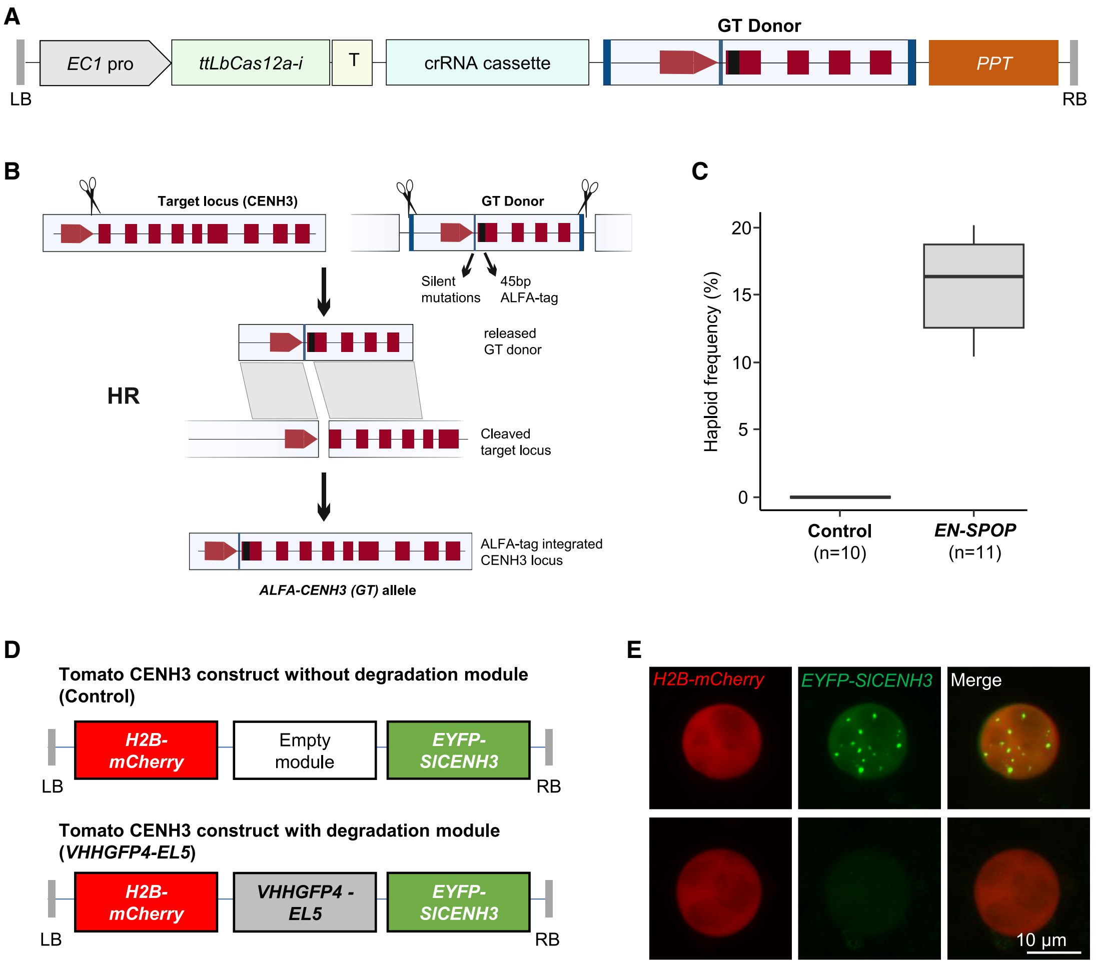

Figure 5. Functionality of degradation modules in the *ALFA-CENH3* (*GT*) background and for tomato CENH3 degradation.
(A) Schematic representation of the T-DNA construct used for *in planta* gene targeting (*ip*GT). Cas12a expression was driven by an egg-cell-specific promoter (*EC1pro*) using the intronized tt*Lb*Cas12a variant (*ttLbCas12a-i*) together with a crRNA cassette targeting *AtCENH3* and a GT donor flanked by target sites for donor excision. The GT donor consists of 5′ and 3′ homology arms and includes the 45-bp ALFA-tag coding sequence, with silent mutations introduced to prevent re-cleavage after successful integration. The phosphinothricin resistance cassette (PPT) was used as a selection marker.
(B) Overview of the *ip*GT strategy for inserting the ALFA-tag at the wild-type *CENH3* locus. Cas12a induces a double-strand break at the target site within the start codon and, at the same time, excises the GT donor from the vector to be used as a template for homologous recombination (HR). HR results in precise insertion of the ALFA tag immediately downstream of the start codon, generating an ALFA-tagged *CENH3* allele.
(C) Haploidization frequency of *ALFA-CENH3* (*GT*) plants and independent hemizygous *EN-SPOP* T1 plants in the *ALFA-CENH3* (*GT*) background after pollination with *gl1-1* pollen. *n* indicates either the number of individual T1 plants evaluated for *EN-SPOP* or the number of individual plants used as controls. Detailed data, including the total number of plants screened, haploid counts, and haploid frequencies for each individual cross, are provided in Supplemental Table 14.
(D) Schematic representation of transgene cassettes with and without the degradation module (*VHHGFP4-EL5*), carrying H2B-mCherry and EYFP-*Sl*CENH3 (tomato), used for transient transformation in *N. benthamiana*.
(E) Representative nuclei of *N. benthamiana* labeled with histone H2B-mCherry (red) and showing the presence or absence of EYFP-*Sl*CENH3 (green).

Screening of 2400 T2 plants identified one heterozygous plant carrying the correctly targeted ALFA-tag insertion, corresponding to a GT efficiency of 0.04%. From its progeny, plants with a homozygous ALFA tag insertion were identified by PCR and used for further experiments. Molecular analysis confirmed precise integration of the ALFA tag at the target locus (Supplemental Figure 6). This allele of *CENH3* is termed the "*ALFA-CENH3* (*GT*)" allele. Plants homozygous for the *ALFA-CENH3* (*GT*) allele were isolated, and they exhibited no morphological differences compared with wild-type Col-0. Anti-*ALFA* immunostaining confirmed the functionality and centromeric localization of the ALFA-CENH3 protein (Supplemental Figure 7).

To assess haploid induction capacity, homozygous *ALFA-CENH3* (*GT*) plants were crossed with *gl1-1* males, and F1 progeny were screened for glabrous haploid plants. No glabrous plants were detected among the progeny, indicating that *ALFA-CENH3* (*GT*) plants lack haploid-inducing activity (Figure 5C). These results demonstrate that ALFA tagging of CENH3 alone is insufficient for haploid induction and instead suggest that altered gene regulation associated with the *ALFA-gCENH3*

transgene in the *cenh3-1* background may be responsible for this haploidization phenotype.

To determine whether egg-cell-specific degradation of *in-locus*-tagged CENH3 in this background could lead to haploid induction, we transformed ALFA-CENH3 (GT) plants with the *EN-SPOP* construct, which was selected based on its high induction efficiency in the *ALFA-gCENH3*$^{cenh3\text{-}1}$ background. Hemizygous *EN-SPOP* T1 plants in the *ALFA-CENH3* (*GT*) background were crossed with *gl1-1* pollen donors, and the progeny were screened for haploid induction. Glabrous haploids were recovered from all T1 maternal lines, with a median haploid induction frequency of 16.38% (Figure 5C). These findings conclusively demonstrate that egg-cell-specific degradation of epitope-tagged CENH3 is sufficient for haploid induction, regardless of whether the tagged CENH3 is expressed from a transgene or the tag is integrated at the endogenous *CENH3* locus.

Engineered plant-derived EL5 effectively degrades tagged tomato CENH3

Translation of egg-cell-specific CENH3 degradation technology from *Arabidopsis* to crop species requires validation that the engineered rice-derived E3 ligase fusion protein can effectively degrade CENH3 orthologs in heterologous systems. As an initial test case, we selected tomato (*Solanum lycopersicum*) *CENH3* (*SlCENH3*) to evaluate the functionality of the engineered E3 ligase. We generated two constructs for transient expression in *Nicotiana benthamiana*: the first contained modules for constitutive expression of *EYFP-SlCENH3* and histone H2B-mCherry, both driven by the *35S* promoter; the second construct included an additional degradation module expressing EL5-VHHGFP4 under the control of the *35S* promoter (Figure 5D).

Following agroinfiltration, confocal microscopy revealed characteristic centromeric EYFP foci in all H2B-mCherry signal-positive nuclei (n = 38) transformed with the construct lacking the degradation module. In contrast, most H2B-mCherry-positive nuclei (96%, n = 50) transformed with the construct containing *EYFP-SlCENH3* and the corresponding degradation module showed a nearly complete absence of EYFP foci (Figure 5E). These results demonstrate that the plant-based engineered E3 ligase EL5 is effective at degrading tagged CENH3 from species other than *Arabidopsis*.

DISCUSSION

We developed a CENH3 degradation-based haploidization approach that produces wild-type haploids. Haploid inducers were generated by combining engineered (non-plant or plant-derived) E3 ligases with YFP- or ALFA-tagged CENH3. These haploid inducers undergo egg-cell-specific CENH3 degradation, and after outcrossing with wild-type plants, maternal chromosomes are eliminated in a subset of F1 plants, resulting in paternal, non-transgenic haploids (Figure 6). An important advantage of this approach is its scalability, allowing increased haploid induction frequency through the use of more efficient protein degradation systems.

Our findings suggest that extreme differences in CENH3 levels between parental chromosomes during early embryogenesis are critical for effective haploid induction. While gametic CENH3 dilution through heterozygous null mutations is effective in maize, its efficacy is significantly reduced in Brassicaceae and largely ineffective in wheat (Lv et al., 2020; Marimuthu et al., 2021; Wang et al., 2021, 2024). This species-specific variation in tolerance to parental CENH3 asymmetry underscores the need for strategies that actively reduce gametic CENH3 levels for broad applicability.

RNAi-mediated CENH3 downregulation has limitations, as constitutive CENH3 suppression compromises plant fitness because of its essential role in chromosome segregation (Lermontova et al., 2011b; Manape et al., 2024). Recent studies show that CENH3 RNAi driven by constitutive *RPS5A* promoters induces haploids only under elevated temperatures or enhances existing inducer frequency (Yang et al., 2025). However, RNAi driven by gametic promoters, such as *EC1.2*, fails to effectively induce haploids (Yang et al., 2025), likely due to CENH3's intrinsic stability and low turnover in centromeric chromatin (Lermontova et al., 2011a). These findings indicate that RNAi alone is insufficient to deplete centromeric CENH3 in gametes, necessitating active protein-degradation mechanisms.

Here, we demonstrate that the ubiquitin–proteasome pathway can selectively degrade CENH3 in egg cells using engineered E3 ubiquitin ligases, generating paternal haploids upon wild-type outcrossing. Since targeted Cid depletion (the *Drosophila* CENH3 homolog) in sperm cells induces gynogenic haploids in *Drosophila* (Raychaudhuri et al., 2012), the link between CENH3 asymmetry and haploid induction appears to be evolutionarily conserved. Therefore, gametic CENH3 degradation offers a promising strategy for haploid induction across crop species.

Using *EN-SPOP* combined with ALFA-CENH3, we achieved an observed haploid induction frequency of 4.11% (median). This frequency appears to be lower than that of the GFP-tailswap modification (29%–45%) (Ravi et al., 2010). Unlike other CENH3-based modifications in *Arabidopsis*, our approach uniquely restricts haploid induction potential to gametes carrying the transgene encoding the degradation module. As the haploid induction experiments in this study used T1 plants hemizygous for CENH3 degradation constructs, efficiency should be compared based on effective rather than observed haploid induction frequency. Leveraging the FAST-Red seed marker, we show that the effective haploid induction frequency of *EN-SPOP* with ALFA-CENH3 ranges from 25% to 57%. This frequency reflects the haploid induction potential of homozygous lines carrying the *EN-SPOP* transgene cassette.

Interestingly, SPOP, which induces haploids at higher frequencies than NSlmb, also causes correspondingly higher seed lethality upon self-pollination. However, this seed lethality associated with high-efficiency haploid induction, which impedes standard segregation analysis, challenges the isolation of homozygous lines. The fluorescent seed marker system proves essential in this context, enabling the identification and selection of transgene-positive seeds among the self-pollinated progeny of hemizygous lines, thereby facilitating the maintenance and propagation of haploid-inducing genotypes.

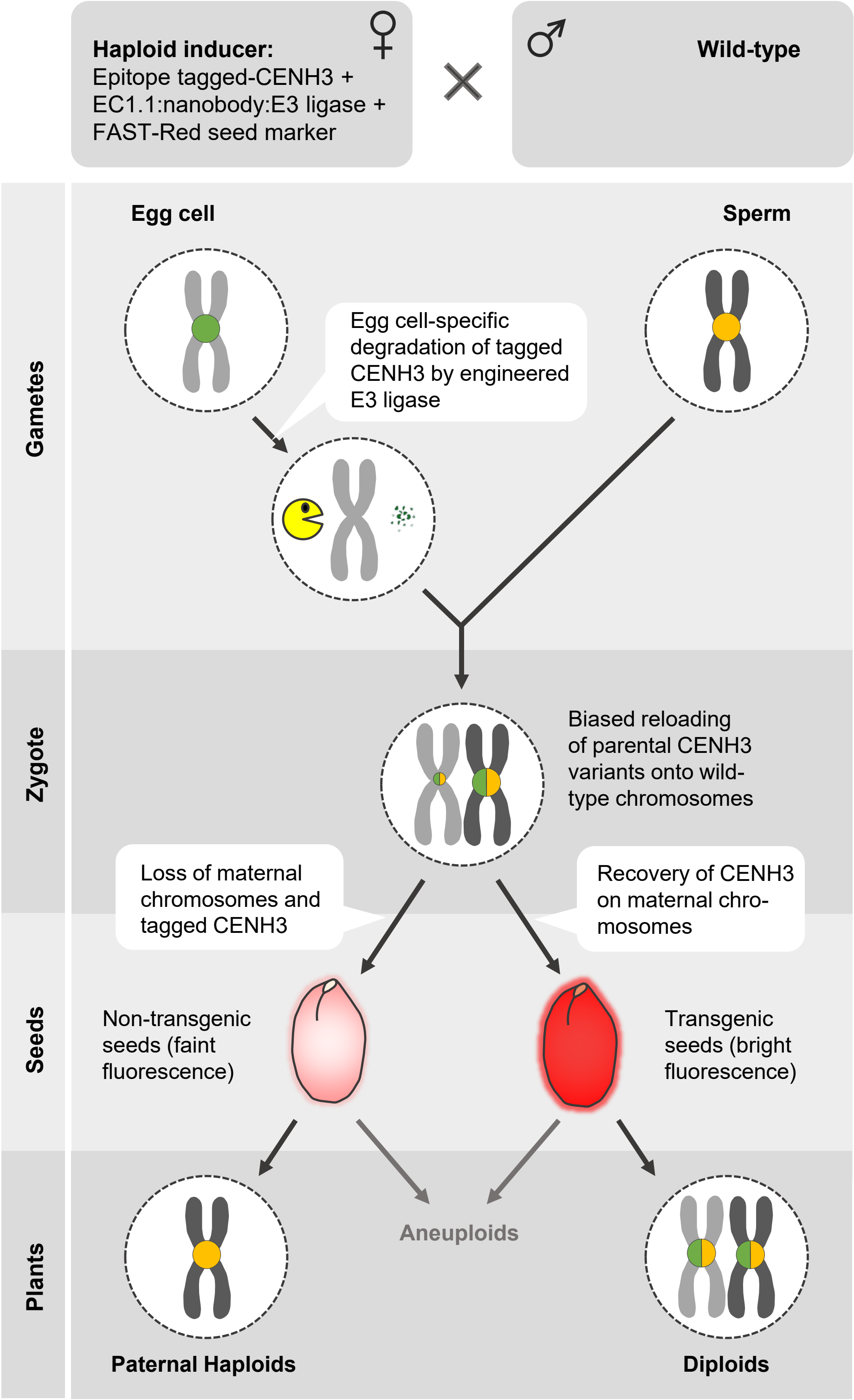

Figure 6. Schematic model of egg-cell-specific CENH3 depletion for paternal haploid induction.
The haploid inducer line carries epitope-tagged CENH3 and is transformed with constructs containing (i) an E3 ligase module for egg-cell-specific CENH3 degradation and (ii) a FAST-Red seed marker module. Gametes: targeted degradation of tagged CENH3 generates maternal chromosomes depleted of CENH3 nucleosomes. Zygote: fertilization with a wild-type sperm produces a zygote containing parental chromosomes with pronounced CENH3 nucleosome asymmetry. Both wild-type and epitope-tagged CENH3 preferentially reload onto CENH3-enriched paternal chromosomes. Seeds: this asymmetric zygote generates two distinct seed fluorescence classes: seeds with faint fluorescence (endosperm-derived signal only) develop into non-transgenic paternal haploids and a few aneuploids, whereas seeds with bright fluorescence (combined embryo and endosperm signals) develop into transgenic diploids and aneuploids.

This is the first study evaluating plant-derived engineered E3 ligases for targeted protein degradation in plants, whereas previous studies used only non-plant-organism-derived E3 ligases (Baudisch et al., 2018; Sorge et al., 2021; Huang and Rojas-Pierce, 2024). However, OsEL5 proved more efficient than *SPOP* in haploid induction only when combined with EYFP-CENH3 and showed the opposite trend with ALFA-CENH3. Previous studies demonstrated that lethality caused by a ubiquitination-deficient mutant, CENP-A K124R, could be rescued by EYFP-tagged CENP-A K124R but not by FLAG-K124R (Niikura et al., 2019), indicating that different tags can influence ubiquitination potential. Additionally, E3 ligase degradation efficiency can vary depending on the interacting domain to which it is fused, likely due to context-dependent steric hindrance (Huang and Rojas-Pierce, 2024). These findings suggest that EYFP's larger structure compared with the ALFA tag may offer more accessible lysine residues or a better structural context for ubiquitination by the monomeric E3 ligase *EL5*. In contrast, SPOP, as a multi-subunit E3 ligase complex, may achieve an optimal geometry for effective ubiquitination of ALFA-tagged CENH3. The interplay among substrate accessibility, steric constraints, and the oligomeric state of the E3 ligase complex appears to be a critical determinant of degradation efficiency. These factors must be considered in future optimizations of protein degradation system design to identify suitable components for efficient degradation.

Applying our approach to crop plants requires two key steps: generating genotypes with a functional epitope-tagged CENH3 to facilitate the recruitment of CENH3-specific E3 ligases and transforming the tagged CENH3 line with constructs for gametic expression of engineered E3 ligases. Epitope-tagged CENH3 genotypes can be generated through two methods: complementing a *cenh3* null mutant with an epitope-tagged CENH3 transgene or using an *in-locus* protein tagging strategy to insert the tag directly at the endogenous locus. Our results demonstrate that the CENH3 degradation strategy functions effectively irrespective of the method used to generate the tagged CENH3 line. Furthermore, the *in-locus*-tagged CENH3 was helpful in bypassing potential position effects associated with transgenic CENH3.

While these steps involve transgenesis, the resulting haploids remain non-transgenic, making this strategy compatible with breeding programs. However, whether these haploids would be subject to regulatory oversight remains debatable and may depend on the country. We endeavored to assess plant E3 ligases for developing effective haploid inducers, as public sentiment may be more averse to using genetic elements from animal species than from plants (Weale, 2010).

Paternal haploids carry the cytoplasm of the female parent and are useful for cytoplasm swapping to generate male-sterile lines (Bortiri et al., 2024; Han et al., 2024). However, haploids that carry both the cytoplasm and the nuclear genome of the maternal parent are preferred in breeding programs (Wang et al., 2023). Therefore, exploring whether sperm-specific degradation of CENH3 can induce maternal haploids in the future would be of interest. However, identifying a promoter that is exclusively active in sperm nuclei that fertilize egg cells remains a challenge (Ingouff et al., 2009). Promising candidates for driving sperm-specific CENH3 degradation include promoters from genes such as *GEX2*, *GCS1*, and *MGH3* (Borg et al., 2011). The expression of these genes has been shown to be highly specific to the generative nucleus and sperm cells.

The combination of plant EL5 and EYFP-CENH3 has proven highly effective for haploid induction in *Arabidopsis*. Notably, the same combination was also successful for the degradation of transiently expressed tomato EYFP-CENH3. Thus, targeted degradation of CENH3 holds strong potential for translation to crop species.

Additionally, alternative protein degradation strategies could be explored to further improve haploid induction frequencies. Incorporating seed markers such as FAST-Red greatly facilitates rapid and efficient haploid screening. Notably, restricting genome elimination to embryos represents a major advancement, particularly for monocot species that require embryo rescue for haploid recovery. The gamete-specific CENH3 degradation strategy presented here marks a starting point for this line of research. Further optimization of components, including promoters, E3 ligases, and other factors, in alignment with synthetic biology principles, may further enhance haploid induction efficiency.

METHODS

Plant growth conditions

Arabidopsis plants were cultivated under long-day conditions (16 h light/8 h dark) at 21°C in a cultivation room. Plants used for crossings were moved to a plant growth chamber (Percival) maintained at 21°C, 16 h light/8 h dark, 2 weeks before emasculation to ensure a constant temperature until complete seed set. Transgenic seeds were selected on ½ MS (Murashige and Skoog, 2006) medium supplemented with cefotaxime (100 μg/ml) and one of the following selection agents: kanamycin (50 μg/ml), hygromycin (15 μg/ml), or PPT (20 μg/ml), under long-day conditions (16 h light/8 h dark) at 21°C. *Arabidopsis* plants were stably transformed using the floral dip method as described by Clough and Bent (1998). Protoplast preparation and transformation were performed following the protocol described by Wu et al. (2009).

Genotypic description

The *cenh3-1* mutation in the Col-0 genetic background (Ravi et al., 2010) was used as a null allele in this study. *EYFP-gCENH3* refers to a previously described transgenic CENH3 construct (Le Goff et al., 2020). *ALFA-gCENH3* denotes a transgenic construct containing genomic CENH3 sequences with a nucleotide sequence encoding the ALFA tag inserted immediately after the start codon. Expression of both transgenic CENH3 constructs is driven by the native *CENH3* promoter and terminator. The *cenh3-1* mutation was genotyped using a dCAPS marker based on *Eco*RV digestion of PCR products (Ravi et al., 2010). Primer sequences varied depending on the transgenic CENH3 construct and are provided in Supplemental Table 5.

EYFP-gCENH3$^{cenh3\text{-}1}$ represents a homozygous *cenh3-1* null mutant complemented by the *EYFP-gCENH3* transgene. *EYFP-gCENH3*^{Wt} refers to wild-type (Col-0) plants expressing the *EYFP-gCENH3* transgene. FAST-Red refers to the pOLE1:OLE1-tagRFP construct encoding seed fluorescence (Shimada et al., 2010). *ALFA-gCENH3*$^{cenh3\text{-}1}$ refers to the *cenh3-1* null mutant complemented by the ALFA-gCENH3 transgene. *ALFA-CENH3* (*GT*) refers to plants homozygous for an *in-locus* ALFA-tagged CENH3 allele.

Preparation of transgenic constructs

All transgenic constructs, except the egg cell marker, were prepared using the Golden Gate-based modular cloning (MoClo) tool kit (Engler et al., 2014). The pICSL4723 vector served as the level 2 destination binary vector. Golden Gate reactions were performed following the protocol described by Grützner and Marillonnet (2020). ALFA-gCENH3 was prepared by assembling two PCR amplicons carrying *Bsa*I sites into pICH86966. PCR amplicons were generated from *Arabidopsis* genomic DNA using the primers listed in Supplemental Table 5. The *ALFA-gCENH3* plasmid and all level 0 modules generated in this study, along with their Addgene ID numbers, are listed in Supplemental Table 6. Details of the modules used to construct level 1 and level 2 plasmids can be found in Supplemental Tables 7 and 8, respectively.

For the egg cell marker *EC1.1pro:H2B-tdTomato*, entry clones containing the *EC1.1* promoter, the H2B-tdTomato coding sequence, and the rbcSE9 terminator were re-combined into pGWB501 using multi-site Gateway cloning (LR Clonase II Plus enzyme, Thermo Fisher). The destination and entry vectors used for GT were based on the previously described *pDe-EC-ttLbCas12a-i* and *pEn-RZ-LbcrRNA* plasmids (Schindele et al., 2023). The GT donor was synthesized by BioCat (Heidelberg, Germany), and flanking *Spe*I restriction sites were added for integration into the destination vector. Specification of the crRNA cassette and final assembly of the *in planta* GT expression vector were performed as previously described (Schindele et al., 2023).

Gene targeting for *in-locus* ALFA tagging of CENH3

T1 plants transformed with the *in planta* GT expression vector were grown, and T2 seeds were harvested individually from each line. A bulk screening method (Ronspies et al., 2022) was performed on T2plants using NC368 and SY209 primers, followed by individual line screening of positive bulks with primers NC368 and NC369. Correct integration was confirmed by Sanger sequencing (Eurofins Genomics, Germany) using primer NC370. PCR and sequence analyses were conducted to identify homozygous ALFA-tag-positive lines. Primer sequences are provided in Supplemental Table 5.

Crossing and haploid screening

Crossing and haploid screening were performed as described by Kuppu et al. (2015). The *gl1-1* mutation, which confers a recessive glabrous (trichome-less) phenotype in the *Ler* background, was used as a phenotypic marker for haploid screening. Plants for which haploid induction frequency was to be quantified were crossed with *gl1-1* plants, and seedlings were screened for glabrous plants 3 weeks after sowing. To confirm ploidy, a few randomly selected plants were then analyzed using flow cytometry.

Transient transformation in *N. benthamiana*

Constructs were transformed into *Agrobacterium tumefaciens* strain GV3101. Agrobacteria harboring the constructs were cultured overnight at 28°C in YEB medium containing kanamycin (50 mg/l), gentamicin (30 mg/l), and rifampicin (50 mg/l) for transient transformation of *N. benthamiana* according to Phan and Conrad (2016).

Indirect immunostaining

Indirect immunostaining was performed on fixed leaf nuclei using the protocol described by Kuo et al. (2023). Custom-made rabbit anti-*Arabidopsis* CENH3 antibodies and a recombinant ALFA nanobody (*NbALFA*) fused to a rabbit IgG Fc domain (NanoTag Biotechnologies, #N1583) were used as primary antibodies. Anti-rabbit secondary antibodies conjugated to a rhodamine fluorophore were used for fluorescent labeling.

Microscopy

Ovules were mounted on glass slides using 50% glycerol/0.1× PBS. DAPI staining of mature pollen was performed following a method described before (Park et al., 1998). Ovules, DAPI-stained pollen, protoplasts, mature *Arabidopsis* seeds, and transiently transformed *N. benthamiana* nuclei were analyzed using a confocal laser scanning microscope (Zeiss LSM780). Images were captured as z-stacks, and maximum-intensity projections were generated in Fiji (ImageJ). Egg cells within ovules were identified using the H2B-tdTomato marker driven by the *EC1.1* promoter.

Immunostained *Arabidopsis* leaf nuclei were imaged using an Olympus BX61 epifluorescence microscope equipped with a Hamamatsu Orca ER CCD camera. Pooled seeds were imaged using a Zeiss Axio Zoom stereo microscope.

Flow cytometry

A BD Influx cell sorter (Becton Dickinson) was used to analyze ploidy. Either young leaves or mature seeds were finely chopped with a razor blade in CyStain UV Ploidy buffer (Sysmex). The homogenate was filtered through a 50-µm nylon mesh and analyzed by flow cytometry.

Statistical comparisons

For all statistical comparisons, the methods used for significance testing, along with p values and sample sizes (n), are provided within the figures or detailed in the figure legends.

FUNDING

This research was supported by grants from DAAD (funding program ID 57507871, reference number 91767722) to S.S. and from Bayer Crop Science (USA) to A.H. S.Y. gratefully acknowledges financial support from the Study Abroad Postgraduate Education Scholarship Program (YLSY) awarded by the Republic of Türkiye Ministry of National Education.

ACKNOWLEDGMENTS

The MoClo toolkit was a gift from Sylvestre Marillonnet (IPB, Halle; Addgene kit #1000000044). pICSL4723 was a gift from Mark Youles (TSL, UK). We thank Luca Comai, Mohan P.A. Marimuthu (UC, Davis, USA), and Stefan Heckmann (IPK, Germany) for critical discussions of the manuscript; Armin Meister for his input on statistical analysis; and Twan Rutten (IPK, Germany) for assistance with seed microscopy. No conflict of interest is declared.

AUTHOR CONTRIBUTIONS

S.S. performed most of the experiments with technical assistance from O.W. J.F. performed the flow cytometry experiments. J.C. contributed to microscopy experiments, and T.L. contributed to crossing and haploid screening. M.C. generated and characterized *EYFP-gCENH3* transgenic lines. A.K. contributed to aspects of experimental design. S.Y., under the supervision of N.C. and H.P., performed all experiments related to *in planta* gene targeting. J.C.L. contributed to the selection and characterization of plant-derived E3 ligases. A.H. supervised the research project. S.S., J.F., and A.H. wrote the manuscript with input from all co-authors.

SUPPLEMENTAL INFORMATION

Supplemental information is available at *Plant Communications Online*.

Received: September 19, 2025
Revised: February 16, 2026
Accepted: February 26, 2026
Published: March 30, 2026

REFERENCES

Ahmadli, U., Kalidass, M., Khaitova, L.C., Fuchs, J., Cuacos, M., Demidov, D., Zuo, S., Pecinkova, J., Mascher, M., Ingouff, M., et al. (2023). High temperature increases centromere-mediated

genome elimination frequency and enhances haploid induction in Arabidopsis. Plant Commun. **4**:100507. https://doi.org/10.1016/j.xplc.2022.100507.

Baudisch, B., Pfort, I., Sorge, E., and Conrad, U. (2018). Nanobody-directed specific degradation of proteins by the 26S-proteasome in plants. Front. Plant Sci. **9**:130. https://doi.org/10.3389/fpls.2018.00130.

Borg, M., Brownfield, L., Khatab, H., Sidorova, A., Lingaya, M., and Twell, D. (2011). The R2R3 MYB transcription factor DUO1 activates a male germline-specific regulon essential for sperm cell differentiation in Arabidopsis. Plant Cell **23**:534–549. https://doi.org/10.1105/tpc.110.081059.

Bortiri, E., Selby, R., Egger, R., Tolhurst, L., Dong, S., Beam, K., Meier, K., Fabish, J., Delaney, D., Dunn, M., et al. (2024). Cyto-swapping in maize by haploid induction with a cenh3 mutant. Nat. Plants **10**:567–571. https://doi.org/10.1038/s41477-024-01630-1.

Caussinus, E., Kanca, O., and Affolter, M. (2011). Fluorescent fusion protein knockout mediated by anti-GFP nanobody. Nat. Struct. Mol. Biol. **19**:117–121. https://doi.org/10.1038/nsmb.2180.

Clough, S.J., and Bent, A.F. (1998). Floral dip: a simplified method for Agrobacterium-mediated transformation of *Arabidopsis thaliana*. Plant J. **16**:735–743. https://doi.org/10.1046/j.1365-313x.1998.00343.x.

Comai, L., and Marimuthu, M.P.A. (2025). Pioneers of chromosome elimination. Front. Epigenetics Epigenomics. **3**:1648270. https://doi.org/10.3389/freae.2025.1648270.

Demidov, D., Lermontova, I., Moebes, M., et al. (2022). Haploid induction by nanobody-targeted ubiquitin-proteasome-based degradation of EYFP-tagged CENH3 in *Arabidopsis thaliana*. J. Exp. Bot. **73**:7243–7254. https://doi.org/10.1093/jxb/erac359.

Engler, C., Youles, M., Gruetzner, R., Ehnert, T.M., Werner, S., Jones, J.D.G., Patron, N.J., and Marillonnet, S. (2014). A golden gate modular cloning toolbox for plants. ACS Synth. Biol. **3**:839–843. https://doi.org/10.1021/sb4001504.

Gao, X., Guo, H., Wu, J., Fan, Y., Zhang, L., Guo, H., Lian, X., Fan, Y., Gou, Z., Zhang, C., et al. (2020). Haploid Bio-Induction in Plant through Mock Sexual Reproduction. iScience **23**:101279. https://doi.org/10.1016/j.isci.2020.101279.

Götzke, H., Kilisch, M., Martínez-Carranza, M., Sograte-Idrissi, S., Rajavel, A., Schlichthaerle, T., Engels, N., Jungmann, R., Stenmark, P., Opazo, F., and Frey, S. (2019). The ALFA-tag is a highly versatile tool for nanobody-based bioscience applications. Nat. Commun. **10**:4403. https://doi.org/10.1038/s41467-019-12301-7.

Grützner, R., and Marillonnet, S. (2020). Generation of MoClo standard parts using Golden Gate cloning. In DNA Cloning and Assembly: Methods and Protocols, S. Chandran and K.W. George, eds. (Springer US), pp. 107–123.

Han, F., Zhang, X., Liu, Y., Liu, Y., Zhao, H., and Li, Z. (2024). One-step creation of CMS lines using a BoCENH3-based haploid induction system in Brassica crop. Nat. Plants **10**:581–586. https://doi.org/10.1038/s41477-024-01643-w.

Hartley, R.W. (1988). Barnase and barstar. Expression of its cloned inhibitor permits expression of a cloned ribonuclease. J. Mol. Biol. **202**:913–915. https://doi.org/10.1016/0022-2836(88)90568-2.

Huang, L., and Rojas-Pierce, M. (2024). Rapid depletion of target proteins in plants by an inducible protein degradation system. Plant Cell **36**:3145–3161. https://doi.org/10.1093/plcell/koae072.

Ingouff, M., Sakata, T., Li, J., Sprunck, S., Dresselhaus, T., and Berger, F. (2009). The two male gametes share equal ability to fertilize the egg cell in *Arabidopsis thaliana*. Curr. Biol. **19**:R19–R20. https://doi.org/10.1016/j.cub.2008.11.025.

Jacquier, N.M.A., Calhau, A.R.M., Fierlej, Y., et al. (2023). *In planta* haploid induction by kokopelli mutants. Plant Physiol. **193**:182–185. https://doi.org/10.1093/plphys/kiad328.

Jin, C., Sun, L., Trinh, H.K., and Danny, G. (2023). Heat stress promotes haploid formation during CENH3-mediated genome elimination in Arabidopsis. Plant Reprod. **36**:147–155. https://doi.org/10.1007/s00497-023-00457-8.

Karimi-Ashtiyani, R., Ishii, T., Niessen, M., Stein, N., Heckmann, S., Gurushidze, M., Banaei-Moghaddam, A.M., Fuchs, J., Schubert, V., Koch, K., et al. (2015). Point mutation impairs centromeric CENH3 loading and induces haploid plants. Proc. Natl. Acad. Sci. USA **112**:11211–11216. https://doi.org/10.1073/pnas.1504333112.

Katoh, S., Hong, C., Tsunoda, Y., Murata, K., Takai, R., Minami, E., Yamazaki, T., and Katoh, E. (2003). High precision NMR structure and function of the RING-H2 finger domain of EL5, a rice protein whose expression is increased upon exposure to pathogen-derived oligosaccharides. J. Biol. Chem. **278**:15341–15348. https://doi.org/10.1074/jbc.M210531200.

Kelliher, T., Starr, D., Wang, W., et al. (2016). Maternal haploids are preferentially induced by CENH3-tailswap transgenic complementation in maize. Front. Plant Sci. **7**:414. https://doi.org/10.3389/fpls.2016.00414.

Kelliher, T., Starr, D., Richbourg, L., Chintamanani, S., Delzer, B., Nuccio, M.L., Green, J., Chen, Z., McCuiston, J., Wang, W., et al. (2017). MATRILINEAL, a sperm-specific phospholipase, triggers maize haploid induction. Nature **542**:105–109. https://doi.org/10.1038/nature20827.

Kuo, Y.T., Câmara, A.S., Schubert, V., Neumann, P., Macas, J., Melzer, M., Chen, J., Fuchs, J., Abel, S., Klocke, E., et al. (2023). Holocentromeres can consist of merely a few megabase-sized satellite arrays. Nat. Commun. **14**:3502. https://doi.org/10.1038/s41467-023-38922-7.

Kuppu, S., Tan, E.H., Nguyen, H., et al. (2015). Point mutations in centromeric histone induce post-zygotic incompatibility and uniparental inheritance. PLoS Genet. **11**:e1005494. https://doi.org/10.1371/journal.pgen.1005494.

Kuppu, S., Ron, M., Marimuthu, M.P.A., Li, G., Huddleson, A., Siddeek, M.H., Terry, J., Buchner, R., Shabek, N., Comai, L., and Britt, A.B. (2020). A variety of changes, including CRISPR/Cas9-mediated deletions, in CENH3 lead to haploid induction on outcrossing. Plant Biotechnol. J. **18**:2068–2080. https://doi.org/10.1111/pbi.13365.

Le Goff, S., Keçeli, B.N., Jeřábková, H., Heckmann, S., Rutten, T., Cotterell, S., Schubert, V., Roitinger, E., Mechtler, K., Franklin, F.C.H., et al. (2020). The H3 histone chaperone NASP(SIM3) escorts CenH3 in Arabidopsis. Plant J. **101**:71–86. https://doi.org/10.1111/tpj.14518.

Lermontova, I., Rutten, T., and Schubert, I. (2011a). Deposition, turnover, and release of CENH3 at Arabidopsis centromeres. Chromosoma **120**:633–640. https://doi.org/10.1007/s00412-011-0338-5.

Lermontova, I., Koroleva, O., Rutten, T., Fuchs, J., Schubert, V., Moraes, I., Koszegi, D., and Schubert, I. (2011b). Knockdown of CENH3 in Arabidopsis reduces mitotic divisions and causes sterility by disturbed meiotic chromosome segregation. Plant J. **68**:40–50. https://doi.org/10.1111/j.1365-313X.2011.04664.x.

Lv, J., Yu, K., Wei, J., Gui, H., Liu, C., Liang, D., Wang, Y., Zhou, H., Carlin, R., Rich, R., et al. (2020). Generation of paternal haploids in wheat by genome editing of the centromeric histone CENH3. Nat. Biotechnol. **38**:1397–1401. https://doi.org/10.1038/s41587-020-0728-4.

Maheshwari, S., Tan, E.H., West, A., Franklin, F.C.H., Comai, L., and Chan, S.W.L. (2015). Naturally occurring differences in CENH3 affect

chromosome segregation in zygotic mitosis of hybrids. PLoS Genet. **11**:e1004970. https://doi.org/10.1371/journal.pgen.1004970.

Manape, T.K., Satheesh, V., Somasundaram, S., et al. (2024). RNAi-mediated downregulation of AcCENH3 can induce in vivo haploids in onion (*Allium cepa* L.). Sci. Rep. **14**:14481. https://doi.org/10.1038/s41598-024-64432-7.

Marimuthu, M.P.A., Maruthachalam, R., Bondada, R., Kuppu, S., Tan, E.H., Britt, A., Chan, S.W.L., and Comai, L. (2021). Epigenetically mismatched parental centromeres trigger genome elimination in hybrids. Sci. Adv. **7**:eabk1151. https://doi.org/10.1126/sciadv.abk1151.

Merker, L., Schindele, P., Huang, T.K., Wolter, F., and Puchta, H. (2020). Enhancing in planta gene targeting efficiencies in Arabidopsis using temperature-tolerant CRISPR/LbCas12a. Plant Biotechnol. J. **18**:2382–2384. https://doi.org/10.1111/pbi.13426.

Murashige, T., and Skoog, F. (2006). A revised medium for rapid growth and bio assays with tobacco tissue cultures. Physiol. Plantarum **15**:473–497. https://doi.org/10.1111/j.1399-3054.1962.tb08052.x.

Niikura, Y., Kitagawa, R., Fang, L., and Kitagawa, K. (2019). CENP-A ubiquitylation is indispensable to cell viability. Dev. Cell **50**:683–689.e6. https://doi.org/10.1016/j.devcel.2019.07.015.

Park, S.K., Howden, R., and Twell, D. (1998). The *Arabidopsis thaliana* gametophytic mutation gemini pollen1 disrupts microspore polarity, division asymmetry and pollen cell fate. Development **125**:3789–3799. https://doi.org/10.1242/dev.125.19.3789.

Paul, W., Hodge, R., Smartt, S., Draper, J., and Scott, R. (1992). The isolation and characterisation of the tapetum-specific *Arabidopsis thaliana* A9 gene. Plant Mol. Biol. **19**:611–622. https://doi.org/10.1007/BF00026787.

Phan, H.T., and Conrad, U. (2016). Plant-based vaccine antigen production. In Vaccine Technologies for Veterinary Viral Diseases: Methods and Protocols, A. Brun, ed. (Springer), pp. 35–47.

Quiroz, L.F., Gondalia, N., Brychkova, G., McKeown, P.C., and Spillane, C. (2024). Haploid rhapsody: the molecular and cellular orchestra of *in vivo* haploid induction in plants. New Phytol. **241**:1936–1949. https://doi.org/10.1111/nph.19523.

Ravi, M., and Chan, S.W.L. (2010). Haploid plants produced by centromere-mediated genome elimination. Nature **464**:615–618. https://doi.org/10.1038/nature08842.

Ravi, M., Kwong, P.N., Menorca, R.M.G., et al. (2010). The rapidly evolving centromere-specific histone has stringent functional requirements in *Arabidopsis thaliana*. Genetics **186**:461–471. https://doi.org/10.1534/genetics.110.120337.

Ravi, M., Marimuthu, M.P.A., Tan, E.H., et al. (2014). A haploid genetics toolbox for *Arabidopsis thaliana*. Nat. Commun. **5**:5334. https://doi.org/10.1038/ncomms6334.

Raychaudhuri, N., Dubruille, R., Orsi, G.A., Bagheri, H.C., Loppin, B., and Lehner, C.F. (2012). Transgenerational propagation and quantitative maintenance of paternal centromeres depends on Cid/Cenp-A presence in Drosophila sperm. PLoS Biol. **10**:e1001434. https://doi.org/10.1371/journal.pbio.1001434.

Rönspies, M., Schindele, P., Wetzel, R., and Puchta, H. (2022). CRISPR-Cas9-mediated chromosome engineering in *Arabidopsis thaliana*. Nat. Protoc. **17**:1332–1358. https://doi.org/10.1038/s41596-022-00686-7.

Rothbauer, U., Zolghadr, K., Tillib, S., Nowak, D., Schermelleh, L., Gahl, A., Backmann, N., Conrath, K., Muyldermans, S., Cardoso, M.C., and Leonhardt, H. (2006). Targeting and tracing antigens in live cells with fluorescent nanobodies. Nat. Methods **3**:887–889. https://doi.org/10.1038/nmeth953.

Sanei, M., Pickering, R., Kumke, K., Nasuda, S., and Houben, A. (2011). Loss of centromeric histone H3 (CENH3) from centromeres precedes uniparental chromosome elimination in interspecific barley hybrids. Proc. Natl. Acad. Sci. USA **108**:E498–E505. https://doi.org/10.1073/pnas.1103190108.

Schindele, P., Merker, L., Schreiber, T., Prange, A., Tissier, A., and Puchta, H. (2023). Enhancing gene editing and gene targeting efficiencies in *Arabidopsis thaliana* by using an intron-containing version of ttLbCas12a. Plant Biotechnol. J. **21**:457–459. https://doi.org/10.1111/pbi.13964.

Shimada, T.L., Shimada, T., and Hara-Nishimura, I. (2010). A rapid and non-destructive screenable marker, FAST, for identifying transformed seeds of *Arabidopsis thaliana*. Plant J. **61**:519–528. https://doi.org/10.1111/j.1365-313X.2009.04060.x.

Shin, Y.J., Park, S.K., Jung, Y.J., Kim, Y.N., Kim, K.S., Park, O.K., Kwon, S.H., Jeon, S.H., Trinh, L.A., Fraser, S.E., et al. (2015). Nanobody-targeted E3-ubiquitin ligase complex degrades nuclear proteins. Sci. Rep. **5**:14269. https://doi.org/10.1038/srep14269.

Sorge, E., Demidov, D., Lermontova, I., Houben, A., and Conrad, U. (2021). Engineered degradation of EYFP-tagged CENH3 via the 26S proteasome pathway in plants. PLoS One **16**:e0247015. https://doi.org/10.1371/journal.pone.0247015.

Sprunck, S., Rademacher, S., Vogler, F., Gheyselinck, J., Grossniklaus, U., and Dresselhaus, T. (2012). Egg cell-secreted EC1 triggers sperm cell activation during double fertilization. Science **338**:1093–1097. https://doi.org/10.1126/science.1223944.

Takai, R., Matsuda, N., Nakano, A., Hasegawa, K., Akimoto, C., Shibuya, N., and Minami, E. (2002). EL5, a rice N-acetylchitooligosaccharide elicitor-responsive RING-H2 finger protein, is a ubiquitin ligase which functions in vitro in co-operation with an elicitor-responsive ubiquitin-conjugating enzyme, OsUBC5b. Plant J. **30**:447–455. https://doi.org/10.1046/j.1365-313x.2002.01299.x.

Wang, G., Zong, M., Han, S., et al. (2024). *In vivo* haploid induction in cauliflower, kale, and broccoli. J. Integr. Plant Biol. **66**:1823–1826. https://doi.org/10.1111/jipb.13730.

Wang, N., Gent, J.I., and Dawe, R.K. (2021). Haploid induction by a maize cenh3 null mutant. Sci. Adv. **7**:eabe2299. https://doi.org/10.1126/sciadv.abe2299.

Wang, Z., Chen, M., Yang, H., Hu, Z., Yu, Y., Xu, H., Yan, S., Yi, K., and Li, J. (2023). A simple and highly efficient strategy to induce both paternal and maternal haploids through temperature manipulation. Nat. Plants **9**:699–705. https://doi.org/10.1038/s41477-023-01389-x.

Weale, A. (2010). Ethical arguments relevant to the use of GM crops. N. Biotechnol. **27**:582–587. https://doi.org/10.1016/j.nbt.2010.08.013.

Widiez, T. (2021). Haploid embryos: being like mommy or like daddy? Trends Plant Sci. **26**:425–427. https://doi.org/10.1016/j.tplants.2021.02.010.

Wu, F.H., Shen, S.C., Lee, L.Y., Lee, S.H., Chan, M.T., and Lin, C.S. (2009). Tape-Arabidopsis Sandwich - a simpler Arabidopsis protoplast isolation method. Plant Methods **5**:16. https://doi.org/10.1186/1746-4811-5-16.

Yang, H., Zhang, R., Wu, S., et al. (2025). Efficient haploid induction in *Arabidopsis thaliana* by fine-tuning CENH3 function. Plant Commun. **6**:101349. https://doi.org/10.1016/j.xplc.2025.101349.

Zhong, Y., Chen, B., Li, M., et al. (2020). A DMP-triggered *in vivo* maternal haploid induction system in the dicotyledonous Arabidopsis. Nat. Plants **6**:466–472. https://doi.org/10.1038/s41477-020-0658-7.

Zhong, Y., Liu, C., Qi, X., Jiao, Y., Wang, D., Wang, Y., Liu, Z., Chen, C., Chen, B., Tian, X., et al. (2019). Mutation of ZmDMP enhances haploid induction in maize. Nat. Plants **5**:575–580. https://doi.org/10.1038/s41477-019-0443-7.

Plant Communications, Volume 7

Supplemental information

Targeted CENH3 protein depletion in egg cells enables highly efficient haploid induction

Saravanakumar Somasundaram, Seda Yaşar, Jörg Fuchs, Maria Cuacos, Julian Claassen, Oda Weiss, Andriy Kochevenko, Jonathan C. Lamb, Tengyu Li, Niklas Capdeville, Holger Puchta, and Andreas Houben

Supplemental figures:

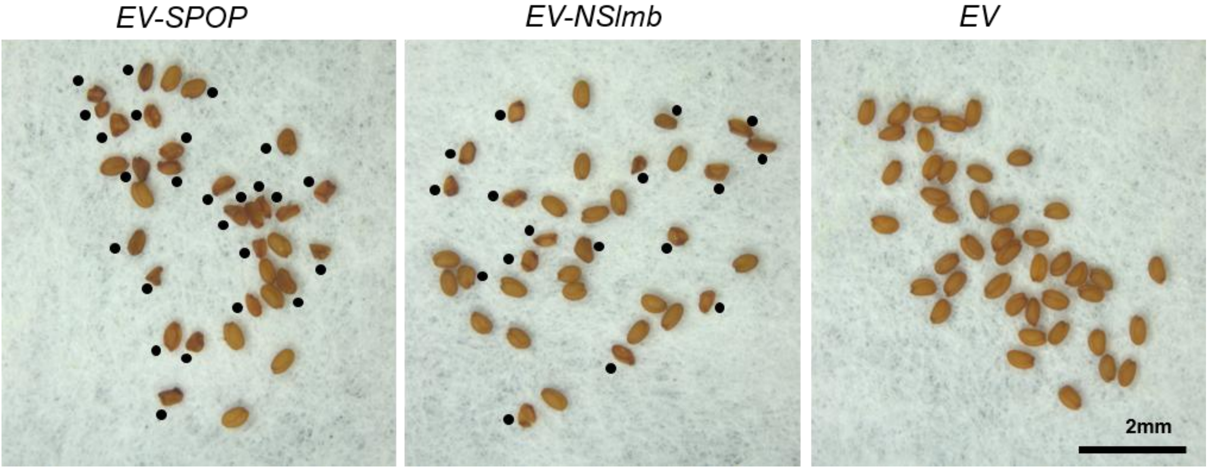

Figure S1: Egg cell-specific degradation of EYFP-CENH3 results in shriveled seeds after self-pollination. Images of seeds from a representative silique of representative *EV-SPOP, EV-NSlmb and EV T1* plants in the genetic background of *EYFP-gCENH3*$^{cenh3\text{-}1}$. Black dots indicate the shriveled seeds.

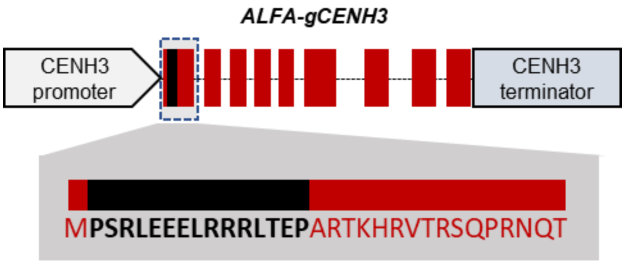

Figure S2: Schematic representation of the *ALFA-gCENH3* construct showing the site of the ALFA-tag insertion. The first exon is zoomed in, and the amino acids highlighted in black corresponds to the ALFA-tag.

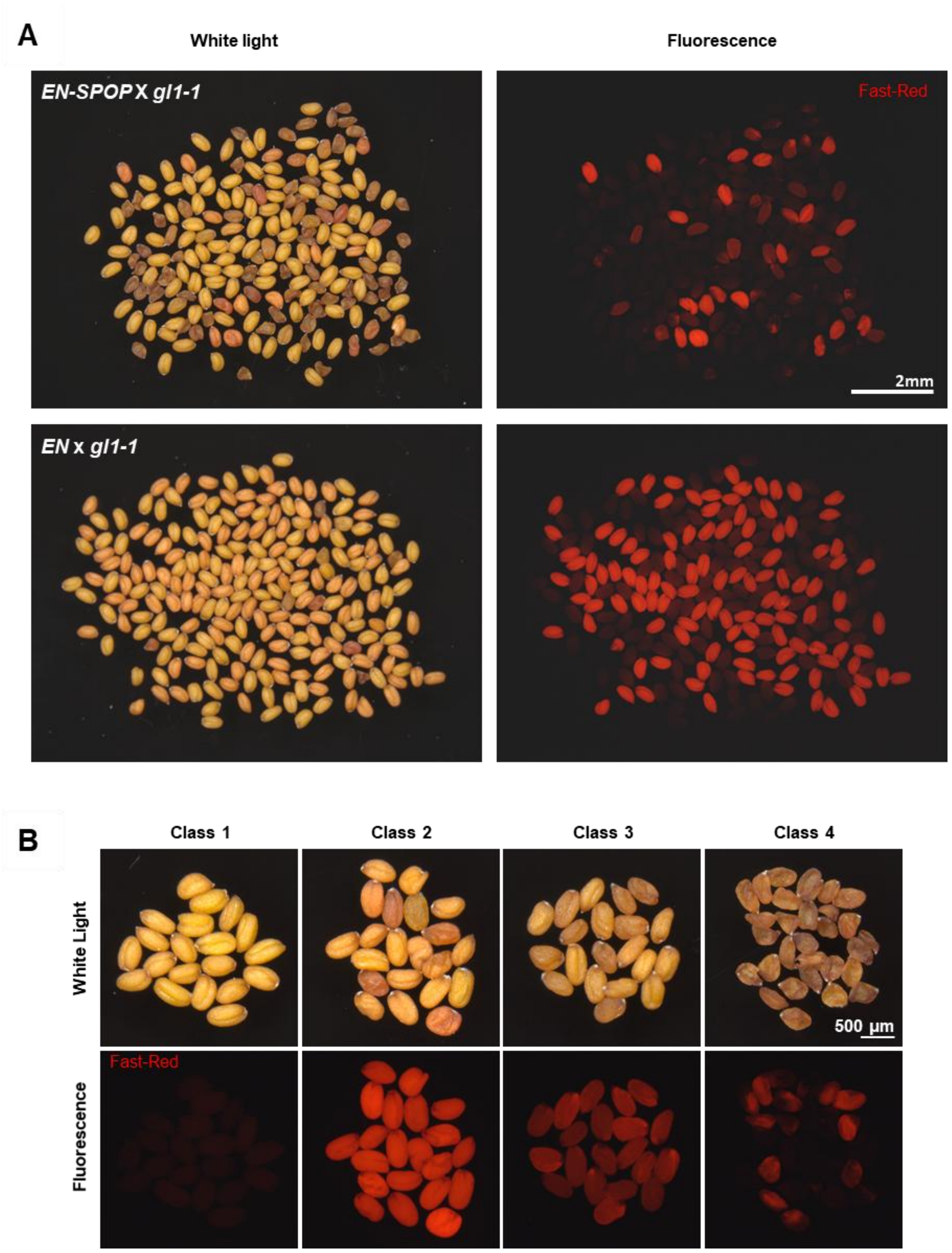

Figure S3: Comparison of F1 seed phenotypes from *ALFA-gCENH3*$^{cenh3\text{-}1}$ mothers with and without engineered E3 ligases. A) Seeds were obtained from crosses between *ALFA-gCENH3*$^{cenh3\text{-}1}$ mothers carrying either *EN-SPOP* (with engineered E3 ligases) and *EN* (control without E3 ligases) transgene and *gl1-1* fathers. Crosses with *EN* mothers produced

predominantly viable F1 seeds with a high proportion exhibiting fluorescence. In contrast, crosses involving *EN-SPOP* mothers resulted in increased seed lethality and a substantially reduced proportion of fluorescent seeds. Fluorescent seeds could be further classified based on fluorescence intensity differences. **B)** Different seed classes observed among the F1 seeds from EN-SPOP T1 females.

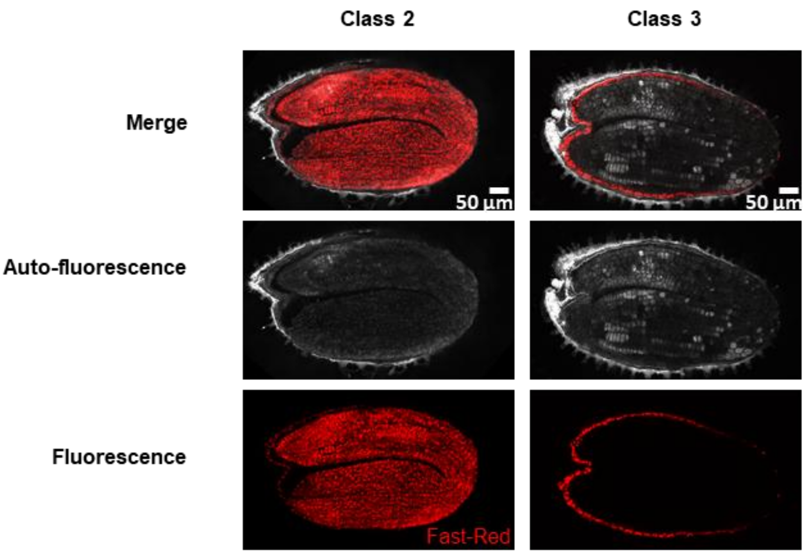

Figure S4: Differential fluorescence distribution in Class 2 and Class 3 seeds. In Class 2 seeds, fluorescence is detected in both the embryo and endosperm. In contrast, Class 3 seeds, which primarily give rise to haploids, exhibit fluorescence only in the endosperm, with no signal observed in the embryo.

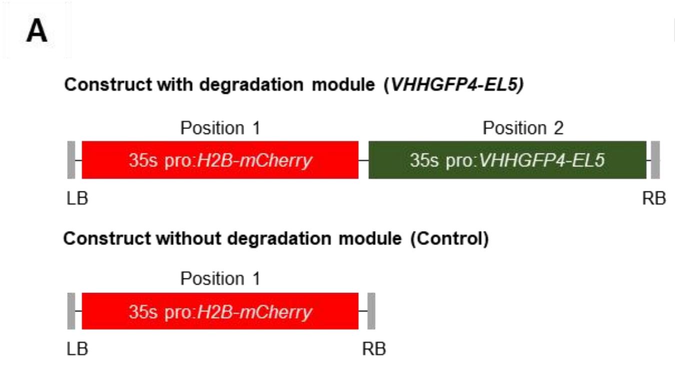

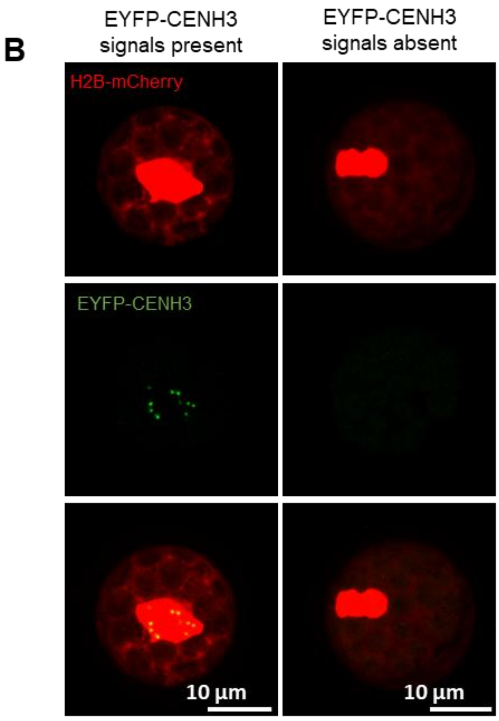

C

Construct	n	Protoplasts with EYFP-CENH3 signal (%)	Protoplasts without EYFP-CENH3 signal (%)
VHHGFP4-EL5	53	3.77	96.23
Control	56	82.14	17.86

Figure S5: Assessment of the functionality of the E3 ligase, EL5 for CENH3 degradation: A) Schematic representation of transgene cassettes with and without the degradation module (VHHGFP4-EL5) used for protoplast transformation. Protoplasts were derived from *EYFP-gCENH3*$^{cenh3\text{-}1}$ plants. **B)** Representative protoplasts showing nuclei labelled with histone H2B-mCherry (red) and displaying either presence or absence of EYFP-CENH3. **C).** Proportion of protoplasts with and without EYFP-CENH3 signals following transformation with the constructs shown in panel (A). ‘n’ indicates the total number of transformed protoplasts analysed for each construct.

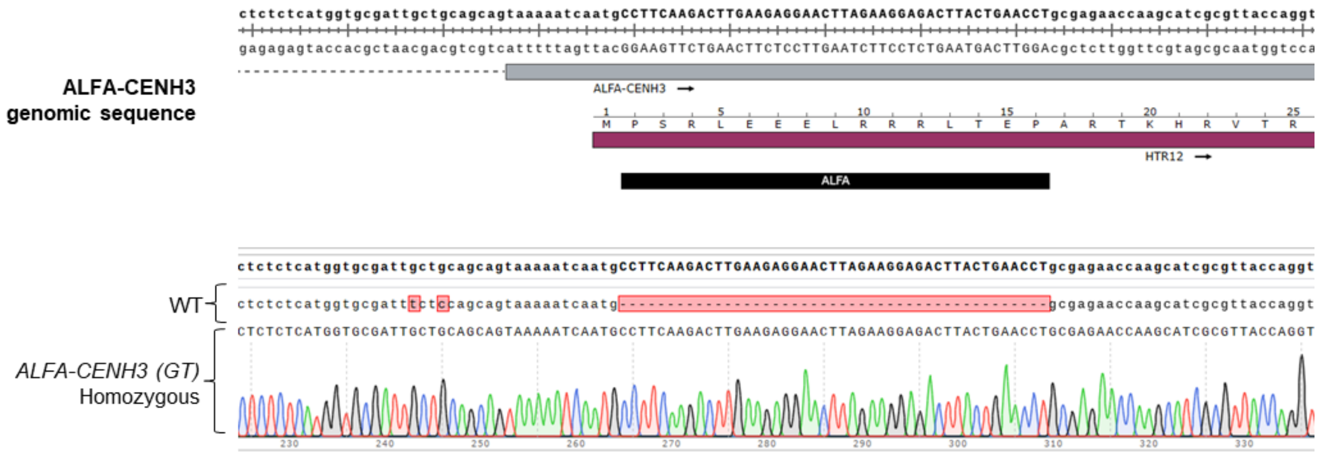

Figure S6: Sanger sequencing chromatogram of a representative *ALFA-CENH3 (GT)* homozygous plant. The sequence is compared with the WT sequence, and the sites where silent mutations were introduced are highlighted.

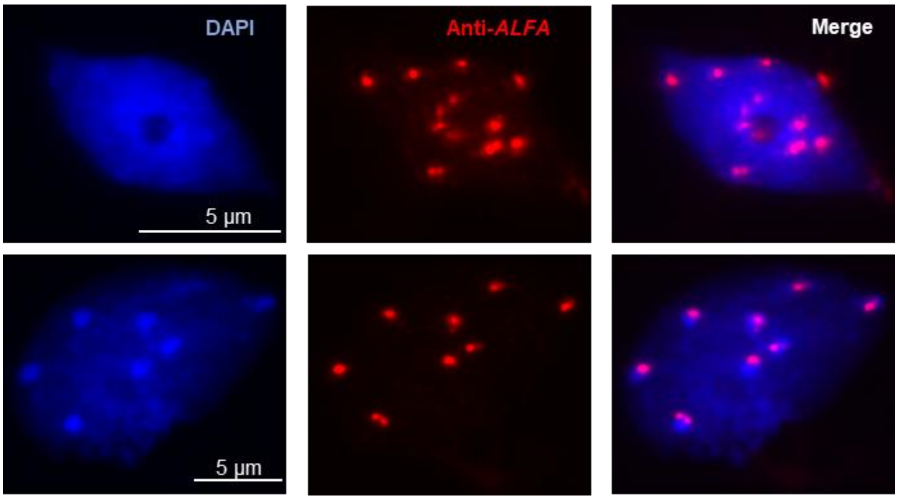

Figure S7: Immunolabeling of leaf nuclei from *ALFA-CENH3 (GT)* homozygous plant with anti-ALFA antibody.

Supplemental Tables

Table S1: ***EYFP-gCENH3$^{cenh3\text{-}1}$*** **results in variable frequencies of haploids on outcrossing.** Haploids are determined based on glabrous seedlings among the progenies observed from crosses with *gl1-1* fathers.

Replicates	EYFP-gCENH3$^{cenh3\text{-}1}$ @			EYFP-gCENH3$^{cenh3\text{-}1}$ #		
	**Total**	**Haploids**	**(%)**	**Total**	**Haploids**	**(%)**
1	48	6	12.50	27	4	14.81
2	271	15	5.54	17	2	11.76
3	62	12	19.35	46	6	13.04
4	59	12	20.34	26	3	11.54
5	68	8	11.76	41	5	12.20
6	184	15	8.15	89	6	6.74
7	29	4	13.79	32	4	12.50
8	41	4	9.76	16	1	6.25
9	19	2	10.53	74	6	8.11
10	32	4	12.50	31	3	9.68

@ - line used in the previous study (Demidov et al. 2022). # - line from an independent complementation experiment.

Table S2: Proportion of F1 seed classes from *ALFA-gCENH3*$^{cenh3-1}$ mother plants with and without engineered E3 ligases, and their corresponding germinated and glabrous seedlings. F1 seeds from three independent T1 mother plants were analyzed for each construct: *EN-SPOP* (with engineered E3 ligases) and *EN* (control without E3 ligases). Seeds were classified based on fluorescence intensity and morphological characteristics as described in Fig. 5. Numbers in parentheses indicate the proportion of seeds in each category relative to total seeds analyzed.

Cross	T1 replicate	Total number of seeds analysed	No. of seeds (%)				Germinated seedlings				Glabrous seedlings			
			Class 1	Class 2	Class 3	Class 4	Class 1	Class 2	Class 3	Class 4	Class 1	Class 2	Class 3	Class 4
EN-SPOP x *gl1-1*	1	604	275 (45.5 %)	75 (12.4 %)	44 (7.3 %)	210 (34.8 %)	251	13	6	5	0	0	6	0
	2	1206	468 (38.8 %)	78 (6.5 %)	76 (6.3 %)	584 (48.4 %)	420	10	17	1	0	0	17	0
	3	1032	465 (45.1 %)	108 (10.5 %)	68 (6.6 %)	391 (37.9 %)	429	13	15	8	0	0	13	0
EN x *gl1-1*	1	641	156 (24.3 %)	460 (71.8 %)	0	25 (3.9 %)	141	395	0	2	0	0	0	0
	2	799	355 (44.4 %)	416 (52.1 %)	0	28 (3.5 %)	326	391	0	3	0	0	0	0
	3	679	296 (43.6 %)	314 (46.2 %)	0	69 (10.2 %)	258	272	0	0	0	0	0	0

Table S3: Ploidy analysis of different F1 seed classes from *EN-SPOP* plants. Flow cytometry analysis was performed on 'n' seeds per class from *EN-SPOP* T1 mother plants. Diploid Col-0 seeds served as internal reference for classes 1-3. Class 4 seeds were measured without an internal reference due to low number of isolatable nuclei in this seed class.

Cross	Seed category	n	Haploid (%)	Diploid (%)	Aneuploid (%)	Mixoploid (%)
EN-SPOP x *gl1-1*	Class 1	20	0	100	0	0
	Class 2	40	0	72.5	25	2.5
	Class 3	40	72.5	15	7.5	5
	Class 4	20	0	0	0	0

Table S4: Accuracy of FAST-Red seed marker and glabrous phenotype for haploid progeny identification. The accuracy of FAST-Red seed marker was calculated as the proportion of flow cytometry-confirmed haploids among germinated seedlings derived from seed class 3 exhibiting faint FAST-Red fluorescence. Accuracy of glabrous phenotype was calculated as the proportion of flow cytometry-confirmed haploids among glarous seedlings.

Cross	T1 replicate	Seedlings from class 3 seeds	Total glabrous seedlings	Haploids based on flow cytometry	Accuracy of glabrous phenotype (%)	Accuracy of class 3 seed phenotype (%)
EN-SPOP x *gl1-1*	1	6	6	6	100.00	100.00
	2	17	17	16	94.12	94.12
	3	15	13	13	100.00	86.67
	**Total**	**38**	**36**	**35**	**97.22**	**92.11**

Table S5: Primer sequences used in this study.

**Purpose**	**Primer name**	**Sequence**
EcoRV dCAPS for genotyping *cenh3-1* (in case of *EYFP-gCENH3* complementation)	CenH3-dCAPS-F	GGTGCGATTTCTCCAGCAGTAAAAATC
	CenH3-dCAPS-R	CTGAGAAGATGAAGCACCGGCGATAT
EcoRV dCAPS for genotyping cenh3-1 (in case of *ALFA-gCENH3* complementation)	AtCENH3-Geno-F2	CTCCAGCAGTAAAAATCAATGGATAGA
	CenH3-dCAPS-R	CTGAGAAGATGAAGCACCGGCGATAT
Primers used for generating PCR fragments to assemble ALFA-gCENH3	CENH3-Bsa1-F1	TTGGTCTCATCCCGTACGGACGCATCATC AACATCTG
	CENH3-Bsa1-R1	TTGGTCTCATTCCTCTTCCAGCCTGGACG GCATTGATTTTTACTGCTGGAGAAATC
	CENH3-Bsa1-F2	TTGGTCTCAGGAACTCAGACGTAGGCTCA CGGAACCTGCGAGAACCAAGCATCGCG
	CENH3-Bsa1-R2	TTGGTCTCACAGATTTCGTTTAAGGACCA ATGAG
Gene-targeting for *in-locus* ALFA tagging of CENH3	NC368	CTATCCTCGGTAATGACCAG
	NC369	CCTGTCTGGATCTCTTAGCC
	NC370	CTAACAATCGAGTCGAACCC
	SY209	CCTCTTCAAGTCTTGAAGG

Table S6: Level-0 modules used in this study and their Addgene ID number.

Module Name	Destination	Addgene ID
NOS-PPT	pICH41331	232550
CAMV35S-promoter	pICH41295	232551
EC1.1-promoter	pICH41295	232552
A9-promoter	pICH41295	232553
HSP-terminator	pAGM9121	232554
RbcSE9-tererminator	pAGM9121	232555
Barnase	pAGM1287	232556
H2B	pICH41258	232557
mCherry	pAGM1299	232558
VHHGFP4	pAGM1287	232559
VHHGFP4-SPOP	pAGM1287	232560
Nslmb-VHHGFP4	pAGM1287	232561
NbALFA-N	pICH41258	232562
NbALFA-C	pAGM1299	232563
NbALFA	pAGM1287	232564
Nslmb	pICH41258	232565
SPOP	pAGM1299	232566
EYFP	pICH41258	232567
CENH3-cds	pAGM9121	232568
EL5	pAGM1299	232569
VHHGFP4-N	pICH41258	232571
VHHGFP4-C	pAGM1299	232572
ALFA-gCENH3	pICH86966	232575

Table S7: Level-1 modules used in this study: List of level-0 modules and destination plasmid used for the construction of level-1 plasmids.

Construct	Destination	Level-0 modules			
		1	2	3	4
PPT	pICH47802	NOS-PPT	-	-	-
Kan	pICH47802	NOS-Kan			
EVS	pICH47811	EC1.1-pro	VHHGFP4-SPOP	RbcSE9-terminator	-
ENV	pICH47811	EC1.1-promoter	Nslmb-VHHGFP4	RbcSE9-terminator	-
EV	pICH47811	EC1.1-promoter	VHHGFP4	RbcSE9-terminator	-
ENS	pICH47811	EC1.1-promoter	NbALFA-N	SPOP	RbcSE9-terminator
ENN	pICH47811	EC1.1-promoter	Nslmb	NbALFA-C	RbcSE9-terminator
EN	pICH47811	EC1.1-promoter	NbALFA	RbcSE9-terminator	-
ENE	pICH47811	EC1.1-promoter	NbALFA-N	EL5	RbcSE9-terminator
EVE	pICH47811	EC1.1-promoter	VHHGFP4-N	EL5	RbcSE9-terminator
FAST	pICH47822	pICSL70008 (Addgene #50336)	-	-	-
Barnase	pICH47822/ pICH47831	A9-promoter	Barnase	HSP-terminator	
H2B-mCherry	pICH47732	CAMV35S-promoter	H2B	mCherry	HSP-terminator
Degradation module (VHHGFP4-EL5)	pICH47742	CAMV35S-promoter	VHHGFP4-N	EL5	RbcSE9-terminator
EYFP-*Sl*CENH3	pICH47751	CAMV35S-promoter	EYFP	*Sl*CENH3 (Synthetic fragment)	RbcSE9-terminator

Table S8: Level-2 modules used in this study: List of level-1 modules used for the construction of level-2 plasmids.

Construct	Destination	Position 1	Position 2	Position 3	Position 4
EV-SPOP	pICSL4723	PPT	EVS	-	-
EV-NSlmb	pICSL4723	PPT	ENV	-	-
EV	pICSL4723	PPT	EV	-	-
EN-SPOP	pICSL4723	PPT	ENS	FAST	Barnase
EN-SPOP (for transformation of *ALFA-CENH3* (GT) plants)	pICSL4723	Kan	ENS	FAST	Barnase
EN-NSlmb	pICSL4723	PPT	ENN	FAST	Barnase
EN	pICSL4723	PPT	EN	FAST	Barnase
Control (only Barnase) for *ALFA-gCENH3*	pICSL4723	PPT	-	FAST	Barnase
EN-EL5	pICSL4723	PPT	ENE	FAST	Barnase
EV-EL5	pICSL4723	PPT	EVE	Barnase	
Control (only Barnase) for *EYFP-gCENH3*	pICSL4723	PPT	-	Barnase	
Construct with degradation Module (VHHGFP4-EL5)	pICSL4723	H2B-mCherry	VHHGFP4-EL5		
Control Construct without degradation Module	pICSL4723	H2B-mCherry	-		
Construct with degradation Module (VHHGFP4-EL5) for tomato CENH3 experiment	pICSL4723	H2B-mCherry	VHHGFP4-EL5	EYFP-*Sl*CENH3	
Control Construct without degradation Module for tomato CENH3 experiment	pICSL4723	H2B-mCherry	-	EYFP-*Sl*CENH3	

Table S9: Haploid induction frequency of individual crosses presented in Figure 2D.

Construct	Replicate	No. of progenies screened	No. of Haploid (glabrous) plants	Haploid frequency (%)	Median (%)
EV-SPOP	1	87	26	29.89	25.17
	2	94	20	21.28	
	3	19	11	57.89	
	4	24	4	16.67	
	5	142	37	26.06	
	6	138	24	17.39	
	7	131	49	37.40	
	8	173	42	24.28	
	9	79	23	29.11	
	10	60	17	28.33	
	11	92	24	26.09	
	12	38	8	21.05	
	13	153	29	18.95	
	14	79	18	22.78	
EV-NSlmb	1	126	28	22.22	22.315
	2	79	28	35.44	
	3	241	54	22.41	
	4	323	58	17.96	
	5	186	74	39.78	
	6	514	89	17.32	
	7	65	10	15.38	
	8	116	40	34.48	
	9	245	41	16.73	
	10	305	41	13.44	
	11	100	27	27.00	
	12	117	32	27.35	
	13	171	44	25.73	
	14	333	52	15.62	
	15	83	14	16.87	
	16	247	81	32.79	
EV	1	357	60	16.81	23.08
	2	191	34	17.80	
	3	134	30	22.39	
	4	276	69	25.00	
	5	107	25	23.36	
	6	149	39	26.17	
	7	118	32	27.12	
	8	79	26	32.91	
	9	337	64	18.99	
	10	195	45	23.08	
	11	69	17	24.64	
	12	181	41	22.65	
	13	24	5	20.83	
Control	1	46	5	10.87	17.105
	2	38	5	13.16	
	3	97	11	11.34	
	4	76	18	23.68	
	5	109	23	21.10	
	6	39	9	23.08	

	7	118	7	5.93	
	8	169	41	24.26	
	9	40	5	12.50	
	10	133	28	21.05	

Table S10: Haploid induction frequency of individual crosses presented in Figure 3B.

T1_Family	Replicate	No. of progenies screened	No. of Haploid (glabrous) plants	Haploid frequency (%)
34-1	1	298	13	4.36
	2	237	5	2.11
	3	192	10	5.21
	4	218	3	1.38
	5	220	5	2.27
	6	200	10	5.00
	7	143	3	2.10
	8	200	11	5.50
	9	244	9	3.69
	10	237	5	2.11
34-2	1	56	3	5.36
	2	46	2	4.35
	3	66	5	7.58
	4	267	0	0.00
	5	20	3	15.00
	6	454	0	0.00
	7	87	5	5.75
	8	434	0	0.00
	9	54	3	5.56
	10	254	0	0.00
34-8	1	44	3	6.82
	2	128	5	3.91
	3	140	4	2.86
	4	50	2	4.00
	5	36	2	5.56
	6	55	3	5.45
	7	90	1	1.11
	8	183	3	1.64
	9	87	2	2.30
	10	56	2	3.57
34-11	1	88	13	14.77
	2	82	4	4.88
	3	108	13	12.04
	4	90	5	5.56
	5	89	10	11.24
	6	66	10	15.15
	7	106	8	7.55
	8	89	8	8.99
	9	107	6	5.61
	10	111	12	10.81
34-15	1	108	2	1.85
	2	219	11	5.02
	3	124	5	4.03
	4	443	5	1.13
	5	230	3	1.30
	6	421	1	0.24
	7	302	5	1.66
	8	303	16	5.28
	9	435	1	0.23

	10	431	1	0.23
34-20	1	124	4	3.23
	2	80	4	5.00
	3	136	11	8.09
	4	92	3	3.26
	5	153	8	5.23
	6	122	6	4.92
	7	98	5	5.10
	8	54	3	5.56
	9	91	6	6.59
	10	54	6	11.11

Table S11: Haploid induction frequency of individual crosses presented in Figure 3D.

Construct	Replicate	No. of progenies screened	No. of Haploid (glabrous) plants	Haploid frequency (%)	Median (%)
EN-SPOP	1	342	10	2.92	4.11
	2	324	18	5.56	
	3	76	7	9.21	
	4	73	3	4.11	
	5	307	10	3.26	
	6	280	8	2.86	
	7	93	12	12.90	
	8	350	13	3.71	
	9	230	3	1.30	
	10	169	8	4.73	
	11	167	10	5.99	
EN-NSlmb	1	172	7	4.07	1.31
	2	342	3	0.88	
	3	322	2	0.62	
	4	185	1	0.54	
	5	290	3	1.03	
	6	287	8	2.79	
	7	228	4	1.75	
	8	301	0	0.00	
	9	252	4	1.59	
	10	71	2	2.82	
EN	1	526	0	0.00	0.00
	2	260	0	0.00	
	3	384	0	0.00	
	4	312	2	0.64	
	5	428	0	0.00	
	6	249	0	0.00	
	7	248	0	0.00	
	8	388	0	0.00	
	9	380	0	0.00	
	10	284	0	0.00	
Control	1	530	0	0.00	0.00
	2	245	0	0.00	
	3	421	0	0.00	
	4	398	0	0.00	
	5	487	0	0.00	
	6	268	0	0.00	
	7	354	0	0.00	
	8	289	0	0.00	
	9	189	0	0.00	
	10	464	0	0.00	

Table S12: Haploid induction frequency of individual crosses presented in Figure 4C.

Construct	Replicate	No. of progenies screened	No. of Haploid (glabrous) plants	Haploid frequency (%)	Median (%)
Control	1	384	0	0.00	0.00
	2	591	0	0.00	
	3	458	0	0.00	
	4	393	0	0.00	
	5	534	0	0.00	
	6	351	0	0.00	
	7	298	0	0.00	
	8	421	0	0.00	
	9	398	0	0.00	
	10	448	0	0.00	
EN-EL5	1	281	5	1.78	1.76
	2	287	5	1.74	
	3	243	7	2.88	
	4	215	2	0.93	
	5	185	7	3.78	
	6	213	2	0.94	
	7	239	20	8.37	
	8	155	4	2.58	
	9	227	3	1.32	
	10	181	2	1.10	

Table S13: Haploid induction frequency of individual crosses presented in Figure 4D.

Construct	Replicate	No. of progenies screened	No. of Haploid (glaborous) plants	Haploid frequency (%)	Median (%)
Control	1	28	4	14.29	17.50
	2	30	7	23.33	
	3	38	11	28.95	
	4	26	5	19.23	
	5	28	3	10.71	
	6	34	5	14.71	
	7	36	4	11.11	
	8	31	6	19.35	
	9	40	7	17.50	
	10	35	4	11.43	
	11	33	6	18.18	
	12	16	2	12.50	
	13	23	5	21.74	
	14	88	9	10.23	
	15	30	6	20.00	
EV-EL5	1	25	7	28.00	51.39
	2	7	4	57.14	
	3	6	3	50.00	
	4	6	4	66.67	
	5	9	3	33.33	
	6	20	11	55.00	
	7	10	4	40.00	
	8	20	7	35.00	
	9	36	19	52.78	
	10	24	16	66.67	
	11	12	6	50.00	
	12	18	6	33.33	
	13	27	16	59.26	
	14	16	9	56.25	
	15	16	5	31.25	
	16	33	21	63.64	
	17	34	21	61.76	
	18	25	9	36.00	
	19	25	18	72.00	
	20	28	8	28.57	
	21	36	15	41.67	
	22	21	13	61.90	
	23	44	26	59.09	
	24	23	11	47.83	

Table S14: Haploid induction frequency of individual crosses presented in Figure 5C.

Construct	Replicate	No. of progenies screened	No. of Haploid (glaborous) plants	Haploid frequency (%)	Median (%)
Control	1	136	0	0.00	0.00
	2	154	0	0.00	
	3	195	0	0.00	
	4	210	0	0.00	
	5	145	0	0.00	
	6	223	0	0.00	
	7	123	0	0.00	
	8	129	0	0.00	
	9	232	0	0.00	
	10	113	0	0.00	
EN-SPOP	1	277	32	11.55	16.38
	2	223	43	19.28	
	3	110	17	15.45	
	4	124	25	20.16	
	5	177	31	17.51	
	6	232	38	16.38	
	7	230	45	19.57	
	8	220	23	10.45	
	9	192	26	13.54	
	10	154	28	18.18	
	11	124	13	10.48	

Supplemental Data

Data S1: Sequence information relevant to in-locus ALFA tagging of CENH3

>CENH3 Cas12a cleavage site

TCCAGCAGTAAAAATCAATGGCGA

>*At*CENH3 GT donor – pUC57 (SpeI flanked)

The sequence organization is as follows: SpeI site – CRISPR target/PAM – 5′ homology arm including two silent point mutations (in red) – ALFA-tag CDS – 3′ homology arm – CRISPR target/PAM – SpeI site.

actagttttctccagcagtaaaaatcaatggcgaAACCTATGATTGGATGCTGAGAACTTGTAAAGAATCTGAGGC
AGAAAGTTGAAAAACTGTGTCAATTTCATTAACTTGAAAAGATGAGCATAAATTGGGAGAGA
GAGAGAGAGACAAAGATTTTGAATTGAGGTTTAACGGTAAAACACACAAAACCTATTCCCCT
CTGTTTCCAATTTTCATCTAAACAAACAGGTACATATTTGAATGTAATATTGTATACAGACCAGG
GGTAAAACAGGAACTAAAGAAGGCTAACAATCGAGTCGAACCCTCTATGTGAAGCCACAGGT
TTAGTGCAAATTGTAATAAGTTGTTCAGAGAGACTCTTGACTGAAACAAATTGTGAAGCAGAT
TCGATTTTAAAATCAAAATTTGAGTGTCGAGCGGGAAAGTAAAAGTTCCGCTCCAATCTTCTA
ATCTTTTCGTATCTAGCGGGAAATTTCTCAGCAGGTGACTTTCATAATCGCAGTTTTCGTCGATT
CTCTTTTCCGATTTTACGATTCCTCTCTCTCTCTCATGGTGCGATT**G**CT**G**CAGCAGTAAAAATC
AATGCCTTCAAGACTTGAAGAGGAACTTAGAAGGAGACTTACTGAACCTGCGAGAACCAAG
CATCGCGTTACCAGGTCACAACCTCGGAATCAAACTGGTATCTTAAATCTGCTTTCTCTTTCAA
TTTTTACTTCTGATTTTACCCAGAATTTTAGGTTTTTTATTTCGATTTTGTTAACCCTAGATTTCG
AATCTGAAATTTGTAGATGCCGCCGGTGCTTCATCTTCTCAGGCGGCAGGTCCAACTACGGTA
CGGCATCTTTTTCCGTCTTAGGGTTTCCAATGTTTCTTCCTTTTATCGTTATGATCAAATTTGTTT
ATCTATCGAAATTGAAGACCCCGACAAGGAGAGGCGGTGAAGGTGGAGATAATACTCAACAA
AGTGAGTTTTTATATTTGAAGTCTTTTTTTTCCCTCTTTTCATCTCTTTTGTTTGTGAAGTTATT
CTTTTGTAACATCTGCAGCAAATCCTACAACTTCACCAGCTACTGGTACAAGGGTAAGATTTTT
GTGACCATTGCTTATGAACTGCTTCAACTTTGATTTCGTTATTAAGCTGACAAAATTCTCGTTTT
GGTTtcgccattgatttttactgctggagaaaactagt
